# Comprehensive analyses of A 12-metabolism-associated gene signature and its connection with tumor metastases in clear cell renal cell carcinoma

**DOI:** 10.1186/s12885-023-10740-6

**Published:** 2023-03-23

**Authors:** Diaoyi Tan, Daojia Miao, Chuanyi Zhao, Jian Shi, Qingyang Lv, Zhiyong Xiong, Hongmei Yang, Xiaoping Zhang

**Affiliations:** 1 Department of Urology, Union Hospital, Tongji Medical College, Huazhong University of Science and Technology, Wuhan, 430022 China; 2 Institute of Urology, Union Hospital, Tongji Medical College, Huazhong University of Science and Technology, Wuhan, 430022 China; 3 Department of Pathogenic Biology, School of Basic Medicine, Huazhong University of Science and Technology, No.13 Hangkong Road, Wuhan, 430030 China

**Keywords:** clear cell renal cell carcinoma, tumor metastasis, metabolism reprogramming, bioinformatic analyses

## Abstract

**Background:**

The outcomes of patients with clear cell renal cell carcinoma (ccRCC) were dreadful due to lethal local recurrence and distant metastases. Accumulating evidence suggested that ccRCC was considered a metabolic disease and metabolism-associated genes (MAGs) exerted essential functions in tumor metastases. Thus, this study intends to seek whether the dysregulated metabolism promotes ccRCC metastases and explores underlying mechanisms.

**Method:**

Weighted gene co-expression network analysis (WGCNA) was employed based on 2131 MAGs to select genes mostly associated with ccRCC metastases for subsequent univariate Cox regression. On this basis, least absolute shrinkage and selection operator (LASSO) regression and multivariate Cox regression were employed to create a prognostic signature based on the cancer genome atlas kidney renal clear cell carcinoma (TCGA-KIRC) cohort. The prognostic signature was confirmed using E-MTAB-1980 and GSE22541 cohorts. Kaplan–Meier, receiver operating characteristic (ROC) curve, and univariate and multivariate Cox regression were applied to detect the predictability and independence of the signature in ccRCC patients. Functional enrichment analyses, immune cell infiltration examinations, and somatic variant investigations were employed to detect the biological roles of the signature.

**Result:**

A 12-gene-metabolism-associated prognostic signature, termed the MAPS by our team, was constructed. According to the MAPS, patients were divided into low- and high-risk subgroups and high-risk patients displayed inferior outcomes. The MAPS was validated as an independent and reliable biomarker in ccRCC patients for forecasting the prognosis and progression of ccRCC patients. Functionally, the MAPS was closely associated with metabolism dysregulation, tumor metastases, and immune responses in which the high-risk tumors were in an immunosuppressive status. Besides, high-risk patients benefited more from immunotherapy and held a higher tumor mutation burden (TMB) than low-risk patients.

**Conclusion:**

The 12-gene MAPS with prominent biological roles could independently and reliably forecast the outcomes of ccRCC patients, and provide clues to uncover the latent mechanism in which dysregulated metabolism controlled ccRCC metastases.

**Supplementary Information:**

The online version contains supplementary material available at 10.1186/s12885-023-10740-6.

## Introduction

The incidence of kidney cancer rises annually, up to 2.2% of the overall tumor incidence and 1.8% of the overall tumor mortality in 2020 [1]. Renal cell carcinoma (RCC) presents 90% of kidney cancers and the most common type is clear cell renal cell carcinoma (ccRCC), accounting for approximately 80% of all RCC patients [2, 3]. ccRCC is highly aggressive and does not respond to traditional radiotherapy and chemotherapy, so the fatality of ccRCC increases year by year. Hence, we must deepen our understanding of ccRCC, explore mechanisms of ccRCC metastases, identify reliable biomarkers to accurately diagnose tumors in the early stage, and develop more efficient measures to treat ccRCC patients.

Recently, dysregulated metabolism of cancer has drawn the attention of researchers. ccRCC is considered a metabolic disease since the oncogenic mutations are engaged with comprehensive metabolic pathways, so-called metabolic reprogramming [4]. Such metabolism reprogramming enables tumor cells to generate adequate cellular fundamental components, like DNA, membrane structures, and molecules that module tumor energetics. Otto Heinrich Warburg found that most cancer cells yielded energy predominantly through an inefficient process, aerobic glycolysis [5]. Notably, this phenomenon is more conspicuous in ccRCC than in ordinary tissues [6]. Abnormal lipid metabolism is also outstanding in ccRCC, as substantial lipids accumulate in ccRCC cells and the levels of cholesterol esters and cholesterol, as well as triglycerides within ccRCC cells, are remarkably higher than those within normal tissues [7]. Part of the reason is that the mutations of *SCD1*, *FASN*, and *ACC* lead to the abnormal generation of acetyl-CoA and fatty acid, which then gradually aggregate and form lipid droplets in the cytoplasm [8]. As an amino acid, Tryptophan is heavily utilized in ccRCC. The increased utilization results in immunosuppression, which promotes tumor growth and lowers the sensitivity to interferon-α-based immunotherapies [9]. Taken together, the reprogramming of metabolic pathways in ccRCC is essential for cellular components, energy production, and avoiding immune surveillance.

Metastases are the major barrier to promising clinical outcomes, as more than 90% of cancer-associated death is the result of metastasis diseases. Unfortunately, up to 30% of ccRCC patients were diagnosed with metastases [10]. Hence, the restraining and curing of metastases are the basis of improving cancer outcomes. The success of tumor metastases needs tumor cells invading through, or collaborating with stroma, regulating the tissue microenvironment, escaping immune surveillance, and developing resistance to anti-cancer therapies [11, 12]. So far, tumor metastases are too complex to be fully elucidated and the integration of various expertise across scales like oncology, genetics, bioinformatics, and mathematics, is required to understand the phenomenon systemically and holistically.

It has been reported that metabolic reprogramming can help cancer cells adjust to varying tumor microenvironments during the invasion-metastasis cascade, and sustain metabolic flexibility and plasticity [13, 14]. Moreover, metabolism-associated genes (MAGs) exert indispensable functions in cancer development and progression [15]. Therefore, here in the present study, we seek whether the dysregulated metabolism promotes ccRCC metastases, analyze the interaction between MAGs and the outcomes of ccRCC, and explore the underlying mechanisms. By integrally analyzing GSE105261 and the cancer genome atlas kidney renal clear cell carcinoma (TCGA -KIRC) cohorts with 2131 comprehensive MAGs from kyoto encyclopedia of genes and genomes (KEGG) database [16] and molecular signatures database (MSigDb), the metabolism-associated prognostic signature (MAPS) was constructed and further validated in two independent cohorts, E-MTAB-1980 and GSE22541. The MAPS was positively correlated with clinicopathologic parameters, and could accurately and independently forecast the outcomes of ccRCC patients. Furthermore, functional enrichment analysis showed the MAPS was accompanied by pathways related to dysregulated metabolism, tumor metastases, and immune responses. Besides, it was demonstrated that the MAPS also showed a strong capacity to estimate the sensitivity of target therapies and immunotherapies in ccRCC.

In summary, we built a powerful metabolism-associated prognostic signature that was valuable in forecasting patient prognoses, guiding the therapeutic selection, and offering potential pathways for exploring mechanisms related to ccRCC metastases standing on the point of metabolism reprogramming.

## Result

### Establishing WGCNA and selecting modules containing MAGs that were closely associated with metastatic traits

**Fig. 1:**
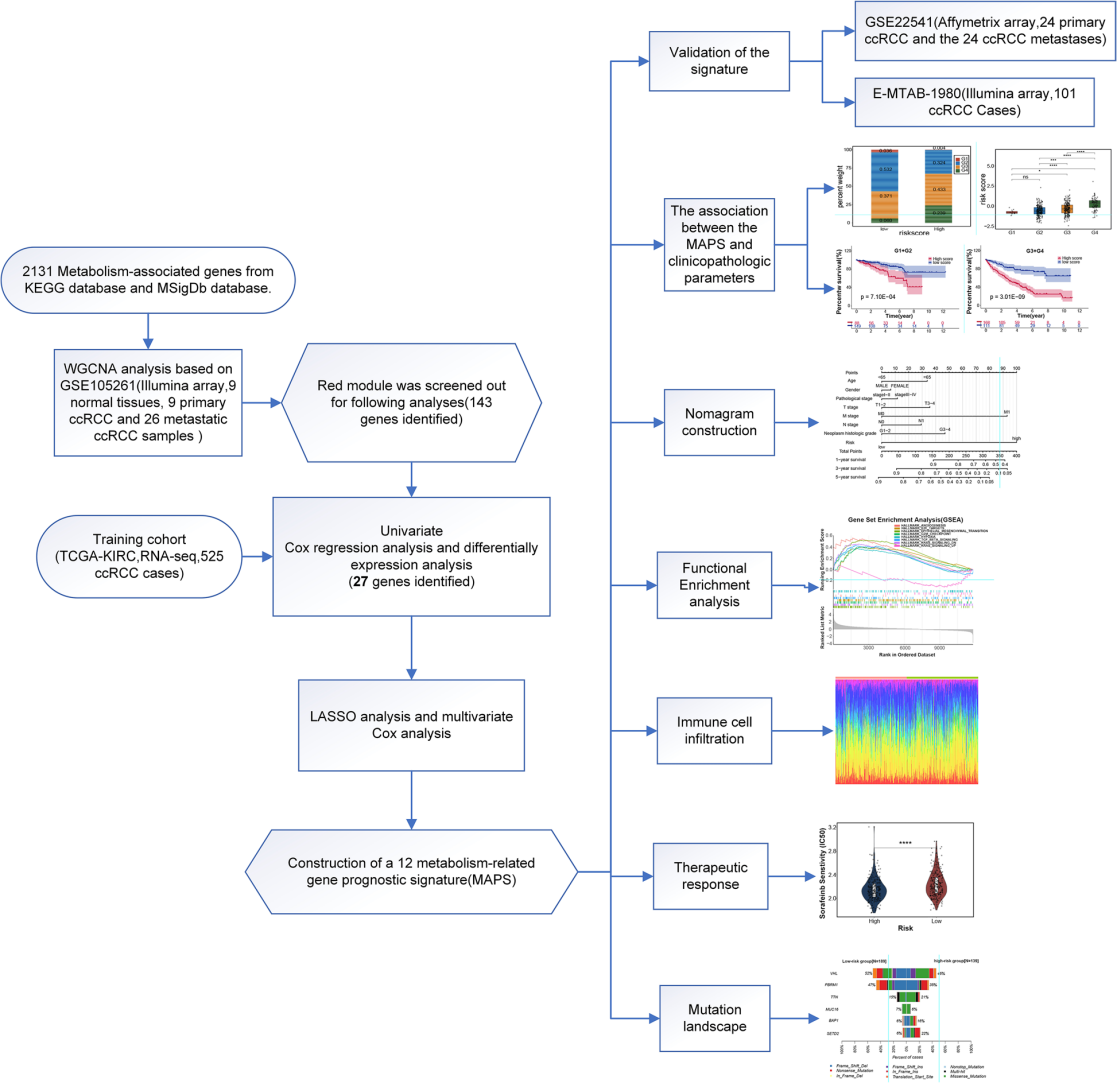
Workflow of the development and validation of the MAPS

**Fig. 2:**
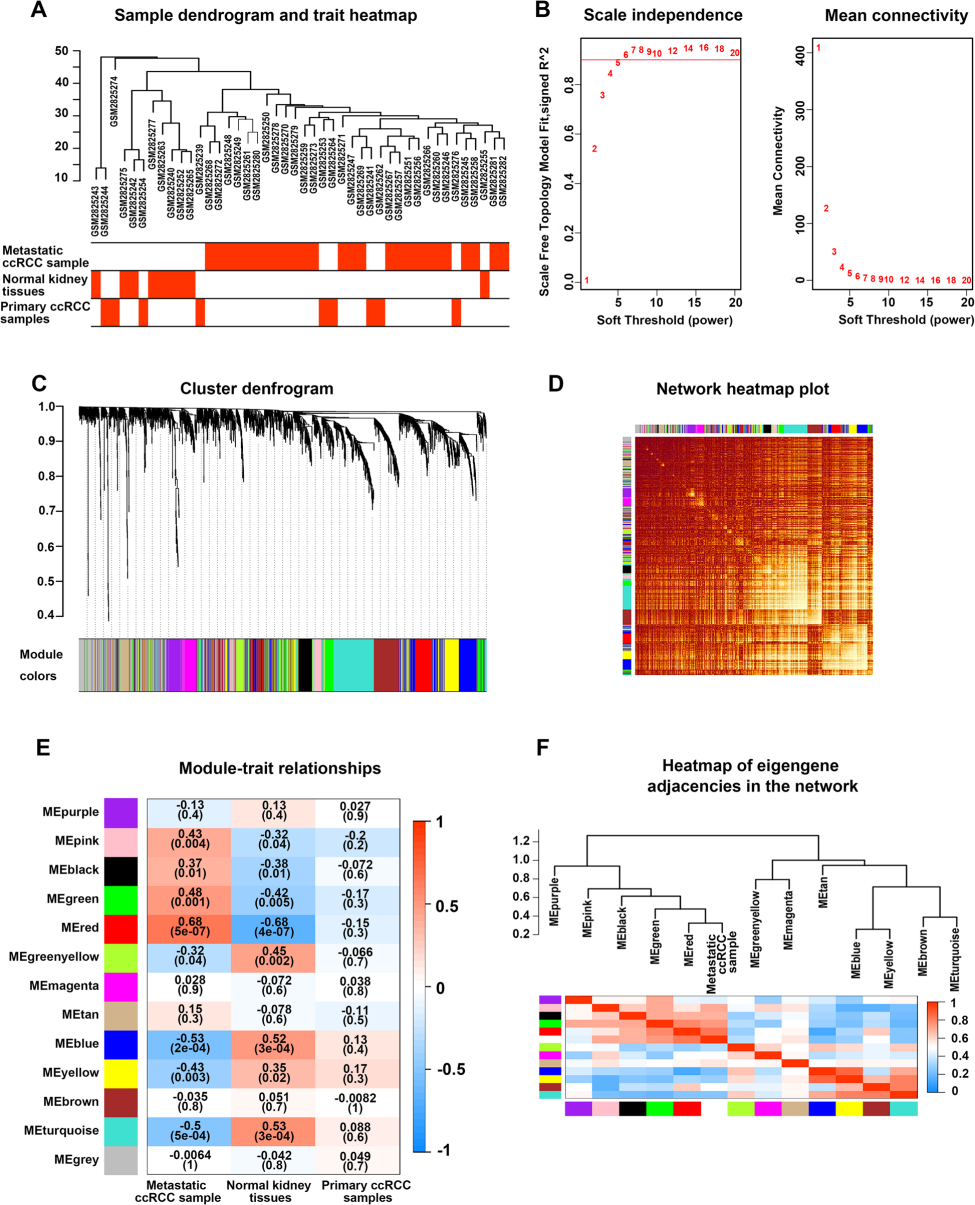
WGCNA construction and identification of modules associated with the clinical traits of ccRCC based on the GSE105261 cohort. **A** Clustering dendrogram of 44 samples from the GSE105261 cohort. **B** Analysis of the scale-free index and the mean connectivity for various soft-threshold powers. **C** Dendrogram of all differentially expressed genes clustered based on the measurement of dissimilarity (1-TOM). The color band shows the results obtained from the automatic single-block analysis. **D** Heatmap depicts the topological overlap matrix (TOM) of genes selected for weighted co-expression network analysis. **E** Heatmap of the correlation between the module eigengenes and histological types of the GSE105261 cohort (metastatic ccRCC, primary ccRCC, and normal kidney tissues). We selected the MEred block for subsequent analyses, as it had the highest association with metastatic ccRCC samples (*r* = 0.68, *P* < 0.01). **F** Heatmap of eigengene adjacencies in the network (color green indicated low adjacency (negative correlation) and color red indicates high adjacency (positive correlation))

The scheme of this study was displayed in Fig. 1. First, a comprehensive MAG set involving 2131 different genes was constructed via KEGG [16] and MSigDb (Supplementary Table S1). The expression profile of the 2131 genes was obtained from the GSE105261 cohort which consisted of the transcriptome profile of 26 metastatic ccRCC samples, 9 primary ccRCC samples, and 9 normal kidney tissues. Then, the transcriptome profile matrix was employed to construct weighted gene co-expression network analysis (WGCNA) [17] and recognize co-expression modules. Pearson’s coefficient was applied to cluster the 44 samples, and the sample clustering tree plus a heatmap showed that all 44 samples were included in the further calculation (Fig. 2A). As the soft-threshold power value was set as 5, the scale-free topology index was 0.9, and both the mean connectivity and scale independence of modules were better (Fig. 2B). Subsequently, 13 co-expression modules were established and displayed in various colors (Fig. 2C), and the interplays within these modules were studied (Fig. 2D). The MAGs in each module were displayed in Supplementary Table S2. Then, we employed the three histopathological types, metastatic ccRCC, primary ccRCC, and normal kidney tissues as clinical traits to study the connections between each module and the development of ccRCC metastases (Fig. 2E). We screened out the red module, which had the highest association with metastatic ccRCC (*r* = 0.68, *P* < 0.01), biologically. In addition, the eigengene dendrogram was applied to detect the connection strength of eigengene adjacency, and the red module was remarkably related to metastatic traits (Fig. 2F). Visualization of the network displayed the connectivity between the red module and the other modules (Supplementary Fig. 1A). The red module includes 143 MAGs (Supplementary Table S3). All of them were applied to reveal the interaction between metastases and the metabolism of ccRCC in the follow-up analyses.

### A MAPS was constructed in TCGA-KIRC cohort

**Fig. 3:**
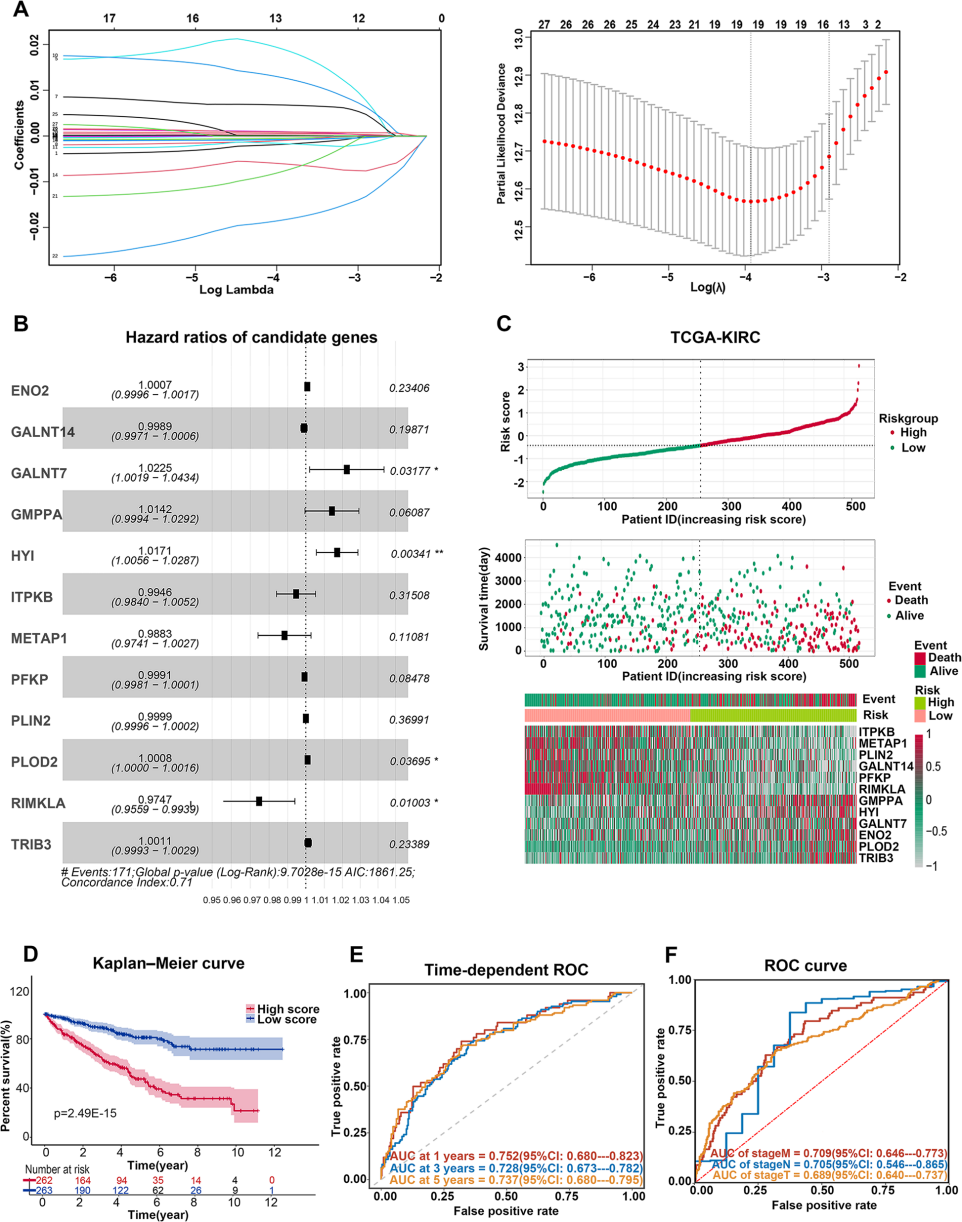
Construction of the MAPS and its prognostic value for ccRCC patients based on TCGA-KIRC cohort. **A** Distribution of LASSO coefficients of the 27 metabolism-associated genes in TCGA-KIRC cohort, and the generated coefficient distribution plots for the logarithmic (lambda) sequence to select the best parameter (lambda). **B** Forest plot demonstrating the multivariable Cox model results of 12 metabolism-associated signature genes using continuous TGCA-KIRC TPM data. **C** Distribution of the risk scores (the upper panel), distribution of prognostic status (the middle panel), and the expression of 12 genes in the MAPS in the TCGA-KIRC cohort (the lower panel). The relative expression of each gene from the MAPS was normalized into Z-score. **D** The Kaplan–Meier survival of the MAPS in the TCGA-KIRC cohort (log-rank test for statistics). **E** The time-dependent ROC curve of a prognostic model for predicting the 1-,3- and 5-year OS of ccRCC patients based on the TCGA-KIRC cohort. **F** The ROC curve of the MAPS for predicting the tumor progresses from stage T1 or T2 to stage T3 or T4, the emergence of lymph node metastases, and remote metastases based on the TCGA-KIRC cohort

One hundred and forty-three genes from the aforementioned red module have been chosen through WGCNA. Univariate Cox regression was run to evaluate genes closely related to the overall survival (OS) of ccRCC patients in TCGA-KIRC cohort. Thirty-one out of 143 genes with *P* < 0.005 were screened out (Table 1). Then, we evaluated whether these genes were differentially expressed among normal samples and tumor samples, or not, in TCGA-KIRC cohort. Among the 31 survival-related MAGs, 27 genes met the threshold set at the absolute value of false discovery rate (FDR) below 0.01, indicating only these 27 genes showed significantly abnormal expression in ccRCC samples (Table 2). Then, least absolute shrinkage and selection operator (LASSO) regression was applied to bypass over-fitting problems. *ENO2*, *GALNT14*, *GALNT7*, *GMPPA*, *HYI*, *ITPKB*, *LPIN3*, *METAP1*, *PFKP*, *PLIN2*, *PLOD2*, *RIMKLA*, and *TRIB3* were preserved when the ideal λ value was determined as 0.055 (Fig. 3A and Supplementary Table S4). Subsequently, employing the multivariate Cox regression, *LPIN3* was deleted from the construction of the signature as its proportional hazard assumption was statistically significant via Schoenfeld's residuals test (Supplementary Table S4). Finally, a 12-gene MAPS was constructed (*ENO2*, *GALNT14*, *GALNT7*, *GMPPA*, *HYI*, *ITPKB*, *METAP1*, *PFKP*, *PLIN2*, *PLOD2*, *RIMKLA*, and *TRIB3*) (Fig. 3B). The full name of each gene and the matching coefficient generated from the multivariant COX analysis was displayed in Table 3. The risk scores were calculated using the linear integration of the relative mRNA levels of these genes weighted by their matching coefficients. As displayed in the scatter chart, patients with high-risk scores ended up with poorer outcomes (Supplementary Fig. 2A). The principal component analysis (PCA) and t-distributed stochastic neighbor embedding (t-SNE) analyses demonstrated dissimilar scattering among the high- and low-risk subgroups (Supplementary Fig. 2B-C). Furthermore, we discovered that the high-risk patients showed worse survival status and shorter survival time in comparison with the low-risk patients (Fig. 3C). Additionally, the heatmap of the 12 genes in TCGA-KIRC cohort manifested that *ITPKB*, *METAP1*, *PLIN2*, *GALNT14*, *PFKP*, and *RIMKLA* was down-regulated in the high-risk subgroup, while *GMPPA*, *HYI*, *GALNT7*, *ENO2*, *PLOD2*, *TRIB3* was up-regulated (Fig. 3C). The Kaplan–Meier analysis demonstrated that high-risk patients displayed inferior survival than low-risk patients (Fig. 3D). Then, the time-dependent receiver operating characteristic (ROC) was employed to check the reliability of the MAPS (Fig. 3E). The area under the ROC curve (AUC) for predicting 1-, 3-, and 5-year OS were 0.752, 0.728, and 0.737, respectively, implying the signature model could reliably distinguish the patients with poor prognosis from the patients with good prognosis. Additionally, the ROC curve demonstrated that the AUC for stage M, stage N, and stage T was 0.709, 0.705, and 0.689, respectively, suggesting that the signature model properly differentiated patients with and without tumor metastases, lymph node invasion, and histological progression (Fig. 3F). Thus, a reliable metabolism-associated prognostic signature was constructed.

**Table 1:** Univariate Cox regression analysis for overall survival

Gene ID	HR	CI	*P*
AK3	0.9868	0.98–0.9938	< 0.001
CHD9	0.9518	0.9332–0.9708	< 0.001
ENO2	1.0019	1.0009–1.0029	< 0.001
ENPP3	0.9987	0.998–0.9995	0.001
GALNT14	0.9971	0.9955–0.9987	< 0.001
GALNT7	1.0297	1.0116–1.048	0.001
GAPDH	1.0001	1–1.0001	0.002
GMPPA	1.0203	1.0091–1.0317	< 0.001
GUCY1A1	0.9918	0.9872–0.9964	< 0.001
HSD3B7	1.0016	1.0005–1.0027	0.003
HYI	1.0196	1.0087–1.0306	< 0.001
ITPKB	0.98	0.9696–0.9905	< 0.001
LPIN3	1.007	1.0032–1.0108	< 0.001
MAN1A2	0.9734	0.9599–0.987	< 0.001
METAP1	0.9691	0.9568–0.9817	< 0.001
NNMT	1.0002	1.0001–1.0004	0.004
PFKP	0.9985	0.9977–0.9993	< 0.001
PGK1	0.999	0.9985–0.9995	< 0.001
PLCB1	0.9601	0.9435–0.9771	< 0.001
PLIN2	0.9995	0.9993–0.9998	< 0.001
PLOD1	1.0014	1.0006–1.0022	0.001
PLOD2	1.0012	1.0007–1.0017	< 0.001
POMGNT2	0.9685	0.9493–0.9881	0.002
PTPRG	0.9708	0.9615–0.9802	< 0.001
RIMKLA	0.9596	0.9435–0.976	< 0.001
RPS15	1.0009	1.0003–1.0014	0.004
S100A10	1.0005	1.0002–1.0008	0.003
SMARCD3	1.028	1.0162–1.0399	< 0.001
SPTLC2	0.9858	0.9781–0.9936	< 0.001
TRIB3	1.0031	1.0018–1.0045	< 0.001
TYMS	0.9911	0.9851–0.9972	0.004

^a^The expression matrix and corresponding clinical information were derived from the TCGA-KIRC cohort

^b^Genes involved in this univariate Cox regression analysis were derived from the red module of WGCNA analysis

^c^*P* lower than 0.005 was set as thresholds

^d^Hazard ratio was estimated from Cox proportional hazard regression model

^e^CI meant confidence interval of the estimated HR

**Table 2:** Differential expression analysis between ccRCC tumor tissue and normal tissue

Gene ID	Log2FoldChange (Log2FC)	Log2CPM	*P*	FDR	Expression Trends
AK3	-1.45	6.36	8.20E-119	8.36E-117	Down
ENO2	3.21	7.52	3.69E-81	1.60E-79	Up
TYMS	2.08	4.77	3.77E-81	1.63E-79	Up
POMGNT2	-1.12	4.11	3.97E-75	1.44E-73	Down
PFKP	1.97	8.92	1.60E-68	4.66E-67	Up
ENPP3	4.59	8.06	3.59E-68	1.04E-66	Up
NNMT	3.94	9.48	7.84E-67	2.17E-65	Up
PLIN2	2.94	9.89	1.38E-53	2.36E-52	Up
GAPDH	1.48	11.98	3.35E-53	5.60E-52	Up
TRIB3	3.14	5.84	7.35E-49	1.02E-47	Up
RIMKLA	2.35	4.92	2.24E-44	2.56E-43	Up
HSD3B7	2.24	6.19	9.25E-42	9.28E-41	Up
ITPKB	-0.95	5.84	2.48E-35	1.89E-34	Down
PLOD1	1.34	8.14	2.49E-33	1.73E-32	Up
PLOD2	1.78	8.31	1.27E-31	8.18E-31	Up
LPIN3	1.57	5.38	1.01E-30	6.28E-30	Up
GMPPA	0.77	5.33	3.99E-25	1.88E-24	Up
PGK1	0.9	9.92	8.25E-25	3.84E-24	Up
S100A10	0.92	8.37	1.70E-23	7.45E-23	Up
RPS15	1.01	8.01	2.34E-22	9.68E-22	Up
SPTLC2	-0.67	6.84	2.34E-22	9.71E-22	Down
HYI	0.86	3.84	2.83E-19	1.01E-18	Up
GALNT14	1.12	7.77	7.19E-18	2.40E-17	Up
GUCY1A1	0.79	6.44	2.37E-13	6.25E-13	Up
MAN1A2	-0.4	6.1	1.24E-09	2.64E-09	Down
METAP1	-0.17	5.42	0.0006511	0.0009494	Down
GALNT7	-0.27	4.37	0.0017783	0.0025001	Down

^a^The differential expression analysis was calculated based on the TCGA-KIRC Counts data via “edgeR” R packages

^b^Expression tread meant the changes in the expression of a particular gene in tumor tissue compared with normal tissue

^c^FDR meant the expected percent of false predictions in the set of predictions

^d^FDR below 0.01 was set as the threshold

**Table 3:** The 12 metabolism-associated prognostic genes obtained from the multivariate Cox regression model

Symbol	Description	Coefficient
ENO2	Enolase 2	0.000651015
GALNT14	Polypeptide N-Acetylgalactosaminyltransferase 14	-0.001139746
GALNT7	Polypeptide N-Acetylgalactosaminyltransferase 7	0.022223137
GMPPA	GDP-Mannose Pyrophosphorylase A	0.014083278
HYI	Hydroxypyruvate Isomerase	0.016940627
ITPKB	Inositol-Trisphosphate 3-Kinase B	-0.005459209
METAP1	Methionyl Aminopeptidase 1	-0.011774191
PFKP	Phosphofructokinase,Platelet	-0.000866611
PLIN2	Perilipin 2	-0.000127161
PLOD2	Procollagen-Lysine,2-Oxoglutarate 5-Dioxygenase 2	0.000801979
RIMKLA	Ribosomal Modification Protein RimK Like Family Member A	-0.025637428
TRIB3	Tribbles Pseudokinase 3	0.001100795

### The MAPS was reliable in both E-MTAB-1980 and GSE22541 cohort

**Fig. 4:**
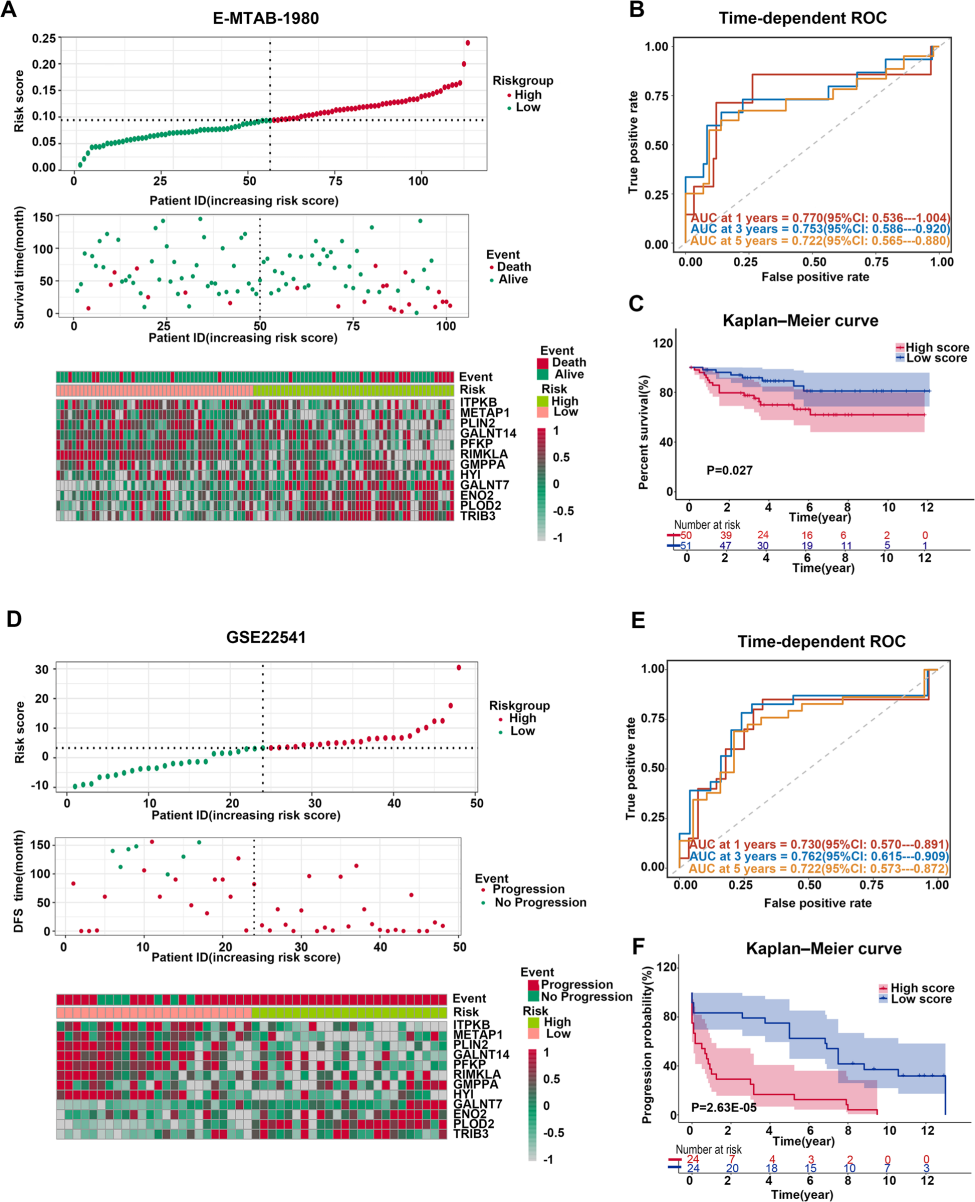
Validation of the MAPS and its prognostic value for ccRCC patients based on the E-MTAB-1980 cohort and GSE22541 cohort. **A** Distribution of the risk scores (the upper panel), distribution of prognostic status (the middle panel), and the expression of 12 genes in the MAPS in the E-MTAB-1980 cohort (the lower panel). The relative expression of each gene from the MAPS was normalized into Z-score. **B**) The time-dependent ROC curve of the MAPS for predicting the 1-,3- and 5-year OS of ccRCC patients based on the E-MTAB-1980 cohort. **C** The Kaplan–Meier survival of the MAPS in the E-MTAB-1980 cohort. **D** Distribution of the risk scores (the upper panel), distribution of prognostic status (the middle panel), and the expression of 12 genes from the MAPS in the GSE22541 cohort (the lower panel). The relative expression of each gene from the MAPS was normalized into Z-score. **E** The time-dependent ROC curve of the MAPS for predicting the 1-, 3- and 5-year DFS based on the GSE22541 cohort. **F** The Kaplan–Meier survival of the MAPS in the GSE22541 cohort. *P* was calculated using the log-rank test (**C**, **F**)

To estimate the practicability and universality of the MAPS, E-MTAB-1980 and GSE22541 cohorts were included as validation cohorts. Patients with poorer status were always with higher risk scores (Fig. 4A and Fig. 4D). The scatter plots also depicted patients with high-risk scores were predisposed to progress and end up with poorer prognosis (Supplementary Fig. 2D and Supplementary Fig. 2G). In the high-risk subgroup, the expression level of *ITPKB*, *METAP1*, *PLIN2*, *GALNT14*, *PFKP*, and *RIMKLA* were lower, while the expression of *GMPPA*, *GALNT7*, *ENO2*, *PLOD2*, and *TRIB3* were higher (Fig. 4A and Fig. 4D). The t-SNE and PCA analyses illustrated disparate distribution among the high- and low-risk subgroups in both E-MTAB-1980 and GSE22541 cohorts (Supplementary Fig. 2E-F and Supplementary Fig. 2H-I). Time-dependent ROC and Kaplan–Meier analyses were also performed. The MAPS manifested satisfactory accuracy for distinguishing the patients with and without poor prognosis according to OS in E-MTAB-1980 and differentiating patients with or without tumor recurrence according to the disease-free survival (DFS) time in the GSE22541 cohort, as the AUC of E-MTAB-1980 cohort at 1-,3- and 5-year OS were 0.770, 0.753 and 0.722, and the AUC of GSE22541 cohort at 1-,3- and 5-year DFS were 0.730,0.762 and 0.722 (Fig. 4B and E). High-risk patients demonstrated poorer prognoses compared with low-risk patients (Fig. 4C) and the DFS in high-risk patients was shorter than that in low-risk patients (Fig. 4F). Additionally, in the E-MTAB-1980 cohort, the ROC curve manifested that the AUC for forecasting remote metastases, the emergence of lymph node invasion and the tumor progresses from stage T1 or T2 to stage T3 or T4 were 0.762, 0.777, and 0.664, respectively, indicating that the MAPS had reliable and universal abilities to predict the progress of tumors (Supplementary Fig. 2J). Taken together, the MAPS was validated in cohorts other than TCGA-KIRC and showed satisfactory practicability and universality.

### The MAPS was closely associated with clinicopathologic parameters and a nomogram was constructed to assist clinical decisions based on the TCGA-KIRC cohort

**Fig. 5:**
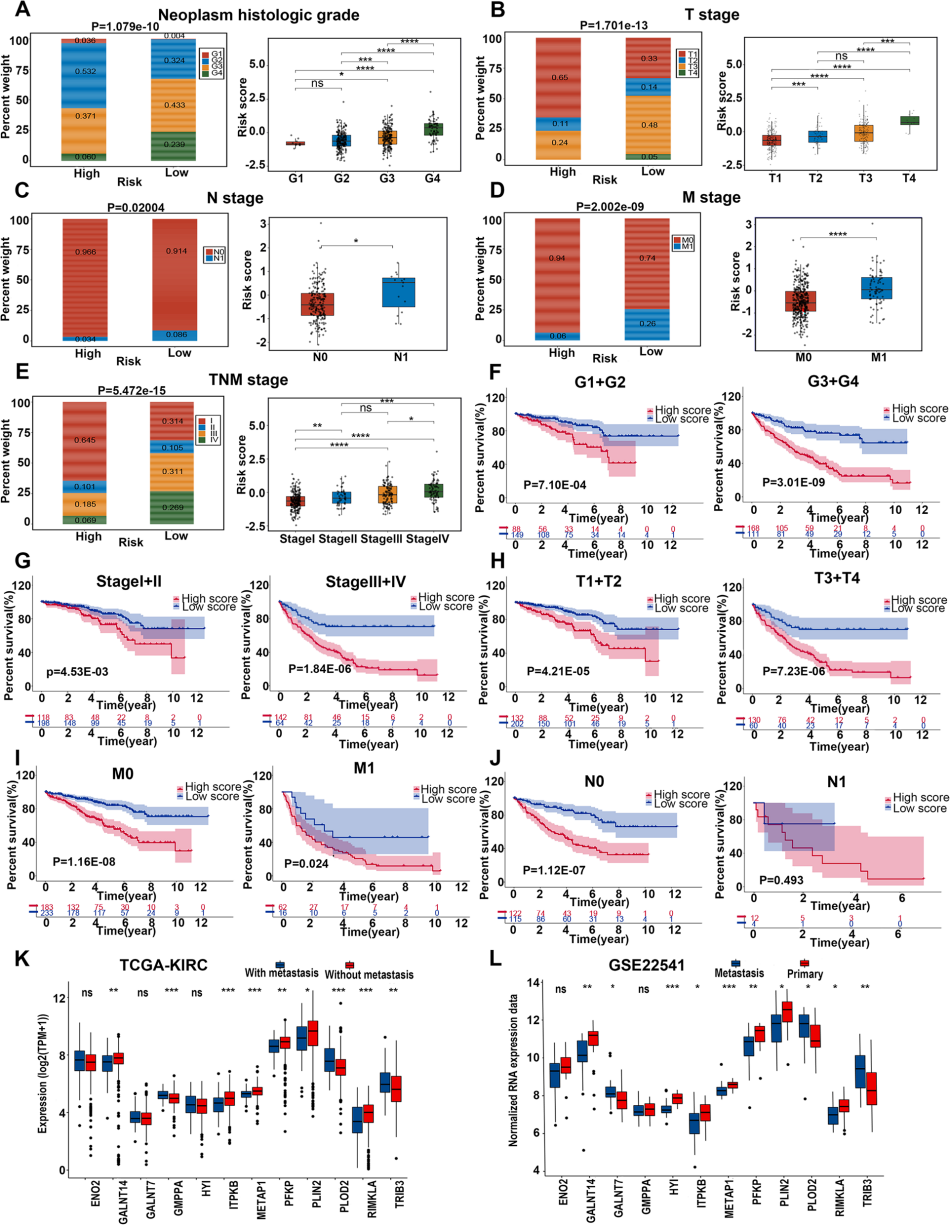
Relationship between risk scores and clinicopathological features. The plots below show the association of risk scores with neoplasm histologic grades (**A**), T stages (**B**), N stages (**C**), M stages (**D**), and TNM stages (**E**) based on the TCGA-KIRC cohort. The Kaplan–Meier analysis of the MAPS regarding the stratified neoplasm histologic grades (**F**), TNM stages (**G**), T stages (**H**), M stages (**I**), and N stages (**J**) based on the TCGA-KIRC cohort. The expression level of 12 genes in the MAPS between samples with remote metastases and samples without remote metastases in the TCGA-KIRC cohort (**K**) and between ccRCC metastatic and primary ccRCC in the GSE22541 cohort(**L**). The gene-level transcription estimates were shown in a form of log2 (TPM + 1) (**K**). *P* was calculated using the χ^2^ test (the left panel of A, B, C, D, and E), one-way ANOVA with Tukey's multiple comparisons tests (the right panels of A, B, C, D, and E), log-rank test (F, G, H, I and J), and two-tailed Mann–Whitney test (K and L). For all the panels, * *P* < 0.05, ** *P* < 0.01, *** *P* < 0.001, **** *P* < 0.0001, "ns" means no significance

The relation between clinicopathologic parameters and the MAPS was assessed utilizing TCGA-KIRC cohort. The high-risk subgroup contained more advanced neoplasm histologic grades(G3 + G4) compared with the low-risk subgroup (Fig. 5A). The proportion of advanced TNM stage in high-risk patients was greater than those in low-risk patients (Fig. 5B-E). Then, we evaluated the scattering of the risk scores across various clinicopathologic parameters. Advanced TNM stage, and neoplasm histologic grade (G3 + G4) had remarkably higher risk scores (Fig. 5A-E). These results demonstrated that the risk scores had a positive relation with advanced clinicopathologic factors. Additionally, the male owned higher risk scores than the female (Supplementary Fig. 3A).

Stratified survival analyses regarding clinicopathological variables were applied in TCGA-KIRC cohort. The signature kept robust predictive ability after ccRCC patients were classified by neoplasm histologic grade, TNM stage, and gender (Fig. 5F-I and Supplementary Fig. 3B). In patients without lymph node metastases, the high-risk subgroup had inferior OS than the low-risk subgroup (Fig. 5J). Nevertheless, no significant variation was witnessed among the high- and low-risk patients with lymph node metastases (Fig. 5J, *P*=0.493). Part of the reason can be explained by the small number of samples with lymphatic metastases in TCGA-KIRC cohort, influencing the accuracy of survival analyses.

As the genes in the MAPS were screened out from the significant metastasis-associated module of WGCNA, we next compared the expression of 12 genes between samples with tumor metastases and without tumor metastases in TCGA-KIRC cohort and between metastatic tissues and primary tumors in GSE22541 cohort. It was manifested that the expression levels of 8 out of 12 genes in TCGA-KIRC cohort and 10 out of 12 genes in GSE22541 cohort had significant differences between subgroups, and the dual changed genes in both cohorts have the same variation tendency (Fig. 5K-L).

The univariate Cox regression demonstrated that apart from the age, TNM stage, and neoplasm histologic grade, the MAPS was also strongly associated with OS (Table 4). Then, the multivariate Cox regression displayed that the MAPS was able to function as an independent prognosticator for patient outcomes (Table 4). Furthermore, the univariate Cox and multivariate Cox regression analyses for progress-free survival (PFS) also indicated that the MAPS was related to PFS and could independently predict the progression of diseases (Table 5).

**Table 4:** Univariate Cox regression and multivariate Cox regression analyses for over survival of KIRC

Variables	Univariate regression			Multivariate regression		
	HR	CI	*P*	HR	CI	*P*
TNM Stage						
I and II						
III and IV	3.93	2.85–5.42	< 0.001	1.15	0.46–2.89	0.764
T Stage						
T1 or T2						
T3 or T4	3.2	2.36–4.35	< 0.001	1.69	0.75–3.78	0.204
N Stage						
No						
N1	3.4	1.8–6.4	< 0.001	1.3	0.66–2.56	0.443
M Stage						
M0						
M1	4.36	3.19–5.97	< 0.001	2.32	1.38–3.93	0.002
Gender						
Female						
Male	0.96	0.7–1.31	0.774			
Age (years)						
< 65						
≥ 65	1.65	1.22–2.23	0.001	1.37	0.91–2.07	0.135
the MAPs						
Risk Score	2.73	2.25–3.31	< 0.001	2.04	1.58–2.63	< 0.001

^a^Overall survival (OS) refers to the time which begins at diagnosis (or at the start of treatment) and up to the time of death

^b^The variables in which *P* was below 0.05 in univariate Cox regression were involved to the further multivariate Cox regression

^c^Hazard ratio was estimated from Cox proportional hazard regression model

^d^Clinical information was derived from the TCGA-KIRC cohort

^e^CI meant confidence interval of the estimated HR

**Table 5:** Univariate Cox regression and multivariate Cox regression analyses for progress-free survival of KIRC

Variables	Univariate regression			Multivariate regression		
	HR	CI	*P*	HR	CI	*P*
TNM Stage						
I and II						
III and IV	6.69	4.66–9.59	< 0.001	2.95	1.19–7.31	0.019
T Stage						
T1 or T2						
T3 or T4	4.41	3.18–6.11	< 0.001	1.11	0.55–2.25	0.764
N Stage						
No						
N1	3.61	1.86–7.03	< 0.001	0.94	0.45–1.94	0.863
M Stage						
M0						
M1	8.7	6.27–12.07	< 0.001	4.1	2.4–7.01	< 0.001
Gender						
Female						
Male	1.54	1.09–2.19	0.015	1.21	0.78–1.89	0.396
Age (years)						
< 65						
≥ 65	1.17	0.85–1.61	0.341			
the MAPs						
Risk Score	2.7	2.18–3.34	< 0.001	1.89	1.4–2.55	< 0.001

^a^Progress-free survival (PFS) is the length of time during and after the treatment of a disease, such as cancer, that a patient lives with the disease but it does not get worse

^b^The variables in which *P* was below 0.05 in univariate Cox regression were involved into the further multivariate Cox regression

^c^Hazard ratio was estimated from Cox proportional hazard regression model

^d^Clinical information was derived from the TCGA-KIRC cohort

^e^CI meant confidence interval of the estimated HR

**Fig. 6:**
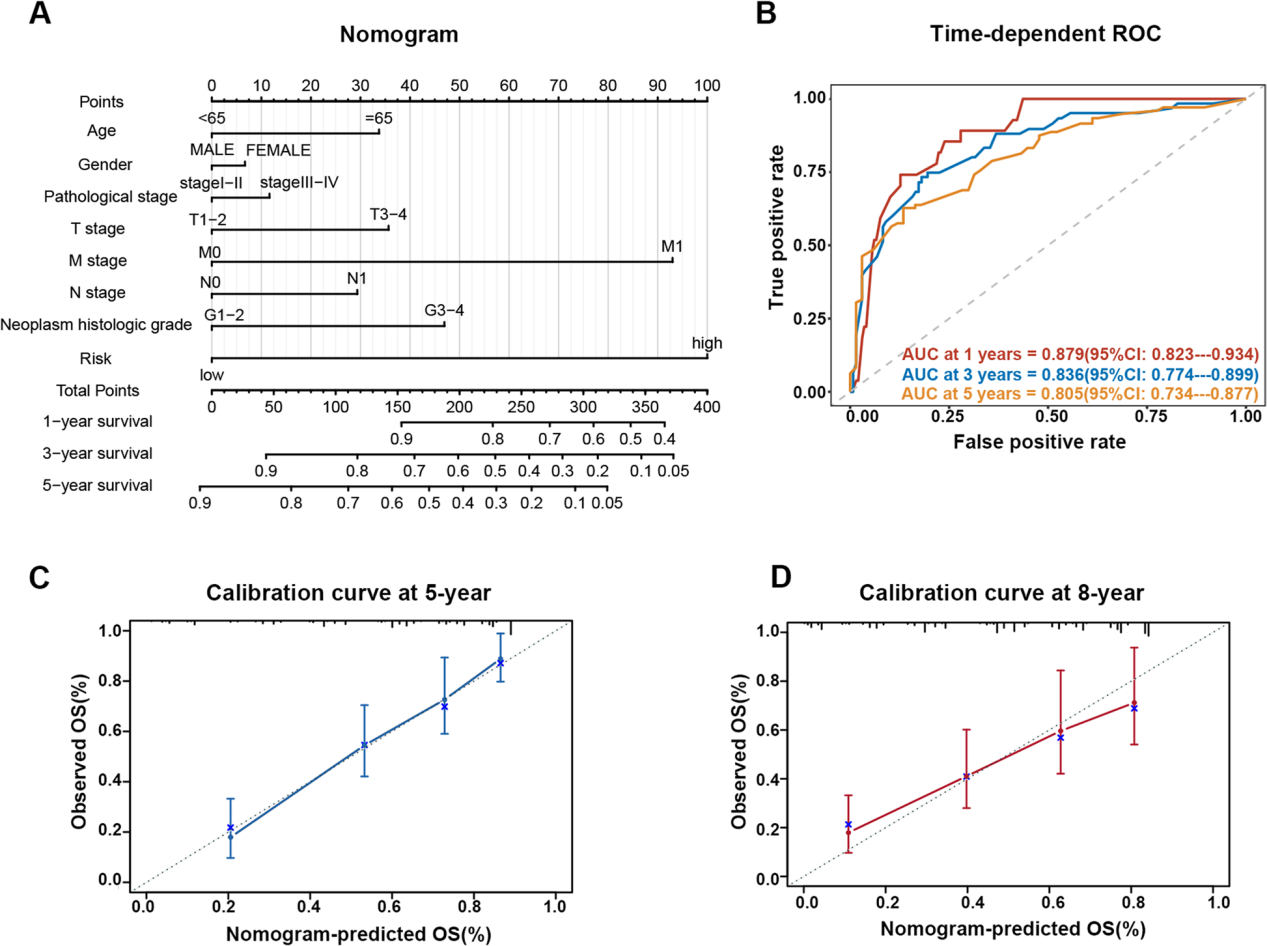
The nomogram to predict the OS of ccRCC. **A** The nomogram for predicting the 1-, 3-, or 5-year OS of ccRCC patients. **B** The time-dependent ROC curve of the nomogram model for predicting the 1-,3- and 5-year OS of ccRCC patients based on TCGA-KIRC cohort. Plots depict the calibration of the nomogram between predicted and observed 5- (**C**), and 8- (**D**) year outcomes

Next, we built a nomogram using the MAPS and the aforementioned clinicopathologic parameters to synthetically forecast the outcomes of ccRCC patients (Fig. 6A). The C-index of this nomogram model was 0.77, which indicated that the nomogram owned satisfactory reliability. Time-dependent ROC analysis showed the AUC for 1-, 3- and 5-year OS were 0.879, 0.836, and 0.805, respectively, implying the nomogram model owned strong credibility of differentiating patients with and without poor prognosis (Fig. 6B). The calibration plot at 5- and 8- years exhibited outstanding congruence between practical information and prognostications from the nomogram (Fig. 6C-D). These results implied that the constructed nomogram possessed advanced accuracy for predicting the outcomes and could assist clinical decisions for ccRCC patients.

### Functional enrichment analysis revealed a tight link between the MAPS and metabolic process, tumor metastases, and immune responses

**Fig. 7:**
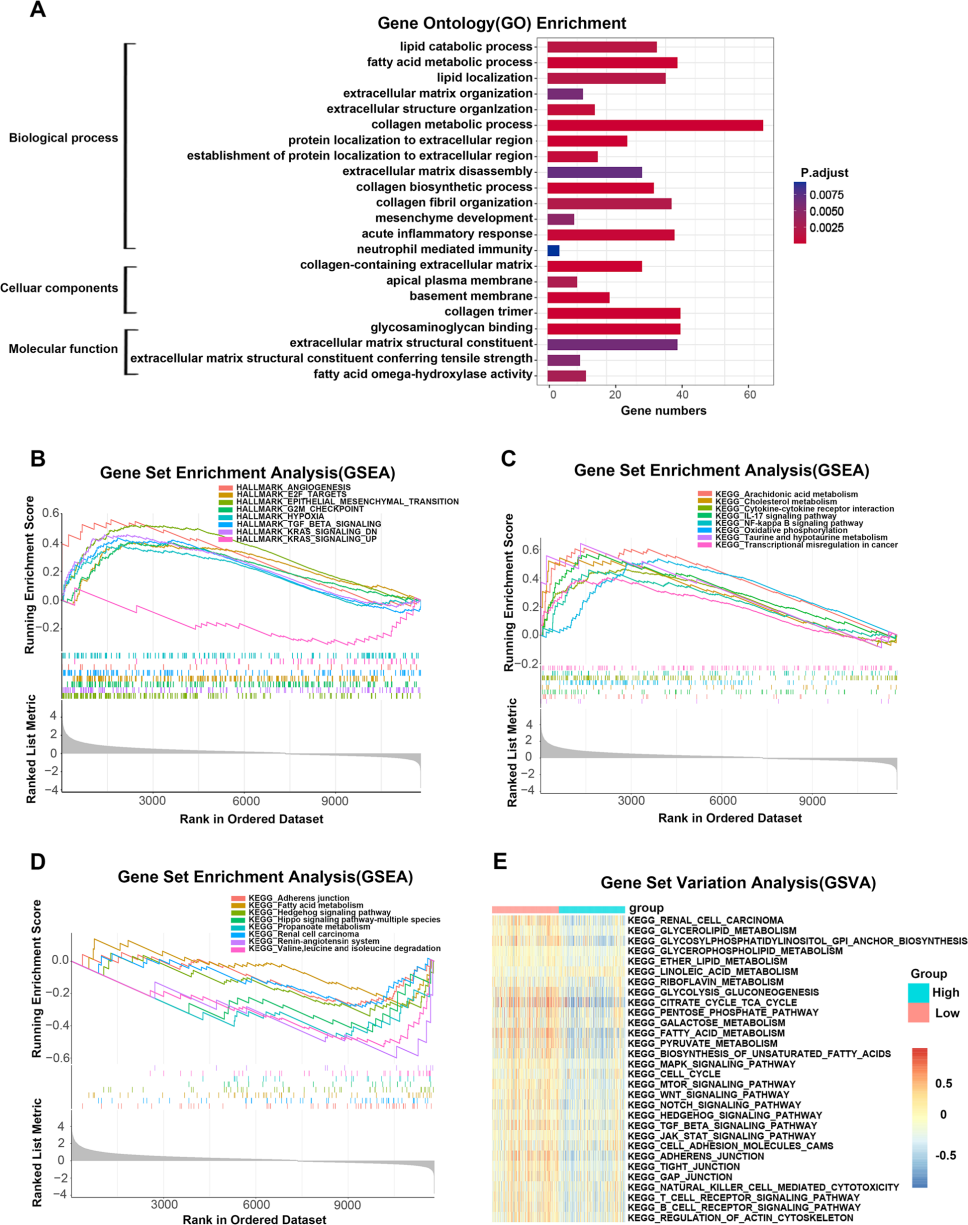
Regulatory network and functional enrichment analysis of the MAPS. **A** GO enrichment analyses of the differentially expressed genes between the high- and low-risk subgroups based on TCGA-KIRC cohort. GSEA was performed using the HALLMARK collection (**B**) and KEGG collection [16] (**C**, **D**). (**E**) GSVA between the high- and low-risk subgroups

We then deeply explored the MAPS and acquired its biological roles. In the previous analyses, we divided the patients from the TCGA-KIRC cohort into high- and low-risk subgroups. Then, the “edgeR” R package was employed for differential expression analyses between those two subgroups based on RNA-Seq HTseq counts and we set the *P* < 0.05 and the logFC > 1 as the thresholds to pick out statistically significant genes for further gene ontology (GO) enrichment analyses. As shown in Fig. 7A, these genes were mostly enriched in lipid-metabolism-associated pathways, extracellular matrix-associated pathways, and immunity-associated pathways (*P* < 0.05), which indicated that the MAPS were closely accompanied by the alteration of metabolism, metastasis process, and immune responses.

Subsequently, we applied gene set enrichment analysis (GSEA) among the high- and low-risk subgroups based on TCGA-KIRC cohort to further delve into the latent mechanisms of the MAPS. Hallmark pathways exhibited that cell cycle pathways (E2F targets, G2M checkpoint), Angiogenesis, Epithelial mesenchymal transition (EMT), and Hypoxia were enriched in the high-risk subgroup, while the TGF-beta signaling was enriched in the low-risk subgroup (Fig. 7B). The KEGG pathways demonstrated that in the high-risk subgroup, metabolism pathways (like arachidonic acid metabolism and cholesterol metabolism) were enriched (Fig. 7C). In the low-risk subgroup, the enriched KEGG terms mostly concentrated on several metabolism pathways (like Fatty acid metabolism and Propanoate metabolism), various key signal pathways (Hedgehog signaling pathway, Hippo signaling pathway, and Renin-angiotensin system), metastasis-related pathway—Adherens junction pathway and cancer-related signaling pathways—Renal cell carcinoma (Fig. 7D). Then, gene set variation analysis (GSVA) was operated. Consistent with the previous outcomes, it was shown that KEGG terms involving metabolism pathways, key regulatory pathways, metastasis-associated pathways, and immune-associated pathways were enriched in the high-risk subgroup (Fig. 7E). Collectively, these results provided a broader view for understanding the MAPS and partly clarified the reason why the outcomes of high-risk patients were inferior to that of low-risk patients standing on the viewpoint of molecular biology.

### The tumor immune microenvironment (TIME) was in an immunosuppressive status and the high-risk patients benefited more from specified targeted drugs and immunotherapies

**Fig. 8:**
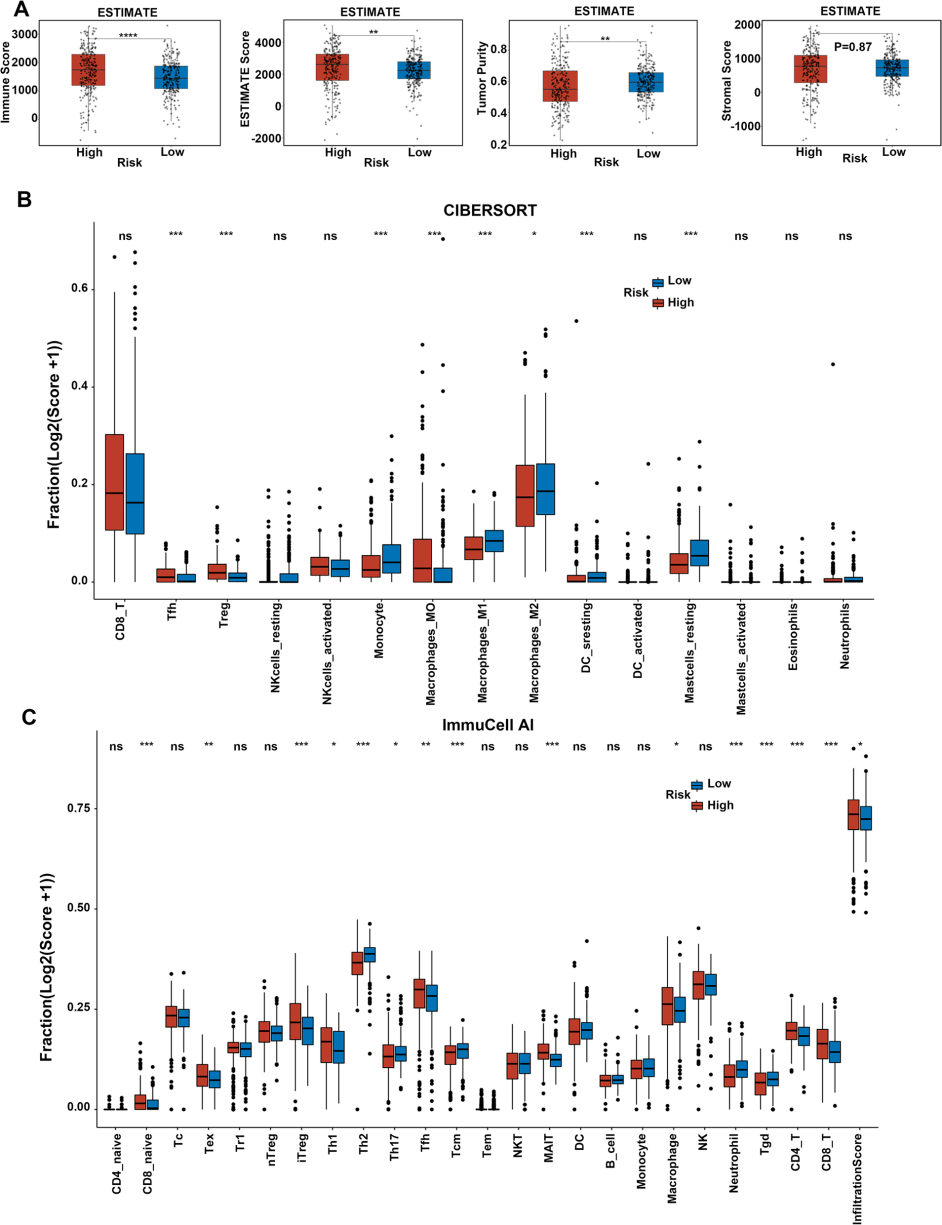
Analysis of the association between risk scores and immune infiltration profiles. **A** Comparison of the immune score, ESTIMATE score, tumor purity, and stromal score between the high- and low-risk subgroups of the MAPS based on the ESTIMATE algorithm. Comparison of the immune cell infiltration between the high- and the low-risk subgroups of the MAPS based on the CIBERSORT algorithm (**B**) and ImmuCell AI algorithm(**C**). The immune cell infiltration scores were shown in a form of log2 (score + 1) (**B** and **C**). *P* was calculated via a two-tailed Mann–Whitney test (A, B, and C). For all the panels, * *P* < 0.05, ** *P* < 0.01, *** *P* < 0.001, **** *P* < 0.0001, "ns" means no significance

**Fig. 9:**
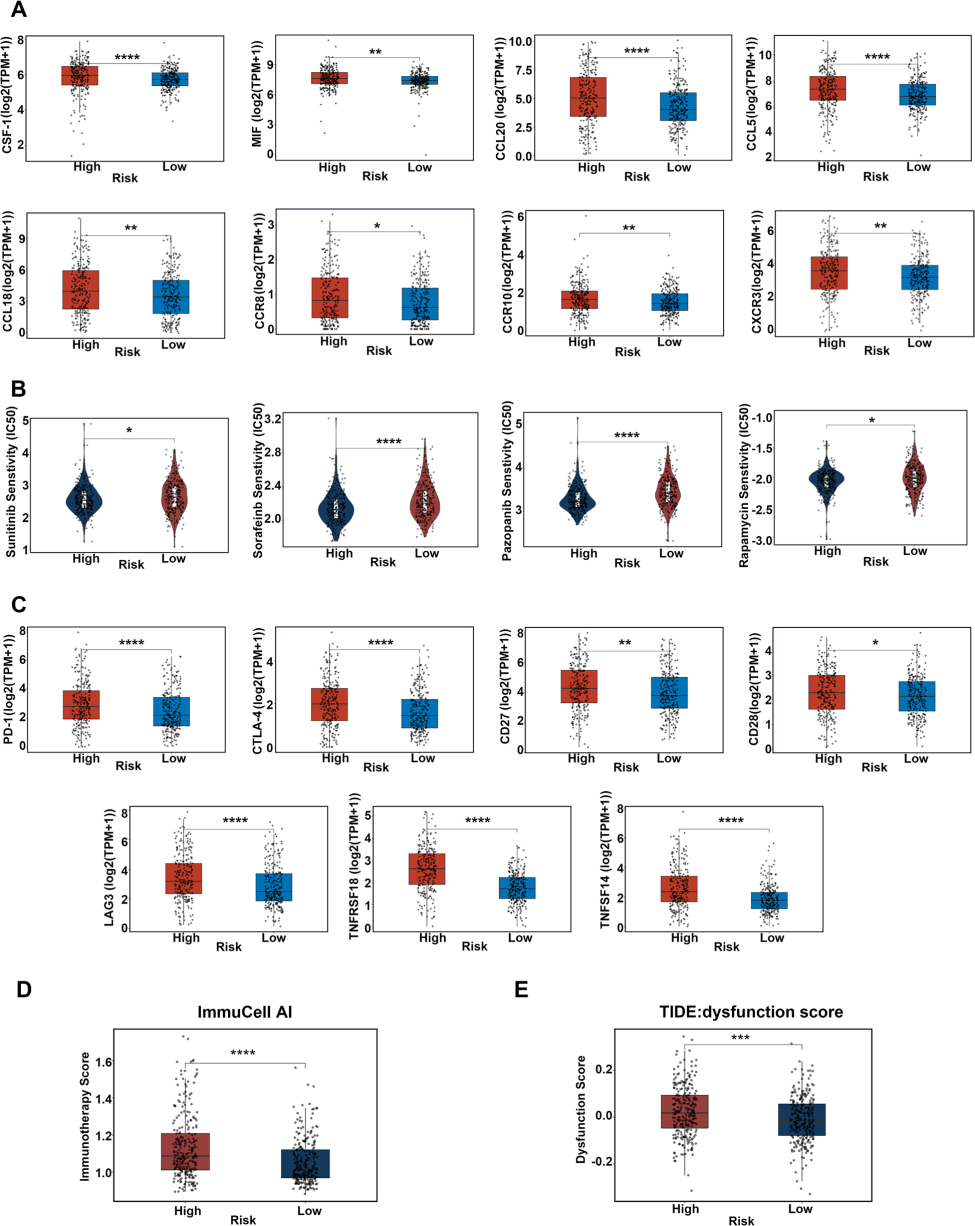
Infiltration of immune cells and drug susceptibility analyses of the MAPS. **A** Comparison of chemokines which are important for the recruitment, differentiation, and activation of macrophages in TIME, and chemokine receptors that promote Tregs cell migration to the TIME between the high- and low-risk subgroups based on the TCGA-KIRC cohort. The gene-level transcription estimates were shown in a form of log2 (TPM + 1). **B** Comparison of drug sensitivity of sunitinib, sorafenib, pazopanib, and rapamycin between the high- and low-risk subgroups of the MAPS. **C** Comparison of the expression of *PD-1*, *CTLA4*, *CD27*, *CD28*, *LAG3*, *TNFRSF18*, and *TNFSF14* between the high- and low-risk subgroups of the MAPS. The gene-level transcription estimates were shown in a form of log2 (TPM + 1). **D** Comparison of the immunotherapy score from ImmuCell AI algorithm between the high-risk subgroup and low-risk subgroup of the MAPS. **E** Comparison of the dysfunction score from the TIDE algorithm between the high-risk subgroup and the low-risk subgroup of the MAPS. *P* was calculated the via two-tailed Mann–Whitney test (A, B, C, and D) and student’s t-test (E). For all the panels, *P < 0.05, **P < 0.01, ***P < 0.001, ****P < 0.0001, "ns" means no significance

The occurrence of metastases partly relied on the elaborate interplay between tumor cells and immune cells at local sites or in systemic circulations. Besides, the previous functional enrichment analyses also demonstrated that several immunity pathways were enriched in the high-risk subgroup. Thus, we aimed to detect the differences of the TIME among the high- and low-risk subgroups, to explore whether the immune responses were involved in the metastatic property of the MAPS or not. Firstly, the ESTIMATE algorithm [18] was conducted, according to TCGA-KIRC transcriptomic profiling. As displayed in Fig. 8A, the high-risk subgroup owned more immune scores and ESTIMATE scores, but less tumor purity in comparison with the low-risk subgroup, indicating the increased immune cell infiltration in the TIME of the high-risk subgroup. The stromal scores showed no statistical significance among the high- and low-risk subgroups (Fig. 8A). Furthermore, immune scores from the xCell algorithm [19] based on the TCGA-KIRC cohort were higher in the high-risk subgroup than those in the low-risk subgroup (Supplementary Fig. 4A). These results implied the TIME of the high-risk patients altered dramatically, and both immune-related cells and molecules were boosted in the high-risk patients. Next, we assessed the types of immune cells within the TIME of ccRCC through two independent algorithms, CIBERSORT [20] and ImmuCell AI [21] based on TCGA-KIRC RNA-seq data. As shown in Fig. 8B-C, the TIME of high-risk subgroup comprised more immunosuppressive cells like regulatory T cells (Tregs), Macrophages, and T follicular helper (Tfh) cells compared with that of the low-risk subgroup, while the infiltration of NK cells showed no clear differences among the two subgroups. Additionally, the data from ImmuCell AI exhibited the immunosuppressive exhausted CD8^+^ T cells were upregulated and pro-inflammatory Th17 cells were downregulated in high-risk subgroups. Chemokines like CCL3, colony-stimulating factor-1 (CSF-1), macrophage migration inhibitory factor (MIF), CXCR4, CCL20, and CCL5 as well as CCL18 are important for the recruitment, differentiation, and activation of macrophages in TIME, and CXCR3, CCR8, and CCR10 are chemokine receptors promoting Treg migration to the TIME [22, 23]. All the aforementioned molecules were elevated in high-risk subgroups, which accounted for the enrichment of macrophages and Tregs in high-risk subgroups (Fig. 9A and Supplementary Fig. 4B). Collectively, these results implied an immunosuppressive phenotype existing in the TIME of high-risk patients.

A considerable proportion of patients with ccRCC are in advanced stages when getting the initial diagnosis and need additional adjuvant and neoadjuvant therapies apart from surgeries. Technically, as a radio-resistant tumor, anti-angiogenesis drugs and immune checkpoint inhibitors (ICIs) are the major and effective alternatives for ccRCC patients [24]. The sensitivity of chemotherapeutic drugs and targeted therapeutics which were usually used in the treatment of ccRCC patients was estimated based on the pRRophetic algorithm [25] based on transcriptomic profiling data of TCGA-KIRC. The high-risk patients showed higher sensitivity to sunitinib, sorafenib, pazopanib, and rapamycin in comparison with the low-risk patients (Fig. 9B). No discrepancies in the sensitivity to temsirolimus and axitinib were identified among the high- and low-risk subgroups (Supplementary Fig. 4C).

Compared with tyrosine kinases inhibitors (TKI) and mammalian target of rapamycin (mTOR) inhibitors, ICIs are more efficient and used as an ideal therapeutic method in metastatic RCC [26]. The adequate expression of immune checkpoint genes reflects a better therapeutic effect to ICIs [27]. Thus, we aimed to detect the expression levels of immune checkpoint genes in ccRCC among the high- and low-risk subgroups, according to the TCGA-KIRC cohort. The expression of *PD-1*, *CTLA4*, *CD27*, *CD28*, *LAG3*, *TNFRSF18*, and *TNFSF14* was significantly higher in the high-risk subgroup compared with the low-risk subgroup, implying that ICIs might exert efficient functions in high-risk patients (Fig. 9C). Then, ImmuCell AI was redeployed to evaluate the response to immunotherapies among low- and high-risk patients. It was exhibited that the high-risk patients owned higher immunotherapy scores and benefited more from immunotherapies (Fig. 9D). Besides, the tumor immune dysfunction and exclusion (TIDE) was conducted to conclude the dysfunction score, a computational method to analyze the infiltration of cytotoxic T lymphocytes (CTL), using TCGA-KIRC RNA-seq data [28]. Jiang et al. demonstrated that in ccRCC, higher dysfunction scores were related to poorer power of cytotoxic T cells killing cancer cells [28]. As shown in Fig. 9E, the dysfunction scores of the high-risk subgroup were higher than that of the low-risk subgroup, in concert with the notion that the high-risk subgroup possessed more progressive tumors.

### The high-risk patients bore higher levels of tumor mutation burden (TMB) in ccRCC

**Fig. 10:**
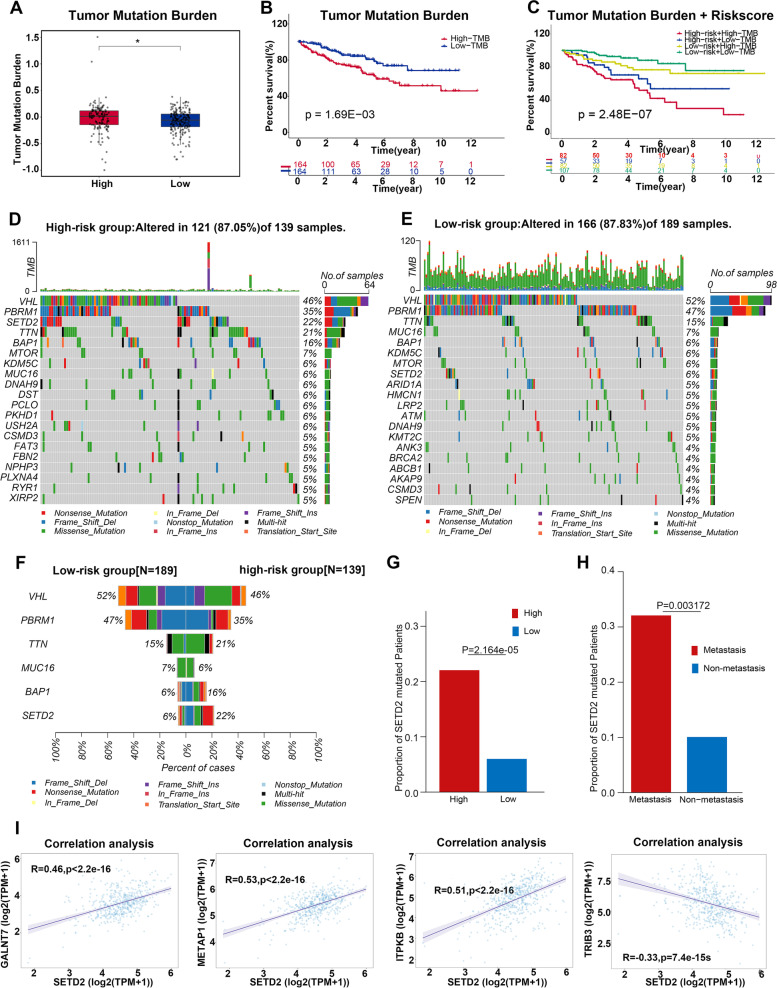
Somatic variant analysis of the MAPS. **A** Comparison of tumor mutation burden (TMB) between the high and low-risk subgroups based on the MAPS. **B** Survival analysis of the different subgroups stratified by TMB. **C** Survival analysis of distinct subgroups stratified by both TMB and the MAPS. **D**-**E** Waterfall plot of common tumor somatic mutation in the high-risk subgroup and low-risk subgroup. **F** Top 5 differentially mutated genes between high- and low-risk subgroups. **G** Comparison of *SETD2* mutated patients between the high- and the low-risk subgroups based on the MAPS. **H** The proportion of *SETD2* mutated patients between the metastasis subgroup and the non-metastasis subgroup based on the MAPS. I the correlation between *SETD2* and *GALNT7*, *METAP1*, *ITPKB*, and *TRIB3* based on the TCGA-KIRC cohort. *P* was calculated via the two-tailed Mann–Whitney test (**A**), log-rank test (**B** and **C**), the χ.^2^ test (**G** and **H**) and Pearson's correlation coefficient(**I**). For panel A, *P < 0.05

Tumor metastases are also driven by genetic alteration within cancers. Thus, we evaluated the level of TMB among the high- and low-risk patients. The high-risk patients held a higher TMB (Fig. 10A) and exhibited unsatisfactory survival (Fig. 10B). We then investigated the advantage of integrating the risk score and TMB in forecasting the OS of ccRCC patients. The Kaplan–Meier curve demonstrated that patients with high-risk scores and high TMB possessed shorter survival than those with low-risk scores or (and) low TMP (Fig. 10C). These results implied that the high-risk patients owned remarkable genetic alterations, which were tightly related to the clinical outcomes of patients with ccRCC.

Furthermore, we investigated somatic variates. The common somatic mutation genes in high- and low-risk patients were depicted, respectively **(**Fig. 10D-E). Among all these genes, the mutation rates of *VHL*, *PBRM1*, and *TTN* were at peak (> 10%) both in high- and low-risk patients (Fig. 10F). Notably, the mutated rates of *SETD2* among the high- and low-risk patients were quite different (22% vs. 6%) (Fig. 10F-G). Besides, we identified that *SETD2* presented as a loss-of-function mutation (nonsense mutation, missense mutation, frameshift deletion, etc.) (Fig. 10F) and the expression level of *SETD2* in the high-risk patients is much less than that in the low-risk patients (Supplementary Fig. 5A). The mutation rate of *SETD2* in patients with metastases was remarkably more than that without metastases, indicating a positive association between mutated *SETD2* and ccRCC metastases (Fig. 10H). Additionally, ccRCC patients with mutated *SETD2* were found to have significantly shortened PFS and DFS compared to *SETD2* intact ones implying that mutated *SETD2* might promote ccRCC progression, while both the mutated *SETD2* and *SETD2* intact subgroups possessed similar OS (Supplementary Fig. 5B-D). Taken together, we inferred that mutated *SETD2* might be one of the triggers to promote metastases in high-risk patients. Next, we evaluated the correlation between *SETD2* and the 12 genes of the MAPS. It was shown that *GALNT7*, *METAP1*, and *ITPKB* were positively associated with SETD2 while TRIB3 was negatively related to *SETD2* (*P* < 0.001) (Fig. 10I), implying that *GALNT7*, *METAP1*, *ITPKB*, and *TRIB3* might be involved in the *SETD2*-mediated functions in the high-risk patients. Then, the enrichment of common oncogenic signaling pathways was checked in both high and low-risk subgroups (Supplementary Fig. 5E-F). By comparison, we found that the fraction of samples affected by the WNT pathway was higher in the high-risk subgroup than that in the low-risk subgroup, which indicates that the WNT pathway was outstanding in the high-risk subgroup.

### The genes from the MAPS were differentially expressed in both mRNA and protein levels

**Fig. 11:**
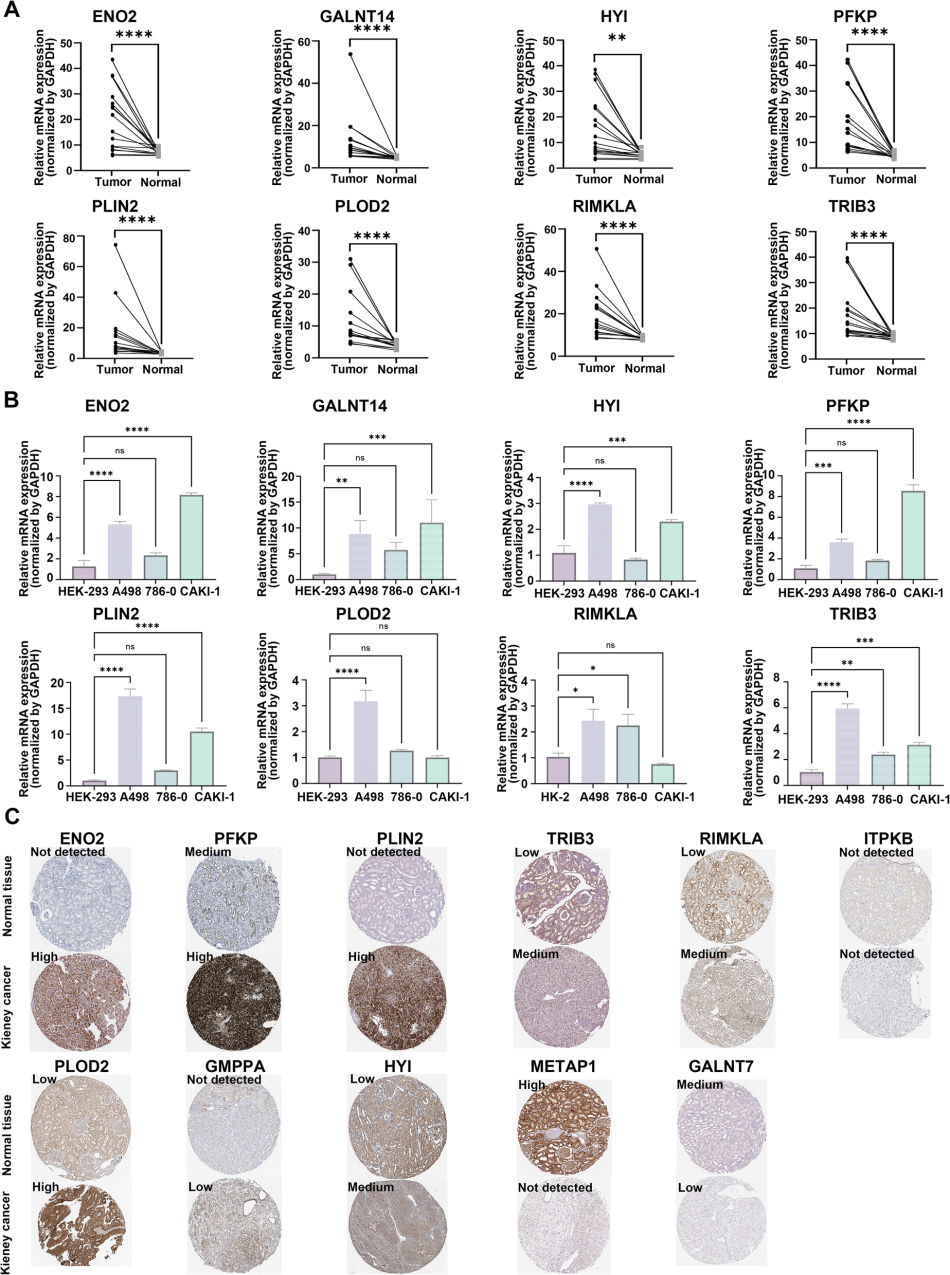
Validation of the 12 genes from the MAPS in clinical samples and cell lines. **A** qRT-PCR assays showed the relative mRNA expression levels of *ENO2*, *GALNT14*, *HYI*, *PFKP*, *PLIN2*, *PLOD2*, *RIMKLA*, and *TRIB3* in sixteen paired ccRCC tumor tissues and corresponding peritumoral normal kidney tissues. **B** qRT-PCR assays showed the relative mRNA expression levels of *ENO2*, *GALNT14*, *HYI*, *PFKP*, *PLIN2*, *PLOD2*, *RIMKLA*, and *TRIB3* in ccRCC cell lines (786–0, A498, and CAKI-1) and the control cell lines (HEK-293 and HK-2). **C** Immunohistochemistry images of ENO2, PFKP, PLIN2, TRIB3, RIMKLA, ITPKB, PLOD2, GMPPA, HYI, METAP1, GALNT7 in kidney cancer and normal kidney tissues. Examples were retrieved from the Human Protein Atlas database. *P* was calculated via the two-tailed Mann–Whitney test(**A**) and one-way ANOVA with Tukey's multiple comparisons test(**B**). For all the panels, *P < 0.05, **P < 0.01, ***P < 0.001, ****P < 0.0001, "ns" means no significance

To confirm the relevant results from bioinformatics, we detected the expression of the 12 genes in the MAPS standing on the perspectives of mRNA and protein levels. According to TCGA-KIRC cohort, the expression of *ENO2*, *PFKP*, *PLIN2*, *TRIB3*, *RIMKLA*, *PLOD2*, *GMPPA*, *HYI*, and *GALNT14* elevated, while the expression of *ITPKB*, *METAP1* and *GALNT7* declined in ccRCC, in comparison with normal kidney tissue (Supplementary Fig. 6A). The quantitative real-time polymerase chain reaction (qRT-PCR) analysis was utilized to determine the mRNA level of these genes in clinical ccRCC samples and ccRCC cell lines. As displayed in Fig. 11A, the mRNA levels of *ENO2*, *GALNT14*, *HYI*, *PFKP*, *PLIN2*, *PLOD2*, *RIMKLA*, and *TRIB3* elevated in ccRCC tissues in comparison with corresponding peritumoral normal kidney tissues. Besides, we detected ccRCC cell lines (786–0, A498, and CAKI-1) and found that the mRNA levels of *ENO2*, *GALNT14*, *HYI*, *PFKP*, *PLIN2*, *PLOD2*, *RIMKLA*, and *TRIB3* raised to varying degrees in ccRCC cells (Fig. 11B). To check the protein levels of these genes, we accessed the HPA database. Results exhibited that the protein levels of ENO2, PFKP, PLIN2, TRIB3, RIMKLA, PLOD2, GMPPA, and HYI were higher in ccRCC, while the protein levels of METAP1 and GALNT7 were lower and the protein level ITPKB remained the same, in comparison with normal kidney tissues (Fig. 11C). Thus, the expression of genes from the MAPS is different in ccRCC compared with normal renal tissues in both mRNA and protein levels.

## Methods

### Data collection

We constructed a comprehensive metabolism-associated gene set involving 2131 genes that were derived from the intersection of comprehensive metabolism genes from the KEGG database ( https://www.genome.jp/kegg/pathway.html) [16] and those from MSigDb ( http://www.gsea-msigdb.org/gsea/login.jsp). Each gene and its corresponding metabolism pathway were shown in Supplementary Table S1.

The row data and corresponding clinical data of GSE22541 and GSE105261 cohorts were derived from the gene expression omnibus (GEO) database via GEOquery R package [29] and data of the E-MTAB-1980 cohort were downloaded from the ArrayExpress database via ArrayExpress R package [30]. The row data of GSE22541 (Affymetrix array) was normalized by applying the robust multi-array averaging algorithm and the normalization of A-MTAB-1980 and GSE105261 (Illumina array) went through bgAdjust background correction, variance stabilizing transformation, quantile normalization and quality control assessment. Besides, RNA-Seq HTseq counts, RNA-Seq HTseq FPKM (fragments per kilo base per million mapped reads), and their matching clinical characteristics were obtained from TCGA database via “TCGAbiolinks” R package [31]. For all cohorts, probe IDs were transformed into gene names according to platform annotation files. When multiple probe IDs pointed to the same gene, average value was used to represent the expression value.

The GSE105261 cohort was combined with the aforementioned 2131 MAGs to conduct WGCNA to sort out metastasis-associated genes which were also related to comprehensive metabolism pathways. The RNA-Seq HTseq counts were employed in differential expression analyses between ccRCC samples and peritumoral normal tissues. The RNA-Seq data in HTseq FPKM format was then converted into TPM as a training cohort since the TPM values were similar to the microarray values [32]. E-MTAB-1980 and GSE22541 cohorts were utilized as validation cohorts. Additionally, the GSE22541 cohort was utilized to detect the expression differences of genes from the signature between primary and metastatic ccRCC samples.

### Establishment of WGCNA and selection of modules closely associated with clinical traits

The expression matrix of 2131 metabolism-associated genes was extracted from the GSE105261 cohort, which contained 26 metastatic ccRCC samples, 9 primary ccRCC samples, and 9 kidney tissue samples. WGCNA package [17] was used to install a gene co-expression network base on this gene set. A sample clustering tree was plotted to rule out the outliers. In this study, the suitable power value for adjacency computation was determined by relying on graphs when the degree of independence was above 0.9. Following, the adjacency matrix was converted into a topological overlap matrix (TOM), an approach to numerically decide clinically significant modules. Each module was a group of genes with notable topological overlap similarity. Thus, co-expression modules and matching eigengenes were acquired. The module-trait relationship was calculated via Pearson's correlation coefficient between the three histopathological types of the GSE105261 cohort and the module eigengenes.

The module with the tightest association with metastatic ccRCC was identified as the pivotal one and picked out for further analyses. In this process, two different values were adopted, gene significance (GS) and module membership (MM). GS stood for the absolute value of the correlation of each trait with expression profile and MM represented the correlation coefficient among the gene expression and module eigengenes.

### Development and validation of the MAPS for ccRCC

A prognostic signature was constructed via the following procedures: First of all, the expression profile of genes in selected modules was screened out from the TCGA-KIRC cohort. Univariate Cox regression was applied to distinguish key genes related to OS (*P* < 0.005). Then, to further filtrate genes closely related to ccRCC, we evaluated differential expression genes among tumors and peritumor benign samples via the “edgeR” package [33, 34] based on TCGA-KIRC counts data, with the threshold set at FDR below 0.01, and then we picked out the significant genes for further analyses. Nest, LASSO regression was exploited via R package “glmnet” [35] to bypass over-fitting problems. Ultimately, multivariate Cox regression was employed to build the gene signature and the Schoenfeld residuals are employed for each variable to check if each variable independently caters to the Cox model assumptions. The risk score was computed based on the relative level of each gene and its matching coefficient from multivariate Cox regression. The formula is:

$$Riskscore=\sum_{j}relative expression level of gene \left(j\right) \times Corresponding Coefficient(j)$$

In the formula, j refers to the number of genes in the constructed signature. PCA and t-SNE were applied to assess the scattering of risk subgroups. Patients were split into low- and high-risk subgroups via the median of the risk score. Through “survminer” R package, we evaluated the survival variation among the high- and low-risk subgroups. Meanwhile, with the “survivalROC” and “timeROC” packages, we assessed the accuracy of the signature for forecasting OS, and the sensitivity and specificity of the signature for predicting the progress. E-MTAB-1980 and GSE22541 were utilized to validate the practicability of the MAPS.

### Construction of the nomogram

The univariate and multivariate Cox regression of the MAPS and clinicopathologic indicators were applied. Then, with the package "rms", a nomogram that contained the MAPS and clinicopathologic indicators was formed. Time-dependent ROC and calibration curves were depicted to detect the effectiveness and stability of the nomogram.

### Enrichment analysis

GO enrichment was employed via R package “clusterprofiler” [36]. Then, GO pathways were plotted via R package “ggplot2”. The same package was employed to perform GSEA for the signature [36]. Next, the R package “GSVA” was utilized to evaluate significantly associated pathways [37]. *P* less than 0.05 was set as statistical significance. “c2.cp.kegg.v6.2.symbols.gmt” and “h.all.v7.2.symbols.gmt” were selected as reference gene sets.

### Assessment of immune cell infiltration

CIBERSORT [20], xCell [19], and ImmuCell AI [21] algorithms were exploited to evaluate the invasion of immune cells. The CIBERSORT.R script is available on the CIBERSORT website. R package "xCELL" was applied for immune invasion analysis. ESTIMATE algorithm presented a tool exploiting transcriptional profiles of tumor tissues to calculate tumor cellularity and the various infiltrations of normal cells. Here, we directly downloaded the stromal score, immune score, and ESTIMATE score from the ESTIMATE website [18] and compare the distribution among different risk subgroups. ImmuCell AI [21] algorithm was applied to assess the effects of immunotherapies as well, and the data was derived from the ImmuCellAI website. TIDE was applied to calculate the infiltration degree of cytotoxic T lymphocytes [28]. All the calculations and analyses mentioned above were based on transcriptomic profiling of TCGA-KIRC.

### Evaluation of the therapeutic Efficacy

The chemotherapeutic response analysis was conducted by R package “pRRophetic” based on the TCGA-KIRC data, according to the genomics of drug sensitivity in cancer (GDSC) [38]. The half-maximal inhibitory concentration (IC50) of every patient was calculated by ridge regression.

### Somatic variants analysis

R package “maftools” was conducted to assess the differences in the somatic mutations [39]. Mutation data were derived from TCGA database. The TMB of patients between subgroups was assessed. Then, the integral mutation status was portrayed in different subgroups. Five commonly mutated genes were compared between subgroups. Common oncogenic signaling pathways were analyzed, too. Additionally, we applied the cBioPortal website(www.cbioportal.org) to estimate the influence of mutated genes on different survival types, like PFS, DFS, and OS [40]. The search term of cBioPortal in this article is kidney renal clear cell carcinoma (TCGA, PanCancer Atlas).

### Cell culture

RCC cell lines (A498,786–0, and CAKI-1) and the control (HEK-293 and HK-2) were derived from the American Type Culture Collection (ATCC, Manassas, VA). DMEM high glucose medium with 10% fetal bovine serum and 1% penicillin–streptomycin was prepared for cell culture and the incubator was set in a 5% CO^2^ at 37^◦^C.

### RNA isolation and qRT-PCR

Sixteen couples of ccRCC tissue and corresponding peritumor normal kidney samples were derived from the Department of Urology, Union Hospital, Tongji Medical College, Wuhan, China. The study followed overall associated ethical rules. The RNA was extracted via TRizol reagent (Thermo Fisher Scientific, Waltham, MA). NanoDrop 2000 spectrophotometer (NanoDrop Technologies, Wilmington, DE) was applied to test the concentration and purity of the above-mentioned extracted RNA. We used 1 µg of extracted RNA for the reverse transcription process and employed the SYBR Green mix (YEASEN, China) to operate Real-time quantitative PCR analysis via StepOnePlus RT-PCR (Thermo Fisher Scientific). GAPDH was utilized as an internal reference to normalize the results. The expression of each gene was calculated by: 2^-ΔCt (ΔCt = Ct_Gene_–Ct_GAPDH_). Gene primers are obtained from TSINGKE Biological Technology (Beijing) and listed in Table 6.

**Table 6:** List of primers for qRT-PCR

Primer	Sequence
**PFKP**	
Forward	5'-CGTAGCTGTCATCAACGTGG-3'
Reverse	5'-TGTGGCGATCTCTTCCAAGT-3'
**HYI**	
Forward	5'-CCCAGGGAGCTGATCGAATA-3'
Reverse	5'-TTCCCATCCATGATCTGCCA-3'
**PLIN2**	
Forward	5'-AGTCTGTGTGTGAGATGGCA-3'
Reverse	5'-CCCAGTCACAGTAGTCGTCA-3'
**RIMKLA**	
Forward	5'-GATGTGGGTGGGATCATTGC-3'
Reverse	5'-TCCCAGTTCAGGCTCACTTT-3'
**GALNT14**	
Forward	5'-CGAAGATGCAAAGTCCCAGG-3'
Reverse	5'-TTTTGGTCCATTGCTGTCGG-3'
**PLOD2**	
Forward	5'-GGGAGTTCATTGCACCAGTT-3'
Reverse	5'-CAGCCTTTTCGTGGTGACTC-3'
**TRIB3**	
Forward	5'-CGTGATCTCAAGCTGTGTCG-3'
Reverse	5'-GAGTATCTCAGGTCCCACGT-3'
**ENO2**	
Forward	5'-GGGCACTCTACCAGGACTTT-3'
Reverse	5'-CAGACAGTTGCAGGCCTTTT-3'
**GAPDH**	
Forward	5'-GAGTCAACGGATTTGGTCGT-3'
Reverse	5'-GACAAGCTTCCCGTTCTCAG-3'

The table contained the primers designed for *PFKP*, *HYI*, *PLIN2*, *RIMKLA*, *GALNT14*, *PLOD2*, *TRIB3*, *ENO2* and *GAPDH*

### Statistical Analysis

In this study, R version 4.1.2 and GraphPad Prism 9.0 were used. Continuous variables were organized via mean and standard error (SD). The comparison of two subgroups of normally distributed variables required the student’s *t*-test, while the comparison of two subgroups of non-normally distributed variables required the Mann–Whitney *U* test. Categorical variables were summarized by proportion (%) and compared between groups using the chi-square test. The log-rank test was used to test whether there were survival differences between two or more groups of individuals in the Kaplan–Meier analysis. One-way analysis of variance (ANOVA) with Tukey's multiple comparisons test was used to compare the means among three or more groups.

## Discussion

As we mentioned before, up to 30% of ccRCC patients were diagnosed with metastases [10]. Metastases are the most lethal aspect of cancer since they are difficult to be cured through surgical resection, conventional chemotherapy, and radiation therapy, with the fact that above 90% of all tumor-associated deaths are caused by metastasis diseases [12]. Also, for patients with ccRCC, due to distant metastases and local recurrence, the outcomes are not satisfactory all the time [41]. Meanwhile, ccRCC is considered a metabolic disease [4], since multiple metabolic pathways are remarkably reprogrammed in ccRCC [42]. Increasing evidence indicated that MAGs exerted essential functions in tumor development and progression [15]. According to "the Warburg effect", glucose metabolism in tumors switches to aerobic glycolysis resulting in the accumulation of lactic acid [43], glutamine [44], and the low-PH tumor microenvironment (TME) [45], which are responsible for promoting the invasion and migration of tumor cells. Additionally, lipid metabolism is also conspicuous in tumors like ccRCC with accumulating evidence showing that the phenotype of ccRCC cells is similar to that of adipotytes [46], and the lipid metabolic reprogramming is tightly related to the emergence of metastases. For example, it was found that peroxisome proliferator-activated receptor gamma coactivator 1-alpha (PGC1A), as a lipid metabolic regulator activated by melatonin, promoted tumor slimming, a lipid browning process reducing catabolic state, and inhibited ccRCC metastases through uncoupling protein 1(UCP1)-dependent manner [47]. Amino acids, like proline, serine, asparagine, and glycine, also affect the emergency of tumor metastases [48–51]. Taken together, we can infer that the metabolic reprogramming in tumors closely affects the occurrence of metastases, while the discussion about this research hotspot in ccRCC is rare. In this article, we create a novel signature, according to the comprehensive MAGs which are also relevant to ccRCC metastases, to help forecast the outcomes of ccRCC patients, assist in choosing treatment regimens, and most importantly provide ideas for unveiling the mechanisms underlying the phenomenon that metabolic reprogramming affects tumor metastases, under the help of high-throughput methods and bioinformatics methodology.

First of all, we downloaded 2131 comprehensive MAGs from the KEGG database [16] and MSigDb. Then through WGCNA, univariate Cox regression, differential expression analysis, LASSO regression, and multivariate Cox regression based on GSE105261 and TCGA-KIRC cohorts, we constructed a 12-gene-metabolism-associated prognosis signature, termed the MAPS by our team. The genes from the MAPS were closely associated with the ccRCC metastasis trait and affected the outcomes of ccRCC patients, and all of them were differentially expressed in ccRCC tumors. Using the median risk score as a cut-off, patients were assigned to low-risk and high-risk subgroups. The high-risk subgroup displayed extraordinarily worse survival. The ROC analyses demonstrated that the MAPS performed satisfactory correctness in forecasting 1-,3-and 5-year OS and the occurrence of progress. Additionally, both multivariate Cox regression and stratified survival analyses regarding clinicopathological parameters implied that the MAPS acted as an independent biomarker. Moreover, E-MTAB-1980 and GSE22541 were utilized to verify the practicability and universality of the MAPS. Taken together, the MAPS was a convincing biomarker for distinguishing the severity and predicting OS in ccRCC patients.

As the genes in the MAPS were screened out via the most metastasis trait-associated module in WGCNA, we further delved into the relationship between the MAPS and metastasis trait. Firstly, we found that the expression levels of these genes from the MAPS alter transcriptionally between primary ccRCC and metastatic samples and may trigger or accelerate the ccRCC metastasies. Moreover, in high-risk patients, the ratio of stage M1 to stage M0 was greater than that in low-risk patients and patients with metastases owned higher risk scores. Besides, The ROC curve illustrated that the AUC for forecasting the emergence of remote metastases was 0.709 in the TCGA-KIRC cohort and 0.762 in the E-MTAB-1980 cohort, respectively. These results implied the close connection between the MAPS and ccRCC metastases and validated that the MAPS had a reliable ability to forecast the emergence of ccRCC metastases. Next, the results from functional enrichment analysis exhibited that the MAPS was closely related to the metastasis trait. On the one hand, the enriched metabolism pathways in GO and GSEA, like fatty acid, arachidonic acid metabolism, cholesterol metabolism, and oxidative phosphorylation metabolism, implied that differentially expressed genes among high- and low-risk subgroups of the MAPS mainly focused on metabolic processes. On the other hand, GO analysis indicated that the MAPS were closely accompanied by the alteration of the metastasis process. Furthermore, GSEA also demonstrated that angiogenesis, Adherens junction, and EMT were enriched in the high-risk subgroup. Targeting the angiogenesis pathway is an attractive approach for cancer therapy, and it is worth mentioning that among tumors, RCC is more sensitive to VEGF inhibitors [52]. Thus, we analyzed the sensitivity of common chemotherapeutic drugs and targeted therapeutics using the pRRophetic algorithm. It was illustrated that the high-risk patients showed more sensitivity to rapamycin, sorafenib, sunitinib, and pazopanib compared with the low-risk patients, while no difference in the sensitivity to temsirolimus and axitinib was observed among high- and low-risk subgroups. Furthermore, we identified that the mutation rate of *SETD2* in patients with metastatic ccRCC is higher than that without metastatic ccRCC and meanwhile *SETD2* mutated more commonly in high-risk patients. The enrichment of common oncogenic signaling pathways also demonstrated the fraction of samples affected by the WNT pathway was higher in the high-risk patients than that in the low-risk patients. These results reinforced that metastasis was an important trait for the high-risk subgroup from the viewpoint of somatic mutation.

Subsequently, we discuss how dysregulated metabolism affects ccRCC metastases. GSEA of the MAPS demonstrated that several key pathways, like Hedgehog signaling (Hh), Hippo signaling, and the Renin-angiotensin system, were enriched. These three pathways are simultaneously implicated in dysregulated metabolism and tumor metastasis. Aberrant Hh, Hippo, and Renin-angiotensin signaling could lead to a series of diseases associated with abnormal lipid metabolism [53, 54], while it can also advance tumor progression and metastases [55, 56]. In the following research, we tested the differences in the TIME among the high- and low-risk subgroups and found that both immune-related cells and molecules were boosted in the high-risk subgroup. Besides, the high-risk subgroup comprised more immunosuppressive cells like Tregs and macrophages, while anti-tumor Th17 cells were significantly downregulated in the high-risk subgroup. The chemokines for the recruitment, differentiation, and activation of macrophages and Tregs in TIME [11] were elevated in high-risk subgroups, which was consistent with the result that macrophages and Tregs were enriched in high-risk subgroups. Tumor-associated macrophages (TAMs) constitute the main tumor-infiltrating immune cells in TIME and can promote tumor cell invasion and metastases [57]. Besides, TAMs are metabolically active [58], and metabolic alterations, like glucose metabolism, lipid metabolism, and glutamine metabolism can determine the functions of TAMs in cancer progression [59]. Interestingly, apart from directly aiming at tumor cells, suppressing Hh signaling could also reconfigure the TIME to be active [55]. Tumor-derived sonic hedgehog (SHH), the hg signaling ligand, acts at TAM to drive M2 polarization which inhibits CD8^+^ T cell recruitment to the TIME and promotes tumor progression [60]. Tregs act as pro-tumor cells in TIME. A higher frequency of Treg cells reflects poorer outcomes in various types of cancer [22]. Taken together, the high infiltration of macrophages and Tregs provides an explanation for the poor outcomes of the high-risk subgroup and brings us thoughts that whether the immune cells are involved in the metabolism-mediated metastases of ccRCC tumor cells, which needs further validation. Besides, checkpoint inhibitors (ICIs) have been effective strategies for ccRCC metastases [61]. We identified that the high-risk subgroup owned higher immune response scores and benefited more from immunotherapy. Thus, the MAPS can serve as an indicator to assist doctors to make clinical decisions in choosing the proper drugs.

Numerous prognostic signatures have been brought up, some of which were tightly related to metabolic abnormalities. For example, Bian et al. have constructed a cuproptosis-related prognostic signature [62]. Their signature was based on the ten known cuproptosis-related genes, while our MAPS was based on 2131 comprehensive MAGs. Notably, the cuproptosis-related gene signature showed poorer performance in predicting the outcomes of ccRCC patients compared with the MAPS [62]. Furthermore, the role of cuproptosis in ccRCC has not been reported and whether it was associated with ccRCC metastases was unknown. M.Alchahin et al. constructed a specific metastatic signature associated with poor prognosis according to the single-cell RNA-seq (scRNA-seq) studies [63]. In contrast, our MAPS was based on the TCGA-KIRC RNA-seq data and their corresponding clinical information. Although both M.Alchahin et al. and this study launched signatures from the perspective of tumor metastases, our MAPS focused on the most notable feature of ccRCC, metabolism reprogramming, and tried to explore the latent mechanism in which dysregulated metabolism controlled ccRCC metastases.

In general, this study offers a novel comprehension of the invasion and metastases of ccRCC from the view of metabolism reprogramming. A12-gene MAPS was successfully constructed with the capacity to independently and accurately forecast the outcomes of ccRCC patients. Evermore, the MAPS owned prominent biological functions and clinical value, such as predicting the emergence of metastases, recognizing ccRCC patients with poor outcomes, and assisting clinical decisions.

## Supplementary Information

**Additional file 1: Supplementary Table S1.** The comphrehensive metabolism gene set.

**Additional file 2: Supplementary Table S2.** The genes and matching information in WGCNA.

**Additional file 3: Supplementary Figure 1.** WGCNA construction and identification of modules associated with the clinical traits of ccRCC based on the GSE105261 cohort. **Supplementary Figure 2.** Construction and validation of the MAPS. **Supplementary Figure 3.** Relationship between risk scores and clinicopathological features. **Supplementary Figure 4.** Infiltration of immune cells and drug susceptibility analyses of the MAPS. **Supplementary Figure 5.** Analysis of the association between risk score and immune infiltration profiles. **Supplementary Figure 6.** Expression ofthe 12 genes in the MAPS in ccRCC and normal kidney tissue in TCGA-KIRC dataset.

**Additional file 4: Supplementary Table S3.** The genes from the red module and their module menbership and trait significance value.

**Additional file 5: Supplementary Table S4.** The 13 genes from LASSO regression and their proportional hazards assuption results in multivariate Cox regression.

## Data Availability

The datasets used in this article were derived from the TCGA database (https://portal.gdc.cancer.gov/), the GEO database (https://www.ncbi.nlm.nih.gov/geo/), the accession number involves GSE105261 and GSE22541) and the EMBL-EBI database (https://www.ebi.ac.uk/biostudies/arrayexpress/studies/, the accession number involves E-MTAB-1980).
