# Supplementary material for: Comprehensive analyses of A 12-metabolism-associated gene signature and its connection with tumor metastases in clear cell renal cell carcinoma
Source: BMC Cancer. 2023 Mar 23;23:264. doi: 10.1186/s12885-023-10740-6 (PMC10035225; doi:10.1186/s12885-023-10740-6)
Supplement: Supplementary file 1 — Additional file 1: Supplementary Table S1. The comphrehensive metabolism gene set. [file 12885_2023_10740_MOESM1_ESM.pdf]

Supplementary Table S1: The comphrehensive metabolism gene set

Gene ID	KEGG	GSEA
GOT1	amino acid metabolism	REACTOME_METABOLISM_OF_CARBOHYDRATES
GOT1L1	amino acid metabolism	Absent
GOT2	amino acid metabolism	REACTOME_METABOLISM_OF_CARBOHYDRATES
IL4I1	amino acid metabolism	HALLMARK_FATTY_ACID_METABOLISM
DDO	amino acid metabolism	REACTOME_METABOLISM_OF_AMINO_ACIDS_AND_DERIVATIVES
ASRGL1	amino acid metabolism	REACTOME_METABOLISM_OF_AMINO_ACIDS_AND_DERIVATIVES
ASNS	amino acid metabolism	REACTOME_METABOLISM_OF_AMINO_ACIDS_AND_DERIVATIVES
NIT2	amino acid metabolism	Absent
GPT2	amino acid metabolism	REACTOME_METABOLISM_OF_AMINO_ACIDS_AND_DERIVATIVES
GPT	amino acid metabolism	REACTOME_METABOLISM_OF_AMINO_ACIDS_AND_DERIVATIVES
AGXT	amino acid metabolism	REACTOME_METABOLISM_OF_AMINO_ACIDS_AND_DERIVATIVES
AGXT2	amino acid metabolism	REACTOME_METABOLISM_OF_AMINO_ACIDS_AND_DERIVATIVES
ASS1	amino acid metabolism	REACTOME_METABOLISM_OF_AMINO_ACIDS_AND_DERIVATIVES
ASL	amino acid metabolism	REACTOME_METABOLISM_OF_AMINO_ACIDS_AND_DERIVATIVES
ADSS1	amino acid metabolism	Absent
ADSS2	amino acid metabolism	Absent
ADSL	amino acid metabolism	HALLMARK_FATTY_ACID_METABOLISM
NAT8L	amino acid metabolism	REACTOME_METABOLISM_OF_AMINO_ACIDS_AND_DERIVATIVES
RIMKLB	amino acid metabolism	REACTOME_METABOLISM_OF_AMINO_ACIDS_AND_DERIVATIVES
RIMKLA	amino acid metabolism	REACTOME_METABOLISM_OF_AMINO_ACIDS_AND_DERIVATIVES
FOLH1	amino acid metabolism	REACTOME_METABOLISM_OF_AMINO_ACIDS_AND_DERIVATIVES
ASPA	amino acid metabolism	REACTOME_METABOLISM_OF_AMINO_ACIDS_AND_DERIVATIVES
GAD1	amino acid metabolism	Absent
GAD2	amino acid metabolism	HALLMARK_FATTY_ACID_METABOLISM
ABAT	amino acid metabolism	Absent
ALDH5A1	amino acid metabolism	Absent
GLUD2	amino acid metabolism	REACTOME_METABOLISM_OF_AMINO_ACIDS_AND_DERIVATIVES
GLUD1	amino acid metabolism	REACTOME_METABOLISM_OF_AMINO_ACIDS_AND_DERIVATIVES
ALDH4A1	amino acid metabolism	REACTOME_METABOLISM_OF_AMINO_ACIDS_AND_DERIVATIVES
GLUL	amino acid metabolism	HALLMARK_FATTY_ACID_METABOLISM
CAD	amino acid metabolism	Absent
GLS2	amino acid metabolism	REACTOME_METABOLISM_OF_AMINO_ACIDS_AND_DERIVATIVES
GLS	amino acid metabolism	REACTOME_METABOLISM_OF_AMINO_ACIDS_AND_DERIVATIVES
CPS1	amino acid metabolism	REACTOME_METABOLISM_OF_AMINO_ACIDS_AND_DERIVATIVES
GFPT2	amino acid metabolism	Absent
GFPT1	amino acid metabolism	Absent
PPAT	amino acid metabolism	Absent
SHMT2	amino acid metabolism	Absent
SHMT1	amino acid metabolism	REACTOME_METABOLISM_OF_AMINO_ACIDS_AND_DERIVATIVES
GRHPR	amino acid metabolism	HALLMARK_FATTY_ACID_METABOLISM
GLYCTK	amino acid metabolism	REACTOME_METABOLISM_OF_CARBOHYDRATES
PGAM1	amino acid metabolism	REACTOME_METABOLISM_OF_CARBOHYDRATES
PGAM2	amino acid metabolism	REACTOME_METABOLISM_OF_CARBOHYDRATES
PGAM4	amino acid metabolism	Absent
BPGM	amino acid metabolism	REACTOME_METABOLISM_OF_CARBOHYDRATES
PHGDH	amino acid metabolism	REACTOME_METABOLISM_OF_AMINO_ACIDS_AND_DERIVATIVES
PSAT1	amino acid metabolism	REACTOME_METABOLISM_OF_AMINO_ACIDS_AND_DERIVATIVES
PSPH	amino acid metabolism	REACTOME_METABOLISM_OF_AMINO_ACIDS_AND_DERIVATIVES
GCAT	amino acid metabolism	REACTOME_METABOLISM_OF_AMINO_ACIDS_AND_DERIVATIVES
ALAS1	amino acid metabolism	REACTOME_METABOLISM_OF_LIPIDS
ALAS2	amino acid metabolism	Absent

MAOB	amino acid metabolism	Absent
MAOA	amino acid metabolism	HALLMARK_FATTY_ACID_METABOLISM
AOC3	amino acid metabolism	HALLMARK_FATTY_ACID_METABOLISM
AOC2	amino acid metabolism	Absent
GLDC	amino acid metabolism	REACTOME_METABOLISM_OF_AMINO_ACIDS_AND_DERIVATIVES
AMT	amino acid metabolism	REACTOME_METABOLISM_OF_AMINO_ACIDS_AND_DERIVATIVES
DLD	amino acid metabolism	HALLMARK_FATTY_ACID_METABOLISM
GCSH	amino acid metabolism	REACTOME_METABOLISM_OF_AMINO_ACIDS_AND_DERIVATIVES
DAO	amino acid metabolism	REACTOME_METABOLISM_OF_AMINO_ACIDS_AND_DERIVATIVES
GATM	amino acid metabolism	REACTOME_METABOLISM_OF_AMINO_ACIDS_AND_DERIVATIVES
GAMT	amino acid metabolism	REACTOME_METABOLISM_OF_AMINO_ACIDS_AND_DERIVATIVES
CHDH	amino acid metabolism	REACTOME_METABOLISM_OF_AMINO_ACIDS_AND_DERIVATIVES
ALDH7A1	amino acid metabolism	REACTOME_METABOLISM_OF_AMINO_ACIDS_AND_DERIVATIVES
BHMT	amino acid metabolism	REACTOME_METABOLISM_OF_AMINO_ACIDS_AND_DERIVATIVES
DMGDH	amino acid metabolism	REACTOME_METABOLISM_OF_AMINO_ACIDS_AND_DERIVATIVES
PIPOX	amino acid metabolism	REACTOME_METABOLISM_OF_AMINO_ACIDS_AND_DERIVATIVES
SARDH	amino acid metabolism	REACTOME_METABOLISM_OF_AMINO_ACIDS_AND_DERIVATIVES
GNMT	amino acid metabolism	REACTOME_METABOLISM_OF_AMINO_ACIDS_AND_DERIVATIVES
CBS	amino acid metabolism	REACTOME_METABOLISM_OF_AMINO_ACIDS_AND_DERIVATIVES
CTH	amino acid metabolism	REACTOME_METABOLISM_OF_AMINO_ACIDS_AND_DERIVATIVES
SDS	amino acid metabolism	REACTOME_METABOLISM_OF_AMINO_ACIDS_AND_DERIVATIVES
SDSL	amino acid metabolism	REACTOME_METABOLISM_OF_AMINO_ACIDS_AND_DERIVATIVES
SRR	amino acid metabolism	REACTOME_METABOLISM_OF_AMINO_ACIDS_AND_DERIVATIVES
KYAT3	amino acid metabolism	REACTOME_METABOLISM_OF_AMINO_ACIDS_AND_DERIVATIVES
KYAT1	amino acid metabolism	REACTOME_METABOLISM_OF_AMINO_ACIDS_AND_DERIVATIVES
BHMT2	amino acid metabolism	REACTOME_METABOLISM_OF_AMINO_ACIDS_AND_DERIVATIVES
MTR	amino acid metabolism	REACTOME_METABOLISM_OF_AMINO_ACIDS_AND_DERIVATIVES
MAT2B	amino acid metabolism	Absent
MAT1A	amino acid metabolism	REACTOME_METABOLISM_OF_AMINO_ACIDS_AND_DERIVATIVES
MAT2A	amino acid metabolism	Absent
AMD1	amino acid metabolism	REACTOME_METABOLISM_OF_AMINO_ACIDS_AND_DERIVATIVES
SRM	amino acid metabolism	REACTOME_METABOLISM_OF_AMINO_ACIDS_AND_DERIVATIVES
SMS	amino acid metabolism	HALLMARK_FATTY_ACID_METABOLISM
MTAP	amino acid metabolism	REACTOME_METABOLISM_OF_AMINO_ACIDS_AND_DERIVATIVES
MRI1	amino acid metabolism	REACTOME_METABOLISM_OF_AMINO_ACIDS_AND_DERIVATIVES
APIP	amino acid metabolism	REACTOME_METABOLISM_OF_AMINO_ACIDS_AND_DERIVATIVES
ENOPH1	amino acid metabolism	REACTOME_METABOLISM_OF_AMINO_ACIDS_AND_DERIVATIVES
ADI1	amino acid metabolism	REACTOME_METABOLISM_OF_AMINO_ACIDS_AND_DERIVATIVES
TAT	amino acid metabolism	REACTOME_METABOLISM_OF_AMINO_ACIDS_AND_DERIVATIVES
DNMT1	amino acid metabolism	Absent
DNMT3A	amino acid metabolism	Absent
DNMT3B	amino acid metabolism	Absent
AHCYL2	amino acid metabolism	Absent
AHCYL1	amino acid metabolism	Absent
AHCY	amino acid metabolism	REACTOME_METABOLISM_OF_AMINO_ACIDS_AND_DERIVATIVES
BCAT2	amino acid metabolism	REACTOME_METABOLISM_OF_AMINO_ACIDS_AND_DERIVATIVES
BCAT1	amino acid metabolism	REACTOME_METABOLISM_OF_AMINO_ACIDS_AND_DERIVATIVES
GCLC	amino acid metabolism	Absent
GCLM	amino acid metabolism	Absent
GSS	amino acid metabolism	Absent
CDO1	amino acid metabolism	REACTOME_METABOLISM_OF_AMINO_ACIDS_AND_DERIVATIVES
MPST	amino acid metabolism	REACTOME_METABOLISM_OF_AMINO_ACIDS_AND_DERIVATIVES
TST	amino acid metabolism	REACTOME_METABOLISM_OF_AMINO_ACIDS_AND_DERIVATIVES
LDHAL6A	amino acid metabolism	Absent
LDHAL6B	amino acid metabolism	Absent
LDHA	amino acid metabolism	HALLMARK_FATTY_ACID_METABOLISM
LDHB	amino acid metabolism	Absent
LDHC	amino acid metabolism	Absent

MDH1	amino acid metabolism	HALLMARK_FATTY_ACID_METABOLISM
MDH2	amino acid metabolism	HALLMARK_FATTY_ACID_METABOLISM
AASS	amino acid metabolism	REACTOME_METABOLISM_OF_AMINO_ACIDS_AND_DERIVATIVES
AADAT	amino acid metabolism	HALLMARK_FATTY_ACID_METABOLISM
DHTKD1	amino acid metabolism	REACTOME_METABOLISM_OF_AMINO_ACIDS_AND_DERIVATIVES
DLST	amino acid metabolism	HALLMARK_FATTY_ACID_METABOLISM
GCDH	amino acid metabolism	HALLMARK_FATTY_ACID_METABOLISM
HADHA	amino acid metabolism	REACTOME_PHOSPHOLIPID_METABOLISM
EHHADH	amino acid metabolism	HALLMARK_FATTY_ACID_METABOLISM
ECHS1	amino acid metabolism	HALLMARK_FATTY_ACID_METABOLISM
HADH	amino acid metabolism	HALLMARK_FATTY_ACID_METABOLISM
ACAT2	amino acid metabolism	HALLMARK_FATTY_ACID_METABOLISM
ACAT1	amino acid metabolism	REACTOME_METABOLISM_OF_LIPIDS
HYKK	amino acid metabolism	REACTOME_METABOLISM_OF_AMINO_ACIDS_AND_DERIVATIVES
PHYKPL	amino acid metabolism	REACTOME_METABOLISM_OF_AMINO_ACIDS_AND_DERIVATIVES
CAMKMT	amino acid metabolism	Absent
KMT2A	amino acid metabolism	Absent
KMT2D	amino acid metabolism	Absent
KMT2C	amino acid metabolism	Absent
KMT2B	amino acid metabolism	Absent
KMT2E	amino acid metabolism	Absent
SETD1B	amino acid metabolism	Absent
SETD1A	amino acid metabolism	Absent
SETD7	amino acid metabolism	Absent
PRDM9	amino acid metabolism	Absent
PRDM7	amino acid metabolism	Absent
ASH1L	amino acid metabolism	Absent
SMYD2	amino acid metabolism	Absent
SMYD3	amino acid metabolism	Absent
SMYD1	amino acid metabolism	Absent
SUV39H1	amino acid metabolism	Absent
SUV39H2	amino acid metabolism	Absent
EHMT2	amino acid metabolism	Absent
EHMT1	amino acid metabolism	Absent
SETDB1	amino acid metabolism	Absent
SETDB2	amino acid metabolism	Absent
PRDM2	amino acid metabolism	Absent
EZH1	amino acid metabolism	Absent
EZH2	amino acid metabolism	Absent
SETMAR	amino acid metabolism	Absent
NSD1	amino acid metabolism	Absent
NSD2	amino acid metabolism	Absent
NSD3	amino acid metabolism	Absent
SETD2	amino acid metabolism	Absent
DOT1L	amino acid metabolism	Absent
KMT5A	amino acid metabolism	HALLMARK_FATTY_ACID_METABOLISM
PRDM6	amino acid metabolism	Absent
KMT5C	amino acid metabolism	Absent
KMT5B	amino acid metabolism	Absent
MECOM	amino acid metabolism	Absent
PRDM16	amino acid metabolism	Absent
TMLHE	amino acid metabolism	REACTOME_METABOLISM_OF_AMINO_ACIDS_AND_DERIVATIVES
ALDH2	amino acid metabolism	Absent
ALDH3A2	amino acid metabolism	HALLMARK_FATTY_ACID_METABOLISM
ALDH1B1	amino acid metabolism	Absent
ALDH9A1	amino acid metabolism	HALLMARK_FATTY_ACID_METABOLISM
BBOX1	amino acid metabolism	REACTOME_METABOLISM_OF_AMINO_ACIDS_AND_DERIVATIVES
PLOD1	amino acid metabolism	Absent

PLOD2	amino acid metabolism	Absent
PLOD3	amino acid metabolism	Absent
COLGALT1	amino acid metabolism	Absent
COLGALT2	amino acid metabolism	Absent
CKM	amino acid metabolism	REACTOME_METABOLISM_OF_AMINO_ACIDS_AND_DERIVATIVES
CKMT1A	amino acid metabolism	REACTOME_METABOLISM_OF_AMINO_ACIDS_AND_DERIVATIVES
CKMT2	amino acid metabolism	REACTOME_METABOLISM_OF_AMINO_ACIDS_AND_DERIVATIVES
CKB	amino acid metabolism	REACTOME_METABOLISM_OF_AMINO_ACIDS_AND_DERIVATIVES
CKMT1B	amino acid metabolism	REACTOME_METABOLISM_OF_AMINO_ACIDS_AND_DERIVATIVES
AZIN2	amino acid metabolism	REACTOME_METABOLISM_OF_AMINO_ACIDS_AND_DERIVATIVES
AGMAT	amino acid metabolism	REACTOME_METABOLISM_OF_AMINO_ACIDS_AND_DERIVATIVES
ODC1	amino acid metabolism	HALLMARK_FATTY_ACID_METABOLISM
AOC1	amino acid metabolism	Absent
SMOX	amino acid metabolism	REACTOME_METABOLISM_OF_AMINO_ACIDS_AND_DERIVATIVES
CNDP1	amino acid metabolism	Absent
CNDP2	amino acid metabolism	Absent
CARNS1	amino acid metabolism	REACTOME_METABOLISM_OF_AMINO_ACIDS_AND_DERIVATIVES
SAT2	amino acid metabolism	Absent
SAT1	amino acid metabolism	REACTOME_METABOLISM_OF_AMINO_ACIDS_AND_DERIVATIVES
NOS1	amino acid metabolism	Absent
NOS2	amino acid metabolism	Absent
NOS3	amino acid metabolism	Absent
ARG2	amino acid metabolism	REACTOME_METABOLISM_OF_AMINO_ACIDS_AND_DERIVATIVES
ARG1	amino acid metabolism	REACTOME_METABOLISM_OF_AMINO_ACIDS_AND_DERIVATIVES
OAT	amino acid metabolism	REACTOME_METABOLISM_OF_AMINO_ACIDS_AND_DERIVATIVES
PYCR3	amino acid metabolism	REACTOME_METABOLISM_OF_AMINO_ACIDS_AND_DERIVATIVES
PYCR2	amino acid metabolism	REACTOME_METABOLISM_OF_AMINO_ACIDS_AND_DERIVATIVES
PYCR1	amino acid metabolism	REACTOME_METABOLISM_OF_AMINO_ACIDS_AND_DERIVATIVES
PRODH	amino acid metabolism	REACTOME_METABOLISM_OF_AMINO_ACIDS_AND_DERIVATIVES
ALDH18A1	Absent	REACTOME_METABOLISM_OF_AMINO_ACIDS_AND_DERIVATIVES
LAP3	Absent	REACTOME_METABOLISM_OF_AMINO_ACIDS_AND_DERIVATIVES
P4HA2	Absent	REACTOME_METABOLISM_OF_AMINO_ACIDS_AND_DERIVATIVES
P4HA1	Absent	REACTOME_METABOLISM_OF_AMINO_ACIDS_AND_DERIVATIVES
P4HA3	Absent	REACTOME_METABOLISM_OF_AMINO_ACIDS_AND_DERIVATIVES
HOGA1	Glyoxylate and dicarboxylate metabolism	REACTOME_METABOLISM_OF_AMINO_ACIDS_AND_DERIVATIVES
L3HYPDH	Absent	REACTOME_METABOLISM_OF_AMINO_ACIDS_AND_DERIVATIVES
HAL	amino acid metabolism	REACTOME_METABOLISM_OF_AMINO_ACIDS_AND_DERIVATIVES
UROC1	amino acid metabolism	REACTOME_METABOLISM_OF_AMINO_ACIDS_AND_DERIVATIVES
AMDHD1	amino acid metabolism	REACTOME_METABOLISM_OF_AMINO_ACIDS_AND_DERIVATIVES
FTCD	amino acid metabolism	REACTOME_METABOLISM_OF_AMINO_ACIDS_AND_DERIVATIVES
HDC	amino acid metabolism	REACTOME_METABOLISM_OF_AMINO_ACIDS_AND_DERIVATIVES
HNMT	amino acid metabolism	REACTOME_METABOLISM_OF_AMINO_ACIDS_AND_DERIVATIVES
ALDH3B1	amino acid metabolism	REACTOME_METABOLISM_OF_LIPIDS
ALDH3B2	amino acid metabolism	REACTOME_METABOLISM_OF_LIPIDS
ALDH3A1	amino acid metabolism	HALLMARK_FATTY_ACID_METABOLISM
CARNMT1	amino acid metabolism	REACTOME_METABOLISM_OF_AMINO_ACIDS_AND_DERIVATIVES
PAH	amino acid metabolism	REACTOME_METABOLISM_OF_AMINO_ACIDS_AND_DERIVATIVES
DDC	amino acid metabolism	REACTOME_METABOLISM_OF_AMINO_ACIDS_AND_DERIVATIVES
HPD	amino acid metabolism	REACTOME_METABOLISM_OF_AMINO_ACIDS_AND_DERIVATIVES
MIF	amino acid metabolism	HALLMARK_FATTY_ACID_METABOLISM
TDO2	amino acid metabolism	HALLMARK_FATTY_ACID_METABOLISM
IDO1	amino acid metabolism	REACTOME_METABOLISM_OF_AMINO_ACIDS_AND_DERIVATIVES
IDO2	amino acid metabolism	REACTOME_METABOLISM_OF_AMINO_ACIDS_AND_DERIVATIVES
AFMID	amino acid metabolism	REACTOME_METABOLISM_OF_AMINO_ACIDS_AND_DERIVATIVES
KMO	amino acid metabolism	REACTOME_METABOLISM_OF_AMINO_ACIDS_AND_DERIVATIVES
KYNU	amino acid metabolism	REACTOME_METABOLISM_OF_AMINO_ACIDS_AND_DERIVATIVES
HAAO	amino acid metabolism	REACTOME_METABOLISM_OF_AMINO_ACIDS_AND_DERIVATIVES
ACMSD	amino acid metabolism	REACTOME_METABOLISM_OF_AMINO_ACIDS_AND_DERIVATIVES

ALDH8A1	amino acid metabolism	Absent
TPH2	amino acid metabolism	REACTOME_METABOLISM_OF_AMINO_ACIDS_AND_DERIVATIVES
TPH1	amino acid metabolism	REACTOME_METABOLISM_OF_AMINO_ACIDS_AND_DERIVATIVES
AOX1	amino acid metabolism	Absent
ASMT	amino acid metabolism	REACTOME_METABOLISM_OF_AMINO_ACIDS_AND_DERIVATIVES
AANAT	amino acid metabolism	REACTOME_METABOLISM_OF_AMINO_ACIDS_AND_DERIVATIVES
CYP1A1	amino acid metabolism	HALLMARK_FATTY_ACID_METABOLISM
CYP1A2	amino acid metabolism	REACTOME_METABOLISM_OF_LIPIDS
CYP1B1	amino acid metabolism	REACTOME_METABOLISM_OF_LIPIDS
INMT	amino acid metabolism	HALLMARK_FATTY_ACID_METABOLISM
CAT	amino acid metabolism	Absent
GALM	Galactose metabolism	Absent
GALK1	Amino sugar and nucleotide sugar metabolism	REACTOME_METABOLISM_OF_CARBOHYDRATES
GALT	Amino sugar and nucleotide sugar metabolism	REACTOME_METABOLISM_OF_CARBOHYDRATES
GALE	Amino sugar and nucleotide sugar metabolism	REACTOME_METABOLISM_OF_CARBOHYDRATES
UGP2	Amino sugar and nucleotide sugar metabolism	REACTOME_METABOLISM_OF_CARBOHYDRATES
PGM1	Purine metabolism	REACTOME_METABOLISM_OF_CARBOHYDRATES
PGM2	Purine metabolism	REACTOME_METABOLISM_OF_CARBOHYDRATES
HK3	Amino sugar and nucleotide sugar metabolism	REACTOME_METABOLISM_OF_CARBOHYDRATES
HK1	Amino sugar and nucleotide sugar metabolism	REACTOME_METABOLISM_OF_CARBOHYDRATES
HK2	Amino sugar and nucleotide sugar metabolism	REACTOME_METABOLISM_OF_CARBOHYDRATES
HKDC1	Amino sugar and nucleotide sugar metabolism	Absent
GCK	Amino sugar and nucleotide sugar metabolism	REACTOME_METABOLISM_OF_CARBOHYDRATES
G6PC1	Starch and sucrose metabolism	REACTOME_METABOLISM_OF_CARBOHYDRATES
G6PC2	Starch and sucrose metabolism	REACTOME_METABOLISM_OF_CARBOHYDRATES
G6PC3	Starch and sucrose metabolism	REACTOME_METABOLISM_OF_CARBOHYDRATES
GLB1	Other glycan degradation	REACTOME_METABOLISM_OF_LIPIDS
LCT	Galactose metabolism	Absent
LALBA	Galactose metabolism	REACTOME_METABOLISM_OF_CARBOHYDRATES
B4GALT1	Glycosphingolipid biosynthesis lacto and neolacto series	REACTOME_METABOLISM_OF_CARBOHYDRATES
B4GALT2	Glycosphingolipid biosynthesis lacto and neolacto series	REACTOME_METABOLISM_OF_CARBOHYDRATES
GLA	Glycosphingolipid biosynthesis globo and isoglobo series	REACTOME_METABOLISM_OF_LIPIDS
AKR1B1	Glycerolipid metabolism	REACTOME_METABOLISM_OF_LIPIDS
AKR1B10	Glycerolipid metabolism	Absent
PFKM	Galactose metabolism	REACTOME_METABOLISM_OF_CARBOHYDRATES
PFKP	Galactose metabolism	REACTOME_METABOLISM_OF_CARBOHYDRATES
PFKL	Galactose metabolism	REACTOME_METABOLISM_OF_CARBOHYDRATES
MGAM	Starch and sucrose metabolism	Absent
MGAM2	Starch and sucrose metabolism	Absent
GAA	Starch and sucrose metabolism	REACTOME_METABOLISM_OF_CARBOHYDRATES
GANC	Starch and sucrose metabolism	Absent
SI	Starch and sucrose metabolism	Absent
UGDH	Amino sugar and nucleotide sugar metabolism	HALLMARK_FATTY_ACID_METABOLISM
UGT2A1	Steroid hormone biosynthesis	Absent
UGT2A3	Steroid hormone biosynthesis	Absent
UGT2B17	Steroid hormone biosynthesis	Absent
UGT2B11	Steroid hormone biosynthesis	Absent
UGT2B28	Steroid hormone biosynthesis	Absent
UGT1A6	Steroid hormone biosynthesis	Absent

UGT1A4	Steroid hormone biosynthesis	Absent
UGT1A1	Steroid hormone biosynthesis	Absent
UGT1A3	Steroid hormone biosynthesis	Absent
UGT2B10	Steroid hormone biosynthesis	Absent
UGT1A9	Steroid hormone biosynthesis	REACTOME_METABOLISM_OF_LIPIDS
UGT2B7	Steroid hormone biosynthesis	Absent
UGT1A10	Steroid hormone biosynthesis	Absent
UGT1A8	Steroid hormone biosynthesis	Absent
UGT1A5	Steroid hormone biosynthesis	Absent
UGT2B15	Steroid hormone biosynthesis	Absent
UGT1A7	Steroid hormone biosynthesis	Absent
UGT2B4	Steroid hormone biosynthesis	Absent
UGT2A2	Steroid hormone biosynthesis	Absent
GUSB	Glycosaminoglycan degradation	REACTOME_METABOLISM_OF_CARBOHYDRATES
KL	Ascorbate and aldarate metabolism	Absent
MIOX	Inositol phosphate metabolism	Absent
AKR1A1	Glycerolipid metabolism	REACTOME_METABOLISM_OF_CARBOHYDRATES
RGN	Ascorbate and aldarate metabolism	Absent
ENPP1	Pyrimidine metabolism	Absent
ENPP3	Pyrimidine metabolism	Absent
GBA3	Starch and sucrose metabolism	Absent
GYS2	Starch and sucrose metabolism	REACTOME_METABOLISM_OF_CARBOHYDRATES
GYS1	Starch and sucrose metabolism	REACTOME_METABOLISM_OF_CARBOHYDRATES
GYG1	Starch and sucrose metabolism	REACTOME_METABOLISM_OF_CARBOHYDRATES
GYG2	Starch and sucrose metabolism	REACTOME_METABOLISM_OF_CARBOHYDRATES
GBE1	Starch and sucrose metabolism	REACTOME_METABOLISM_OF_CARBOHYDRATES
PYGL	Starch and sucrose metabolism	REACTOME_METABOLISM_OF_CARBOHYDRATES
PYGM	Starch and sucrose metabolism	REACTOME_METABOLISM_OF_CARBOHYDRATES
PYGB	Starch and sucrose metabolism	REACTOME_METABOLISM_OF_CARBOHYDRATES
AGL	Starch and sucrose metabolism	REACTOME_METABOLISM_OF_CARBOHYDRATES
AMY1C	Starch and sucrose metabolism	Absent
AMY2A	Starch and sucrose metabolism	Absent
AMY1A	Starch and sucrose metabolism	Absent
AMY2B	Starch and sucrose metabolism	Absent
AMY1B	Starch and sucrose metabolism	Absent
TREH	Starch and sucrose metabolism	Absent
PGM2L1	Starch and sucrose metabolism	REACTOME_METABOLISM_OF_CARBOHYDRATES
GPI	Amino sugar and nucleotide sugar metabolism	REACTOME_METABOLISM_OF_CARBOHYDRATES
CHIA	Amino sugar and nucleotide sugar metabolism	Absent
CHIT1	Amino sugar and nucleotide sugar metabolism	Absent
HEXA	Other glycan degradation	REACTOME_METABOLISM_OF_LIPIDS
HEXB	Other glycan degradation	REACTOME_METABOLISM_OF_LIPIDS
NAGK	Amino sugar and nucleotide sugar metabolism	Absent
AMDHD2	Amino sugar and nucleotide sugar metabolism	Absent
GNPDA1	Amino sugar and nucleotide sugar metabolism	REACTOME_METABOLISM_OF_CARBOHYDRATES
GNPDA2	Amino sugar and nucleotide sugar metabolism	REACTOME_METABOLISM_OF_CARBOHYDRATES
GNPNAT1	Amino sugar and nucleotide sugar metabolism	Absent
PGM3	Amino sugar and nucleotide sugar metabolism	Absent
GALK2	Amino sugar and nucleotide sugar metabolism	Absent
UAP1	Amino sugar and nucleotide sugar metabolism	Absent

UAP1L1	Amino sugar and nucleotide sugar metabolism	Absent
GNE	Amino sugar and nucleotide sugar metabolism	Absent
RENBP	Amino sugar and nucleotide sugar metabolism	Absent
NANS	Amino sugar and nucleotide sugar metabolism	Absent
NANP	Amino sugar and nucleotide sugar metabolism	Absent
NPL	Amino sugar and nucleotide sugar metabolism	Absent
CMAS	Amino sugar and nucleotide sugar metabolism	Absent
CYB5R1	Amino sugar and nucleotide sugar metabolism	Absent
CYB5R3	Amino sugar and nucleotide sugar metabolism	Absent
CYB5R2	Amino sugar and nucleotide sugar metabolism	Absent
CYB5RL	Amino sugar and nucleotide sugar metabolism	Absent
CYB5R4	Amino sugar and nucleotide sugar metabolism	Absent
UXS1	Amino sugar and nucleotide sugar metabolism	Absent
MPI	Amino sugar and nucleotide sugar metabolism	Absent
PMM2	Amino sugar and nucleotide sugar metabolism	Absent
PMM1	Amino sugar and nucleotide sugar metabolism	Absent
GMPPB	Amino sugar and nucleotide sugar metabolism	Absent
GMPPA	Amino sugar and nucleotide sugar metabolism	Absent
GMDS	Amino sugar and nucleotide sugar metabolism	Absent
GFUS	Amino sugar and nucleotide sugar metabolism	Absent
FCSK	Amino sugar and nucleotide sugar metabolism	Absent
FPGT	Amino sugar and nucleotide sugar metabolism	Absent
ACSS1	Propanoate metabolism	HALLMARK_FATTY_ACID_METABOLISM
ACSS2	Propanoate metabolism	Absent
PDHA2	Pyruvate metabolism	REACTOME_METABOLISM_OF_AMINO_ACIDS_AND_DERIVATIVES
PDHA1	Pyruvate metabolism	HALLMARK_FATTY_ACID_METABOLISM
PDHB	Pyruvate metabolism	HALLMARK_FATTY_ACID_METABOLISM
DLAT	Pyruvate metabolism	REACTOME_METABOLISM_OF_AMINO_ACIDS_AND_DERIVATIVES
ADH1A	Fatty acid degradation	Absent
ADH1B	Fatty acid degradation	Absent
ADH1C	Fatty acid degradation	HALLMARK_FATTY_ACID_METABOLISM
ADH7	Fatty acid degradation	HALLMARK_FATTY_ACID_METABOLISM
ADH4	Fatty acid degradation	Absent
ADH6	Fatty acid degradation	Absent
ADH5	Fatty acid degradation	Absent
PKM	Pyruvate metabolism	REACTOME_METABOLISM_OF_CARBOHYDRATES
PKLR	Pyruvate metabolism	REACTOME_METABOLISM_OF_CARBOHYDRATES
ACACA	Fatty acid biosynthesis	REACTOME_METABOLISM_OF_LIPIDS
ACACB	Fatty acid biosynthesis	REACTOME_METABOLISM_OF_LIPIDS
ACYP2	Pyruvate metabolism	Absent
ACYP1	Pyruvate metabolism	Absent
ACOT12	Pyruvate metabolism	REACTOME_METABOLISM_OF_LIPIDS
LDHD	Pyruvate metabolism	Absent
GLO1	Pyruvate metabolism	Absent

HAGH	Pyruvate metabolism	Absent
ME2	Pyruvate metabolism	Absent
ME3	Pyruvate metabolism	Absent
ME1	Pyruvate metabolism	HALLMARK_FATTY_ACID_METABOLISM
PC	Pyruvate metabolism	REACTOME_METABOLISM_OF_CARBOHYDRATES
FH	Pyruvate metabolism	HALLMARK_FATTY_ACID_METABOLISM
PCK1	Pyruvate metabolism	REACTOME_METABOLISM_OF_CARBOHYDRATES
PCK2	Pyruvate metabolism	REACTOME_METABOLISM_OF_CARBOHYDRATES
CS	Glyoxylate and dicarboxylate metabolism	Absent
ACO2	Glyoxylate and dicarboxylate metabolism	HALLMARK_FATTY_ACID_METABOLISM
ACO1	Glyoxylate and dicarboxylate metabolism	Absent
MCEE	Propanoate metabolism	HALLMARK_FATTY_ACID_METABOLISM
PCCA	Propanoate metabolism	REACTOME_METABOLISM_OF_LIPIDS
PCCB	Propanoate metabolism	REACTOME_METABOLISM_OF_LIPIDS
MMUT	Propanoate metabolism	REACTOME_METABOLISM_OF_LIPIDS
HAO2	Glyoxylate and dicarboxylate metabolism	HALLMARK_FATTY_ACID_METABOLISM
HAO1	Glyoxylate and dicarboxylate metabolism	REACTOME_METABOLISM_OF_AMINO_ACIDS_AND_DERIVATIVES
PGP	Glyoxylate and dicarboxylate metabolism	REACTOME_METABOLISM_OF_CARBOHYDRATES
HYI	Glyoxylate and dicarboxylate metabolism	Absent
ACSS3	Propanoate metabolism	REACTOME_METABOLISM_OF_LIPIDS
BCKDHA	Propanoate metabolism	REACTOME_METABOLISM_OF_AMINO_ACIDS_AND_DERIVATIVES
BCKDHB	Propanoate metabolism	HALLMARK_FATTY_ACID_METABOLISM
DBT	Propanoate metabolism	REACTOME_METABOLISM_OF_AMINO_ACIDS_AND_DERIVATIVES
ACADS	Fatty acid degradation	HALLMARK_FATTY_ACID_METABOLISM
ACOX3	Biosynthesis of unsaturated fatty acids	REACTOME_METABOLISM_OF_LIPIDS
ACOX1	Biosynthesis of unsaturated fatty acids	HALLMARK_FATTY_ACID_METABOLISM
HIBCH	Propanoate metabolism	HALLMARK_FATTY_ACID_METABOLISM
MLYCD	Propanoate metabolism	HALLMARK_FATTY_ACID_METABOLISM
ECHDC1	Propanoate metabolism	Absent
SUCLG1	Propanoate metabolism	HALLMARK_FATTY_ACID_METABOLISM
SUCLG2	Propanoate metabolism	HALLMARK_FATTY_ACID_METABOLISM
SUCLA2	Propanoate metabolism	HALLMARK_FATTY_ACID_METABOLISM
ALDH6A1	Inositol phosphate metabolism	REACTOME_METABOLISM_OF_AMINO_ACIDS_AND_DERIVATIVES
ACSM1	Butanoate metabolism	Absent
ACSM2A	Butanoate metabolism	Absent
ACSM4	Butanoate metabolism	Absent
ACSM5	Butanoate metabolism	Absent
ACSM3	Butanoate metabolism	HALLMARK_FATTY_ACID_METABOLISM
ACSM2B	Butanoate metabolism	Absent
ACSM6	Butanoate metabolism	REACTOME_METABOLISM_OF_LIPIDS
L2HGDH	Butanoate metabolism	Absent
HMGCS1	Butanoate metabolism	HALLMARK_FATTY_ACID_METABOLISM
HMGCS2	Butanoate metabolism	HALLMARK_FATTY_ACID_METABOLISM
HMGCL	Butanoate metabolism	HALLMARK_FATTY_ACID_METABOLISM
HMGCLL1	Butanoate metabolism	REACTOME_METABOLISM_OF_LIPIDS
OXCT1	Butanoate metabolism	REACTOME_METABOLISM_OF_LIPIDS
OXCT2	Butanoate metabolism	REACTOME_METABOLISM_OF_LIPIDS
AACS	Butanoate metabolism	REACTOME_METABOLISM_OF_LIPIDS
BDH1	Butanoate metabolism	REACTOME_METABOLISM_OF_LIPIDS
BDH2	Butanoate metabolism	REACTOME_METABOLISM_OF_LIPIDS
PIK3C3	Inositol phosphate metabolism	REACTOME_PHOSPHOLIPID_METABOLISM
MTM1	Inositol phosphate metabolism	REACTOME_PHOSPHOLIPID_METABOLISM
MTMR1	Inositol phosphate metabolism	REACTOME_PHOSPHOLIPID_METABOLISM
MTMR2	Inositol phosphate metabolism	REACTOME_PHOSPHOLIPID_METABOLISM
MTMR3	Inositol phosphate metabolism	REACTOME_PHOSPHOLIPID_METABOLISM
MTMR4	Inositol phosphate metabolism	REACTOME_PHOSPHOLIPID_METABOLISM
MTMR8	Inositol phosphate metabolism	REACTOME_PHOSPHOLIPID_METABOLISM
MTMR6	Inositol phosphate metabolism	REACTOME_PHOSPHOLIPID_METABOLISM

MTMR7	Inositol phosphate metabolism	REACTOME_PHOSPHOLIPID_METABOLISM
MTMR14	Inositol phosphate metabolism	REACTOME_PHOSPHOLIPID_METABOLISM
PI4KA	Inositol phosphate metabolism	REACTOME_PHOSPHOLIPID_METABOLISM
PI4KB	Inositol phosphate metabolism	REACTOME_PHOSPHOLIPID_METABOLISM
PI4K2A	Inositol phosphate metabolism	REACTOME_PHOSPHOLIPID_METABOLISM
PI4K2B	Inositol phosphate metabolism	REACTOME_PHOSPHOLIPID_METABOLISM
SACM1L	Inositol phosphate metabolism	REACTOME_PHOSPHOLIPID_METABOLISM
INPP5F	Inositol phosphate metabolism	REACTOME_PHOSPHOLIPID_METABOLISM
PIP5K1C	Inositol phosphate metabolism	REACTOME_PHOSPHOLIPID_METABOLISM
PIP5K1A	Inositol phosphate metabolism	REACTOME_PHOSPHOLIPID_METABOLISM
PIP5K1B	Inositol phosphate metabolism	REACTOME_PHOSPHOLIPID_METABOLISM
PIP5KL1	Inositol phosphate metabolism	Absent
OCRL	Inositol phosphate metabolism	REACTOME_PHOSPHOLIPID_METABOLISM
INPP5B	Inositol phosphate metabolism	Absent
INPP5E	Inositol phosphate metabolism	REACTOME_PHOSPHOLIPID_METABOLISM
SYNJ1	Inositol phosphate metabolism	REACTOME_PHOSPHOLIPID_METABOLISM
SYNJ2	Inositol phosphate metabolism	REACTOME_PHOSPHOLIPID_METABOLISM
PIK3CA	Inositol phosphate metabolism	REACTOME_PHOSPHOLIPID_METABOLISM
PIK3CD	Inositol phosphate metabolism	REACTOME_PHOSPHOLIPID_METABOLISM
PIK3CB	Inositol phosphate metabolism	REACTOME_PHOSPHOLIPID_METABOLISM
PIK3CG	Inositol phosphate metabolism	REACTOME_PHOSPHOLIPID_METABOLISM
PTEN	Inositol phosphate metabolism	REACTOME_PHOSPHOLIPID_METABOLISM
INPP5D	Inositol phosphate metabolism	REACTOME_PHOSPHOLIPID_METABOLISM
INPPL1	Inositol phosphate metabolism	REACTOME_PHOSPHOLIPID_METABOLISM
PIK3C2G	Inositol phosphate metabolism	REACTOME_PHOSPHOLIPID_METABOLISM
PIK3C2A	Inositol phosphate metabolism	REACTOME_PHOSPHOLIPID_METABOLISM
PIK3C2B	Inositol phosphate metabolism	REACTOME_PHOSPHOLIPID_METABOLISM
INPP4A	Inositol phosphate metabolism	REACTOME_PHOSPHOLIPID_METABOLISM
INPP4B	Inositol phosphate metabolism	REACTOME_PHOSPHOLIPID_METABOLISM
PIKFYVE	Inositol phosphate metabolism	REACTOME_PHOSPHOLIPID_METABOLISM
FIG4	Inositol phosphate metabolism	REACTOME_PHOSPHOLIPID_METABOLISM
PIP4K2C	Inositol phosphate metabolism	REACTOME_PHOSPHOLIPID_METABOLISM
PIP4K2A	Inositol phosphate metabolism	REACTOME_PHOSPHOLIPID_METABOLISM
PIP4K2B	Inositol phosphate metabolism	REACTOME_PHOSPHOLIPID_METABOLISM
PLCB1	Inositol phosphate metabolism	Absent
PLCB2	Inositol phosphate metabolism	Absent
PLCB3	Inositol phosphate metabolism	Absent
PLCB4	Inositol phosphate metabolism	Absent
PLCD1	Inositol phosphate metabolism	Absent
PLCD3	Inositol phosphate metabolism	Absent
PLCD4	Inositol phosphate metabolism	Absent
PLCE1	Inositol phosphate metabolism	Absent
PLCG1	Inositol phosphate metabolism	Absent
PLCG2	Inositol phosphate metabolism	Absent
PLCZ1	Inositol phosphate metabolism	Absent
PLCH1	Inositol phosphate metabolism	Absent
PLCH2	Inositol phosphate metabolism	Absent
IMPA2	Inositol phosphate metabolism	Absent
IMPA1	Inositol phosphate metabolism	Absent
CDIPT	Glycerophospholipid metabolism	REACTOME_PHOSPHOLIPID_METABOLISM
ISYNA1	Inositol phosphate metabolism	Absent
INPP1	Inositol phosphate metabolism	Absent
INPP5A	Inositol phosphate metabolism	Absent
INPP5K	Inositol phosphate metabolism	REACTOME_PHOSPHOLIPID_METABOLISM
INPP5J	Inositol phosphate metabolism	REACTOME_PHOSPHOLIPID_METABOLISM
MINPP1	Inositol phosphate metabolism	Absent
ITPKB	Inositol phosphate metabolism	Absent
ITPKA	Inositol phosphate metabolism	Absent

ITPKC	Inositol phosphate metabolism	Absent
ITPK1	Inositol phosphate metabolism	Absent
IPMK	Inositol phosphate metabolism	Absent
IPPK	Inositol phosphate metabolism	Absent
TPI1	Inositol phosphate metabolism	REACTOME_METABOLISM_OF_CARBOHYDRATES
NDUFS1	Oxidative phosphorylation	Absent
NDUFS2	Oxidative phosphorylation	Absent
NDUFS3	Oxidative phosphorylation	Absent
NDUFS4	Oxidative phosphorylation	Absent
NDUFS5	Oxidative phosphorylation	Absent
NDUFS6	Oxidative phosphorylation	Absent
NDUFS7	Oxidative phosphorylation	Absent
NDUFS8	Oxidative phosphorylation	Absent
NDUFV1	Oxidative phosphorylation	Absent
NDUFV2	Oxidative phosphorylation	Absent
NDUFV3	Oxidative phosphorylation	Absent
NDUFA1	Oxidative phosphorylation	Absent
NDUFA2	Oxidative phosphorylation	Absent
NDUFA3	Oxidative phosphorylation	Absent
NDUFA4	Oxidative phosphorylation	Absent
NDUFA4L2	Oxidative phosphorylation	Absent
NDUFA5	Oxidative phosphorylation	Absent
NDUFA6	Oxidative phosphorylation	Absent
NDUFA7	Oxidative phosphorylation	Absent
NDUFA8	Oxidative phosphorylation	Absent
NDUFA9	Oxidative phosphorylation	Absent
NDUFA10	Oxidative phosphorylation	Absent
NDUFAB1	Oxidative phosphorylation	REACTOME_METABOLISM_OF_LIPIDS
NDUFA11	Oxidative phosphorylation	Absent
NDUFA12	Oxidative phosphorylation	Absent
NDUFA13	Oxidative phosphorylation	Absent
NDUFB1	Oxidative phosphorylation	Absent
NDUFB2	Oxidative phosphorylation	Absent
NDUFB3	Oxidative phosphorylation	Absent
NDUFB4	Oxidative phosphorylation	Absent
NDUFB5	Oxidative phosphorylation	Absent
NDUFB6	Oxidative phosphorylation	Absent
NDUFB7	Oxidative phosphorylation	Absent
NDUFB8	Oxidative phosphorylation	Absent
NDUFB9	Oxidative phosphorylation	Absent
NDUFB10	Oxidative phosphorylation	Absent
NDUFB11	Oxidative phosphorylation	Absent
NDUFC1	Oxidative phosphorylation	Absent
NDUFC2	Oxidative phosphorylation	Absent
SDHA	Oxidative phosphorylation	HALLMARK_FATTY_ACID_METABOLISM
SDHB	Oxidative phosphorylation	Absent
SDHC	Oxidative phosphorylation	HALLMARK_FATTY_ACID_METABOLISM
SDHD	Oxidative phosphorylation	HALLMARK_FATTY_ACID_METABOLISM
UQCRFS1	Oxidative phosphorylation	Absent
CYC1	Oxidative phosphorylation	Absent
UQCRC1	Oxidative phosphorylation	Absent
UQCRC2	Oxidative phosphorylation	Absent
UQCRH	Oxidative phosphorylation	Absent
UQCRHL	Oxidative phosphorylation	Absent
UQCRB	Oxidative phosphorylation	Absent
UQCRQ	Oxidative phosphorylation	Absent
UQCR10	Oxidative phosphorylation	Absent
UQCR11	Oxidative phosphorylation	Absent

COX10	Oxidative phosphorylation	Absent
COX4I2	Oxidative phosphorylation	Absent
COX4I1	Oxidative phosphorylation	Absent
COX5A	Oxidative phosphorylation	Absent
COX5B	Oxidative phosphorylation	Absent
COX6A1	Oxidative phosphorylation	Absent
COX6A2	Oxidative phosphorylation	Absent
COX6B1	Oxidative phosphorylation	Absent
COX6B2	Oxidative phosphorylation	Absent
COX6C	Oxidative phosphorylation	Absent
COX7A1	Oxidative phosphorylation	Absent
COX7A2	Oxidative phosphorylation	Absent
COX7A2L	Oxidative phosphorylation	Absent
COX7B	Oxidative phosphorylation	Absent
COX7B2	Oxidative phosphorylation	Absent
COX7C	Oxidative phosphorylation	Absent
COX8C	Oxidative phosphorylation	Absent
COX8A	Oxidative phosphorylation	Absent
COX11	Oxidative phosphorylation	Absent
COX15	Oxidative phosphorylation	Absent
COX17	Oxidative phosphorylation	Absent
CYCS	Oxidative phosphorylation	Absent
ATP5F1A	Oxidative phosphorylation	Absent
ATP5F1B	Oxidative phosphorylation	Absent
ATP5F1C	Oxidative phosphorylation	Absent
ATP5F1D	Oxidative phosphorylation	Absent
ATP5F1E	Oxidative phosphorylation	Absent
ATP5PO	Oxidative phosphorylation	Absent
ATP5PB	Oxidative phosphorylation	Absent
ATP5MC1	Oxidative phosphorylation	Absent
ATP5MC2	Oxidative phosphorylation	Absent
ATP5MC3	Oxidative phosphorylation	Absent
ATP5PD	Oxidative phosphorylation	Absent
ATP5ME	Oxidative phosphorylation	Absent
ATP5MF	Oxidative phosphorylation	Absent
ATP5MG	Oxidative phosphorylation	Absent
ATP5PF	Oxidative phosphorylation	Absent
ATP6V1A	Oxidative phosphorylation	Absent
ATP6V1B1	Oxidative phosphorylation	Absent
ATP6V1B2	Oxidative phosphorylation	Absent
ATP6V1C2	Oxidative phosphorylation	Absent
ATP6V1C1	Oxidative phosphorylation	Absent
ATP6V1D	Oxidative phosphorylation	Absent
ATP6V1E2	Oxidative phosphorylation	Absent
ATP6V1E1	Oxidative phosphorylation	Absent
ATP6V1F	Oxidative phosphorylation	Absent
ATP6V1G1	Oxidative phosphorylation	Absent
ATP6V1G3	Oxidative phosphorylation	Absent
ATP6V1G2	Oxidative phosphorylation	Absent
ATP6V1H	Oxidative phosphorylation	Absent
TCIRG1	Oxidative phosphorylation	Absent
ATP6V0A2	Oxidative phosphorylation	Absent
ATP6V0A4	Oxidative phosphorylation	Absent
ATP6V0A1	Oxidative phosphorylation	Absent
ATP6V0C	Oxidative phosphorylation	Absent
ATP6V0B	Oxidative phosphorylation	Absent
ATP6V0D1	Oxidative phosphorylation	Absent
ATP6V0D2	Oxidative phosphorylation	Absent

ATP6V0E1	Oxidative phosphorylation	Absent
ATP6V0E2	Oxidative phosphorylation	Absent
ATP6AP1	Oxidative phosphorylation	Absent
ATP4A	Oxidative phosphorylation	Absent
ATP4B	Oxidative phosphorylation	Absent
ATP12A	Oxidative phosphorylation	Absent
PPA2	Oxidative phosphorylation	Absent
PPA1	Oxidative phosphorylation	Absent
LHPP	Oxidative phosphorylation	Absent
CA13	Nitrogen metabolism	Absent
CA1	Nitrogen metabolism	Absent
CA6	Nitrogen metabolism	HALLMARK_FATTY_ACID_METABOLISM
CA7	Nitrogen metabolism	Absent
CA12	Nitrogen metabolism	Absent
CA5B	Nitrogen metabolism	Absent
CA14	Nitrogen metabolism	Absent
CA9	Nitrogen metabolism	Absent
CA3	Nitrogen metabolism	Absent
CA5A	Nitrogen metabolism	Absent
CA8	Nitrogen metabolism	Absent
CA2	Nitrogen metabolism	HALLMARK_FATTY_ACID_METABOLISM
CA4	Nitrogen metabolism	HALLMARK_FATTY_ACID_METABOLISM
PAPSS2	Purine metabolism	REACTOME_METABOLISM_OF_CARBOHYDRATES
PAPSS1	Purine metabolism	REACTOME_METABOLISM_OF_CARBOHYDRATES
BPNT1	Sulfur metabolism	Absent
BPNT2	Sulfur metabolism	Absent
SUOX	Sulfur metabolism	REACTOME_METABOLISM_OF_AMINO_ACIDS_AND_DERIVATIVES
SQOR	Sulfur metabolism	REACTOME_METABOLISM_OF_AMINO_ACIDS_AND_DERIVATIVES
ETHE1	Sulfur metabolism	REACTOME_METABOLISM_OF_AMINO_ACIDS_AND_DERIVATIVES
SELENBP1	Sulfur metabolism	Absent
ACSF3	Fatty acid biosynthesis	REACTOME_METABOLISM_OF_LIPIDS
MCAT	Fatty acid biosynthesis	REACTOME_METABOLISM_OF_LIPIDS
FASN	Fatty acid biosynthesis	HALLMARK_FATTY_ACID_METABOLISM
OXSM	Fatty acid biosynthesis	Absent
CBR4	Fatty acid biosynthesis	REACTOME_METABOLISM_OF_LIPIDS
MECR	Fatty acid elongation	REACTOME_METABOLISM_OF_LIPIDS
OLAH	Fatty acid biosynthesis	REACTOME_METABOLISM_OF_LIPIDS
ACSL6	Fatty acid degradation	REACTOME_METABOLISM_OF_LIPIDS
ACSL4	Fatty acid degradation	HALLMARK_FATTY_ACID_METABOLISM
ACSL1	Fatty acid degradation	HALLMARK_FATTY_ACID_METABOLISM
ACSL5	Fatty acid degradation	HALLMARK_FATTY_ACID_METABOLISM
ACSL3	Fatty acid degradation	REACTOME_METABOLISM_OF_LIPIDS
ACSBG1	Fatty acid degradation	REACTOME_METABOLISM_OF_LIPIDS
ACSBG2	Fatty acid degradation	REACTOME_METABOLISM_OF_LIPIDS
ACAA2	Fatty acid degradation	HALLMARK_FATTY_ACID_METABOLISM
HADHB	Fatty acid degradation	HALLMARK_FATTY_ACID_METABOLISM
PPT1	Fatty acid elongation	REACTOME_METABOLISM_OF_LIPIDS
PPT2	Fatty acid elongation	REACTOME_METABOLISM_OF_LIPIDS
ELOVL1	Biosynthesis of unsaturated fatty acids	REACTOME_METABOLISM_OF_LIPIDS
ELOVL2	Biosynthesis of unsaturated fatty acids	REACTOME_METABOLISM_OF_LIPIDS
ELOVL3	Biosynthesis of unsaturated fatty acids	REACTOME_METABOLISM_OF_LIPIDS
ELOVL4	Biosynthesis of unsaturated fatty acids	REACTOME_METABOLISM_OF_LIPIDS
ELOVL5	Biosynthesis of unsaturated fatty acids	HALLMARK_FATTY_ACID_METABOLISM
ELOVL6	Biosynthesis of unsaturated fatty acids	REACTOME_METABOLISM_OF_LIPIDS
ELOVL7	Biosynthesis of unsaturated fatty acids	REACTOME_METABOLISM_OF_LIPIDS
HSD17B12	Biosynthesis of unsaturated fatty acids	REACTOME_METABOLISM_OF_LIPIDS
HACD2	Biosynthesis of unsaturated fatty acids	REACTOME_METABOLISM_OF_LIPIDS
HACD1	Biosynthesis of unsaturated fatty acids	REACTOME_METABOLISM_OF_LIPIDS

HACD4	Biosynthesis of unsaturated fatty acids	REACTOME_METABOLISM_OF_LIPIDS
HACD3	Biosynthesis of unsaturated fatty acids	REACTOME_METABOLISM_OF_LIPIDS
TECR	Biosynthesis of unsaturated fatty acids	REACTOME_METABOLISM_OF_LIPIDS
ACOT4	Biosynthesis of unsaturated fatty acids	REACTOME_METABOLISM_OF_LIPIDS
ACOT2	Biosynthesis of unsaturated fatty acids	HALLMARK_FATTY_ACID_METABOLISM
ACOT1	Biosynthesis of unsaturated fatty acids	REACTOME_METABOLISM_OF_LIPIDS
ACOT7	Biosynthesis of unsaturated fatty acids	REACTOME_METABOLISM_OF_LIPIDS
THEM4	Fatty acid elongation	REACTOME_METABOLISM_OF_LIPIDS
THEM5	Fatty acid elongation	REACTOME_METABOLISM_OF_LIPIDS
ACAA1	Biosynthesis of unsaturated fatty acids	HALLMARK_FATTY_ACID_METABOLISM
ACADM	Fatty acid degradation	HALLMARK_FATTY_ACID_METABOLISM
ACADL	Fatty acid degradation	HALLMARK_FATTY_ACID_METABOLISM
ACADSB	Fatty acid degradation	REACTOME_METABOLISM_OF_AMINO_ACIDS_AND_DERIVATIVES
ACADVL	Fatty acid degradation	HALLMARK_FATTY_ACID_METABOLISM
CPT1A	Fatty acid degradation	HALLMARK_FATTY_ACID_METABOLISM
CPT1B	Fatty acid degradation	REACTOME_METABOLISM_OF_LIPIDS
CPT1C	Fatty acid degradation	Absent
CPT2	Fatty acid degradation	HALLMARK_FATTY_ACID_METABOLISM
ECI1	Fatty acid degradation	HALLMARK_FATTY_ACID_METABOLISM
ECI2	Fatty acid degradation	HALLMARK_FATTY_ACID_METABOLISM
CYP2U1	Arachidonic acid metabolism	REACTOME_METABOLISM_OF_LIPIDS
FDFT1	Steroid biosynthesis	REACTOME_METABOLISM_OF_LIPIDS
SQLE	Steroid biosynthesis	REACTOME_METABOLISM_OF_LIPIDS
LSS	Steroid biosynthesis	REACTOME_METABOLISM_OF_LIPIDS
CYP51A1	Steroid biosynthesis	REACTOME_METABOLISM_OF_LIPIDS
TM7SF2	Steroid biosynthesis	REACTOME_METABOLISM_OF_LIPIDS
LBR	Steroid biosynthesis	REACTOME_METABOLISM_OF_LIPIDS
MSMO1	Steroid biosynthesis	REACTOME_METABOLISM_OF_LIPIDS
NSDHL	Steroid biosynthesis	HALLMARK_FATTY_ACID_METABOLISM
HSD17B7	Steroid hormone biosynthesis	HALLMARK_FATTY_ACID_METABOLISM
EBP	Steroid biosynthesis	REACTOME_METABOLISM_OF_LIPIDS
DHCR24	Steroid biosynthesis	HALLMARK_FATTY_ACID_METABOLISM
SC5D	Steroid biosynthesis	REACTOME_METABOLISM_OF_LIPIDS
DHCR7	Steroid biosynthesis	REACTOME_METABOLISM_OF_LIPIDS
LIPA	Steroid biosynthesis	Absent
CEL	Glycerolipid metabolism	HALLMARK_FATTY_ACID_METABOLISM
SOAT2	Steroid biosynthesis	Absent
SOAT1	Steroid biosynthesis	Absent
CYP2R1	Steroid biosynthesis	REACTOME_METABOLISM_OF_LIPIDS
CYP27B1	Steroid biosynthesis	REACTOME_METABOLISM_OF_LIPIDS
CYP24A1	Steroid biosynthesis	REACTOME_METABOLISM_OF_LIPIDS
CYP46A1	Primary bile acid biosynthesis	REACTOME_METABOLISM_OF_LIPIDS
CYP39A1	Primary bile acid biosynthesis	REACTOME_METABOLISM_OF_LIPIDS
HSD3B7	Primary bile acid biosynthesis	REACTOME_METABOLISM_OF_LIPIDS
CH25H	Primary bile acid biosynthesis	REACTOME_METABOLISM_OF_LIPIDS
CYP7B1	Steroid hormone biosynthesis	REACTOME_METABOLISM_OF_LIPIDS
CYP7A1	Steroid hormone biosynthesis	REACTOME_METABOLISM_OF_LIPIDS
CYP27A1	Primary bile acid biosynthesis	REACTOME_METABOLISM_OF_LIPIDS
CYP8B1	Primary bile acid biosynthesis	REACTOME_METABOLISM_OF_LIPIDS
AKR1D1	Steroid hormone biosynthesis	REACTOME_METABOLISM_OF_LIPIDS
AKR1C4	Steroid hormone biosynthesis	REACTOME_METABOLISM_OF_LIPIDS
SLC27A5	Primary bile acid biosynthesis	REACTOME_METABOLISM_OF_LIPIDS
AMACR	Primary bile acid biosynthesis	REACTOME_METABOLISM_OF_LIPIDS
ACOX2	Primary bile acid biosynthesis	REACTOME_METABOLISM_OF_LIPIDS
HSD17B4	Biosynthesis of unsaturated fatty acids	HALLMARK_FATTY_ACID_METABOLISM
SCP2	Biosynthesis of unsaturated fatty acids	REACTOME_METABOLISM_OF_LIPIDS
ACOT8	Primary bile acid biosynthesis	HALLMARK_FATTY_ACID_METABOLISM
BAAT	Biosynthesis of unsaturated fatty acids	REACTOME_METABOLISM_OF_LIPIDS

CYP11A1	Steroid hormone biosynthesis	REACTOME_METABOLISM_OF_LIPIDS
CYP17A1	Steroid hormone biosynthesis	REACTOME_METABOLISM_OF_LIPIDS
STS	Steroid hormone biosynthesis	REACTOME_METABOLISM_OF_LIPIDS
SULT2B1	Steroid hormone biosynthesis	Absent
CYP21A2	Steroid hormone biosynthesis	REACTOME_METABOLISM_OF_LIPIDS
HSD3B1	Steroid hormone biosynthesis	REACTOME_METABOLISM_OF_LIPIDS
HSD3B2	Steroid hormone biosynthesis	REACTOME_METABOLISM_OF_LIPIDS
SRD5A1	Steroid hormone biosynthesis	REACTOME_METABOLISM_OF_LIPIDS
SRD5A2	Steroid hormone biosynthesis	REACTOME_METABOLISM_OF_LIPIDS
SRD5A3	N Glycan biosynthesis	REACTOME_METABOLISM_OF_LIPIDS
AKR1C2	Steroid hormone biosynthesis	REACTOME_METABOLISM_OF_LIPIDS
AKR1C3	Arachidonic acid metabolism	REACTOME_METABOLISM_OF_LIPIDS
CYP11B1	Steroid hormone biosynthesis	REACTOME_METABOLISM_OF_LIPIDS
CYP11B2	Steroid hormone biosynthesis	REACTOME_METABOLISM_OF_LIPIDS
HSD11B1	Steroid hormone biosynthesis	REACTOME_METABOLISM_OF_LIPIDS
HSD11B1L	Steroid hormone biosynthesis	Absent
HSD11B2	Steroid hormone biosynthesis	REACTOME_METABOLISM_OF_LIPIDS
AKR1C1	Steroid hormone biosynthesis	REACTOME_METABOLISM_OF_LIPIDS
SULT1E1	Steroid hormone biosynthesis	Absent
HSD17B1	Steroid hormone biosynthesis	REACTOME_METABOLISM_OF_LIPIDS
HSD17B2	Steroid hormone biosynthesis	REACTOME_METABOLISM_OF_LIPIDS
HSD17B6	Steroid hormone biosynthesis	Absent
DHRS11	Steroid hormone biosynthesis	Absent
CYP3A5	Steroid hormone biosynthesis	Absent
CYP3A7	Steroid hormone biosynthesis	Absent
CYP2E1	Linoleic acid metabolism	REACTOME_METABOLISM_OF_LIPIDS
CYP3A4	Linoleic acid metabolism	REACTOME_METABOLISM_OF_LIPIDS
CYP19A1	Steroid hormone biosynthesis	REACTOME_METABOLISM_OF_LIPIDS
COMT	Steroid hormone biosynthesis	Absent
LRTOMT	Steroid hormone biosynthesis	Absent
HSD17B3	Steroid hormone biosynthesis	REACTOME_METABOLISM_OF_LIPIDS
TKFC	Glycerolipid metabolism	REACTOME_METABOLISM_OF_CARBOHYDRATES
GK2	Glycerolipid metabolism	REACTOME_METABOLISM_OF_LIPIDS
GK	Glycerolipid metabolism	REACTOME_METABOLISM_OF_LIPIDS
GPAM	Glycerophospholipid metabolism	REACTOME_PHOSPHOLIPID_METABOLISM
GPAT2	Glycerophospholipid metabolism	REACTOME_PHOSPHOLIPID_METABOLISM
GPAT4	Glycerophospholipid metabolism	REACTOME_PHOSPHOLIPID_METABOLISM
GPAT3	Glycerophospholipid metabolism	REACTOME_PHOSPHOLIPID_METABOLISM
AGPAT1	Glycerophospholipid metabolism	REACTOME_PHOSPHOLIPID_METABOLISM
AGPAT2	Glycerophospholipid metabolism	REACTOME_PHOSPHOLIPID_METABOLISM
AGPAT3	Glycerophospholipid metabolism	REACTOME_PHOSPHOLIPID_METABOLISM
AGPAT4	Glycerophospholipid metabolism	REACTOME_PHOSPHOLIPID_METABOLISM
AGPAT5	Glycerophospholipid metabolism	REACTOME_PHOSPHOLIPID_METABOLISM
LCLAT1	Glycerophospholipid metabolism	REACTOME_PHOSPHOLIPID_METABOLISM
MBOAT1	Glycerophospholipid metabolism	REACTOME_PHOSPHOLIPID_METABOLISM
MBOAT2	Glycerophospholipid metabolism	REACTOME_PHOSPHOLIPID_METABOLISM
PLPP1	Sphingolipid metabolism	REACTOME_METABOLISM_OF_LIPIDS
PLPP3	Sphingolipid metabolism	REACTOME_METABOLISM_OF_LIPIDS
PLPP2	Sphingolipid metabolism	REACTOME_METABOLISM_OF_LIPIDS
LPIN1	Glycerophospholipid metabolism	REACTOME_PHOSPHOLIPID_METABOLISM
LPIN3	Glycerophospholipid metabolism	REACTOME_PHOSPHOLIPID_METABOLISM
LPIN2	Glycerophospholipid metabolism	REACTOME_PHOSPHOLIPID_METABOLISM
PLPP5	Glycerophospholipid metabolism	Absent
PLPP4	Glycerophospholipid metabolism	Absent
DGKZ	Glycerophospholipid metabolism	Absent
DGKD	Glycerophospholipid metabolism	Absent
DGKI	Glycerophospholipid metabolism	Absent
DGKA	Glycerophospholipid metabolism	Absent

DGKE	Glycerophospholipid metabolism	Absent
DGKB	Glycerophospholipid metabolism	Absent
DGKH	Glycerophospholipid metabolism	Absent
DGKG	Glycerophospholipid metabolism	Absent
DGKQ	Glycerophospholipid metabolism	Absent
DGKK	Glycerophospholipid metabolism	Absent
DGAT1	Glycerolipid metabolism	REACTOME_PHOSPHOLIPID_METABOLISM
DGAT2	Glycerolipid metabolism	REACTOME_PHOSPHOLIPID_METABOLISM
MOGAT3	Glycerolipid metabolism	REACTOME_METABOLISM_OF_LIPIDS
PNPLA2	Glycerolipid metabolism	REACTOME_PHOSPHOLIPID_METABOLISM
PNPLA3	Glycerolipid metabolism	REACTOME_PHOSPHOLIPID_METABOLISM
PNLIP	Glycerolipid metabolism	Absent
PNLIPRP1	Glycerolipid metabolism	Absent
PNLIPRP2	Glycerolipid metabolism	Absent
PNLIPRP3	Glycerolipid metabolism	Absent
LIPC	Glycerolipid metabolism	Absent
LIPF	Glycerolipid metabolism	Absent
LIPG	Glycerolipid metabolism	Absent
LPL	Glycerolipid metabolism	Absent
AGK	Glycerolipid metabolism	REACTOME_PHOSPHOLIPID_METABOLISM
MGLL	Glycerolipid metabolism	HALLMARK_FATTY_ACID_METABOLISM
MOGAT1	Glycerolipid metabolism	REACTOME_METABOLISM_OF_LIPIDS
MOGAT2	Glycerolipid metabolism	REACTOME_METABOLISM_OF_LIPIDS
GPD1L	Glycerophospholipid metabolism	REACTOME_PHOSPHOLIPID_METABOLISM
GPD1	Glycerophospholipid metabolism	HALLMARK_FATTY_ACID_METABOLISM
GPD2	Glycerophospholipid metabolism	HALLMARK_FATTY_ACID_METABOLISM
GNPAT	Glycerophospholipid metabolism	REACTOME_PHOSPHOLIPID_METABOLISM
ADPRM	Purine metabolism	Absent
CHPT1	Ether lipid metabolism	REACTOME_PHOSPHOLIPID_METABOLISM
CEPT1	Ether lipid metabolism	REACTOME_PHOSPHOLIPID_METABOLISM
PLD1	Ether lipid metabolism	REACTOME_PHOSPHOLIPID_METABOLISM
PLD2	Ether lipid metabolism	REACTOME_PHOSPHOLIPID_METABOLISM
PLD3	Ether lipid metabolism	REACTOME_PHOSPHOLIPID_METABOLISM
PLD4	Ether lipid metabolism	REACTOME_PHOSPHOLIPID_METABOLISM
LCAT	Glycerophospholipid metabolism	Absent
PLA2G10	alpha Linolenic acid metabolism	REACTOME_PHOSPHOLIPID_METABOLISM
PLA2G2D	alpha Linolenic acid metabolism	REACTOME_PHOSPHOLIPID_METABOLISM
PLA2G2E	alpha Linolenic acid metabolism	REACTOME_PHOSPHOLIPID_METABOLISM
PLA2G3	alpha Linolenic acid metabolism	REACTOME_PHOSPHOLIPID_METABOLISM
PLA2G2F	alpha Linolenic acid metabolism	REACTOME_PHOSPHOLIPID_METABOLISM
PLA2G12A	alpha Linolenic acid metabolism	REACTOME_PHOSPHOLIPID_METABOLISM
PLA2G12B	alpha Linolenic acid metabolism	Absent
PLA2G1B	alpha Linolenic acid metabolism	REACTOME_PHOSPHOLIPID_METABOLISM
PLA2G5	alpha Linolenic acid metabolism	REACTOME_PHOSPHOLIPID_METABOLISM
PLA2G2A	alpha Linolenic acid metabolism	REACTOME_PHOSPHOLIPID_METABOLISM
PLA2G2C	alpha Linolenic acid metabolism	Absent
PLA2G4E	alpha Linolenic acid metabolism	REACTOME_PHOSPHOLIPID_METABOLISM
PLA2G4A	alpha Linolenic acid metabolism	REACTOME_PHOSPHOLIPID_METABOLISM
MJD7-PLA2G4I	alpha Linolenic acid metabolism	Absent
PLA2G4C	alpha Linolenic acid metabolism	REACTOME_PHOSPHOLIPID_METABOLISM
PLA2G4D	alpha Linolenic acid metabolism	REACTOME_PHOSPHOLIPID_METABOLISM
PLA2G4F	alpha Linolenic acid metabolism	REACTOME_PHOSPHOLIPID_METABOLISM
PLA2G6	alpha Linolenic acid metabolism	REACTOME_PHOSPHOLIPID_METABOLISM
PLB1	alpha Linolenic acid metabolism	REACTOME_PHOSPHOLIPID_METABOLISM
PLAAT3	alpha Linolenic acid metabolism	REACTOME_PHOSPHOLIPID_METABOLISM
LPCAT2	Ether lipid metabolism	REACTOME_PHOSPHOLIPID_METABOLISM
LPCAT1	Ether lipid metabolism	REACTOME_PHOSPHOLIPID_METABOLISM
LPCAT4	Ether lipid metabolism	REACTOME_PHOSPHOLIPID_METABOLISM

LPCAT3	Glycerophospholipid metabolism	REACTOME_PHOSPHOLIPID_METABOLISM
LYPLA1	Glycerophospholipid metabolism	Absent
PLA2G15	Glycerophospholipid metabolism	REACTOME_PHOSPHOLIPID_METABOLISM
LYPLA2	Glycerophospholipid metabolism	Absent
PNPLA6	Glycerophospholipid metabolism	REACTOME_PHOSPHOLIPID_METABOLISM
PNPLA7	Glycerophospholipid metabolism	REACTOME_PHOSPHOLIPID_METABOLISM
GPCPD1	Glycerophospholipid metabolism	REACTOME_PHOSPHOLIPID_METABOLISM
CHAT	Glycerophospholipid metabolism	REACTOME_PHOSPHOLIPID_METABOLISM
ACHE	Glycerophospholipid metabolism	REACTOME_PHOSPHOLIPID_METABOLISM
CHKA	Glycerophospholipid metabolism	REACTOME_PHOSPHOLIPID_METABOLISM
CHKB	Glycerophospholipid metabolism	REACTOME_PHOSPHOLIPID_METABOLISM
PHOSPHO1	Glycerophospholipid metabolism	REACTOME_PHOSPHOLIPID_METABOLISM
PCYT1B	Glycerophospholipid metabolism	REACTOME_PHOSPHOLIPID_METABOLISM
PCYT1A	Glycerophospholipid metabolism	REACTOME_PHOSPHOLIPID_METABOLISM
SELENOI	Ether lipid metabolism	REACTOME_PHOSPHOLIPID_METABOLISM
ETNK1	Glycerophospholipid metabolism	REACTOME_PHOSPHOLIPID_METABOLISM
ETNK2	Glycerophospholipid metabolism	REACTOME_PHOSPHOLIPID_METABOLISM
PCYT2	Glycerophospholipid metabolism	REACTOME_PHOSPHOLIPID_METABOLISM
ETNPPL	Glycerophospholipid metabolism	REACTOME_PHOSPHOLIPID_METABOLISM
PEMT	Glycerophospholipid metabolism	REACTOME_PHOSPHOLIPID_METABOLISM
CDS1	Glycerophospholipid metabolism	REACTOME_PHOSPHOLIPID_METABOLISM
CDS2	Glycerophospholipid metabolism	REACTOME_PHOSPHOLIPID_METABOLISM
PLA1A	Glycerophospholipid metabolism	REACTOME_PHOSPHOLIPID_METABOLISM
PTDSS1	Glycerophospholipid metabolism	REACTOME_PHOSPHOLIPID_METABOLISM
PTDSS2	Glycerophospholipid metabolism	REACTOME_PHOSPHOLIPID_METABOLISM
PISD	Glycerophospholipid metabolism	REACTOME_PHOSPHOLIPID_METABOLISM
PGS1	Glycerophospholipid metabolism	REACTOME_PHOSPHOLIPID_METABOLISM
CRLS1	Glycerophospholipid metabolism	REACTOME_PHOSPHOLIPID_METABOLISM
TAFAZZIN	Glycerophospholipid metabolism	REACTOME_PHOSPHOLIPID_METABOLISM
LPGAT1	Glycerophospholipid metabolism	REACTOME_PHOSPHOLIPID_METABOLISM
MBOAT7	Glycerophospholipid metabolism	REACTOME_PHOSPHOLIPID_METABOLISM
AGPS	Ether lipid metabolism	REACTOME_METABOLISM_OF_LIPIDS
PEDS1	Ether lipid metabolism	Absent
PEDS1-UBE2V1	Ether lipid metabolism	Absent
ENPP6	Ether lipid metabolism	REACTOME_PHOSPHOLIPID_METABOLISM
ENPP2	Ether lipid metabolism	Absent
GDPD3	Ether lipid metabolism	REACTOME_PHOSPHOLIPID_METABOLISM
GDPD1	Ether lipid metabolism	REACTOME_PHOSPHOLIPID_METABOLISM
TMEM86B	Ether lipid metabolism	REACTOME_PHOSPHOLIPID_METABOLISM
UGT8	Sphingolipid metabolism	REACTOME_METABOLISM_OF_LIPIDS
GAL3ST1	Sphingolipid metabolism	Absent
PAFAH1B1	Ether lipid metabolism	Absent
PAFAH1B2	Ether lipid metabolism	Absent
PAFAH1B3	Ether lipid metabolism	Absent
PLA2G7	Ether lipid metabolism	Absent
PAFAH2	Ether lipid metabolism	Absent
SPTLC1	Sphingolipid metabolism	REACTOME_METABOLISM_OF_LIPIDS
SPTLC2	Sphingolipid metabolism	REACTOME_METABOLISM_OF_LIPIDS
SPTLC3	Sphingolipid metabolism	REACTOME_METABOLISM_OF_LIPIDS
KDSR	Sphingolipid metabolism	REACTOME_METABOLISM_OF_LIPIDS
CERS1	Sphingolipid metabolism	REACTOME_METABOLISM_OF_LIPIDS
CERS2	Sphingolipid metabolism	REACTOME_METABOLISM_OF_LIPIDS
CERS4	Sphingolipid metabolism	REACTOME_METABOLISM_OF_LIPIDS
CERS3	Sphingolipid metabolism	REACTOME_METABOLISM_OF_LIPIDS
CERS6	Sphingolipid metabolism	REACTOME_METABOLISM_OF_LIPIDS
CERS5	Sphingolipid metabolism	REACTOME_METABOLISM_OF_LIPIDS
ASAH1	Sphingolipid metabolism	REACTOME_METABOLISM_OF_LIPIDS
ASAH2	Sphingolipid metabolism	REACTOME_METABOLISM_OF_LIPIDS

ACER2	Sphingolipid metabolism	REACTOME_METABOLISM_OF_LIPIDS
ACER1	Sphingolipid metabolism	REACTOME_METABOLISM_OF_LIPIDS
ACER3	Sphingolipid metabolism	REACTOME_METABOLISM_OF_LIPIDS
DEGS1	Sphingolipid metabolism	REACTOME_METABOLISM_OF_LIPIDS
DEGS2	Sphingolipid metabolism	REACTOME_METABOLISM_OF_LIPIDS
SGMS1	Sphingolipid metabolism	REACTOME_METABOLISM_OF_LIPIDS
SGMS2	Sphingolipid metabolism	REACTOME_METABOLISM_OF_LIPIDS
SMPD1	Sphingolipid metabolism	REACTOME_METABOLISM_OF_LIPIDS
SMPD2	Sphingolipid metabolism	REACTOME_METABOLISM_OF_LIPIDS
SMPD3	Sphingolipid metabolism	REACTOME_METABOLISM_OF_LIPIDS
SMPD4	Sphingolipid metabolism	REACTOME_METABOLISM_OF_LIPIDS
ENPP7	Sphingolipid metabolism	REACTOME_METABOLISM_OF_LIPIDS
CERK	Sphingolipid metabolism	REACTOME_METABOLISM_OF_LIPIDS
SGPP1	Sphingolipid metabolism	REACTOME_METABOLISM_OF_LIPIDS
SGPP2	Sphingolipid metabolism	REACTOME_METABOLISM_OF_LIPIDS
SPHK1	Sphingolipid metabolism	REACTOME_METABOLISM_OF_LIPIDS
SPHK2	Sphingolipid metabolism	REACTOME_METABOLISM_OF_LIPIDS
SGPL1	Sphingolipid metabolism	REACTOME_METABOLISM_OF_LIPIDS
UGCG	Sphingolipid metabolism	REACTOME_METABOLISM_OF_LIPIDS
GBA	Other glycan degradation	REACTOME_METABOLISM_OF_LIPIDS
GBA2	Other glycan degradation	REACTOME_METABOLISM_OF_LIPIDS
B4GALT6	Sphingolipid metabolism	REACTOME_METABOLISM_OF_CARBOHYDRATES
GALC	Sphingolipid metabolism	REACTOME_METABOLISM_OF_LIPIDS
ARSA	Sphingolipid metabolism	REACTOME_METABOLISM_OF_LIPIDS
NEU1	Other glycan degradation	REACTOME_METABOLISM_OF_LIPIDS
NEU3	Other glycan degradation	REACTOME_METABOLISM_OF_LIPIDS
NEU4	Other glycan degradation	REACTOME_METABOLISM_OF_LIPIDS
NEU2	Other glycan degradation	REACTOME_METABOLISM_OF_LIPIDS
PSAP	Sphingolipid metabolism	REACTOME_METABOLISM_OF_LIPIDS
PSAPL1	Sphingolipid metabolism	Absent
PTGS1	Arachidonic acid metabolism	REACTOME_METABOLISM_OF_LIPIDS
PTGS2	Arachidonic acid metabolism	REACTOME_METABOLISM_OF_LIPIDS
PTGES	Arachidonic acid metabolism	REACTOME_METABOLISM_OF_LIPIDS
PTGES2	Arachidonic acid metabolism	REACTOME_METABOLISM_OF_LIPIDS
PTGES3	Arachidonic acid metabolism	REACTOME_METABOLISM_OF_LIPIDS
CBR1	Arachidonic acid metabolism	HALLMARK_FATTY_ACID_METABOLISM
CBR3	Arachidonic acid metabolism	HALLMARK_FATTY_ACID_METABOLISM
PRXL2B	Arachidonic acid metabolism	REACTOME_METABOLISM_OF_LIPIDS
TBXAS1	Arachidonic acid metabolism	REACTOME_METABOLISM_OF_LIPIDS
PTGDS	Arachidonic acid metabolism	REACTOME_METABOLISM_OF_LIPIDS
HPGDS	Arachidonic acid metabolism	REACTOME_METABOLISM_OF_LIPIDS
PTGIS	Arachidonic acid metabolism	REACTOME_METABOLISM_OF_LIPIDS
ALOX5	Arachidonic acid metabolism	REACTOME_METABOLISM_OF_LIPIDS
LTA4H	Arachidonic acid metabolism	REACTOME_METABOLISM_OF_LIPIDS
CYP4F2	Arachidonic acid metabolism	REACTOME_METABOLISM_OF_LIPIDS
CYP4F3	Arachidonic acid metabolism	REACTOME_METABOLISM_OF_LIPIDS
LTC4S	Arachidonic acid metabolism	HALLMARK_FATTY_ACID_METABOLISM
GGT1	Arachidonic acid metabolism	REACTOME_METABOLISM_OF_LIPIDS
GGT5	Arachidonic acid metabolism	REACTOME_METABOLISM_OF_LIPIDS
GPX6	Arachidonic acid metabolism	Absent
GPX7	Arachidonic acid metabolism	Absent
GPX2	Arachidonic acid metabolism	REACTOME_METABOLISM_OF_LIPIDS
GPX3	Arachidonic acid metabolism	Absent
GPX1	Arachidonic acid metabolism	REACTOME_METABOLISM_OF_LIPIDS
GPX5	Arachidonic acid metabolism	Absent
GPX8	Arachidonic acid metabolism	Absent
CYP2J2	Linoleic acid metabolism	REACTOME_METABOLISM_OF_LIPIDS
CYP2C19	Linoleic acid metabolism	REACTOME_METABOLISM_OF_LIPIDS

CYP4F8	Arachidonic acid metabolism	REACTOME_METABOLISM_OF_LIPIDS
ALOX12	Arachidonic acid metabolism	REACTOME_METABOLISM_OF_LIPIDS
ALOX12B	Arachidonic acid metabolism	REACTOME_METABOLISM_OF_LIPIDS
ALOX15B	Arachidonic acid metabolism	REACTOME_METABOLISM_OF_LIPIDS
CYP2B6	Arachidonic acid metabolism	Absent
CYP2C8	Linoleic acid metabolism	REACTOME_METABOLISM_OF_LIPIDS
CYP2C9	Linoleic acid metabolism	REACTOME_METABOLISM_OF_LIPIDS
EPHX2	Arachidonic acid metabolism	REACTOME_METABOLISM_OF_LIPIDS
ALOX15	Linoleic acid metabolism	REACTOME_METABOLISM_OF_LIPIDS
FADS2	Biosynthesis of unsaturated fatty acids	REACTOME_METABOLISM_OF_LIPIDS
SCD	Biosynthesis of unsaturated fatty acids	REACTOME_METABOLISM_OF_LIPIDS
SCD5	Biosynthesis of unsaturated fatty acids	REACTOME_METABOLISM_OF_LIPIDS
FADS1	Biosynthesis of unsaturated fatty acids	REACTOME_METABOLISM_OF_LIPIDS
DOLK	N Glycan biosynthesis	Absent
DPAGT1	N Glycan biosynthesis	Absent
ALG5	N Glycan biosynthesis	Absent
ALG13	Various types of N glycan biosynthesis	Absent
ALG14	Various types of N glycan biosynthesis	Absent
DPM1	N Glycan biosynthesis	Absent
DPM2	Glycosylphosphatidylinositol anchor biosynthesis	Absent
DPM3	N Glycan biosynthesis	Absent
ALG1	Various types of N glycan biosynthesis	Absent
ALG2	Various types of N glycan biosynthesis	Absent
ALG11	Various types of N glycan biosynthesis	Absent
ALG3	Various types of N glycan biosynthesis	Absent
ALG9	Various types of N glycan biosynthesis	Absent
ALG12	Various types of N glycan biosynthesis	Absent
ALG6	N Glycan biosynthesis	Absent
ALG8	N Glycan biosynthesis	Absent
ALG10	N Glycan biosynthesis	Absent
ALG10B	N Glycan biosynthesis	Absent
STT3A	Various types of N glycan biosynthesis	Absent
STT3B	Various types of N glycan biosynthesis	Absent
RPN1	Various types of N glycan biosynthesis	Absent
RPN2	Various types of N glycan biosynthesis	Absent
DAD1	Various types of N glycan biosynthesis	Absent
TUSC3	Various types of N glycan biosynthesis	Absent
DDOST	Various types of N glycan biosynthesis	Absent
DOLPP1	N Glycan biosynthesis	Absent
MOGS	N Glycan biosynthesis	Absent
GANAB	N Glycan biosynthesis	Absent
MAN1B1	Various types of N glycan biosynthesis	Absent
MAN1A2	Various types of N glycan biosynthesis	Absent
MAN1C1	Various types of N glycan biosynthesis	Absent
MAN1A1	Various types of N glycan biosynthesis	Absent
MGAT1	Various types of N glycan biosynthesis	Absent
MAN2A1	Various types of N glycan biosynthesis	Absent
MAN2A2	Various types of N glycan biosynthesis	Absent
MGAT2	Various types of N glycan biosynthesis	Absent
FUT8	Glycosaminoglycan biosynthesis keratan sulfate	Absent
B4GALT3	Glycosphingolipid biosynthesis lacto and neolacto series	REACTOME_METABOLISM_OF_CARBOHYDRATES
ST6GAL1	Other types of O glycan biosynthesis	Absent
ST6GAL2	Other types of O glycan biosynthesis	Absent
MGAT3	N Glycan biosynthesis	Absent
MGAT4A	Various types of N glycan biosynthesis	Absent
MGAT4B	Various types of N glycan biosynthesis	Absent

MGAT4D	Various types of N glycan biosynthesis	Absent
MGAT5	N Glycan biosynthesis	Absent
MGAT5B	Mannose type O glycan biosynthesis	Absent
MGAT4C	Various types of N glycan biosynthesis	Absent
HEXD	Other glycan degradation	Absent
ST3GAL3	Glycosphingolipid biosynthesis lacto and neolacto series	REACTOME_METABOLISM_OF_CARBOHYDRATES
B4GALNT3	Various types of N glycan biosynthesis	Absent
B4GALNT4	Various types of N glycan biosynthesis	Absent
CHST8	Various types of N glycan biosynthesis	Absent
CHST9	Various types of N glycan biosynthesis	REACTOME_METABOLISM_OF_CARBOHYDRATES
GALNTL6	Other types of O glycan biosynthesis	Absent
GALNT5	Other types of O glycan biosynthesis	Absent
GALNT17	Other types of O glycan biosynthesis	Absent
GALNT11	Other types of O glycan biosynthesis	Absent
GALNT12	Other types of O glycan biosynthesis	Absent
GALNT13	Other types of O glycan biosynthesis	Absent
GALNT14	Other types of O glycan biosynthesis	Absent
GALNT16	Other types of O glycan biosynthesis	Absent
GALNT15	Other types of O glycan biosynthesis	Absent
GALNT18	Other types of O glycan biosynthesis	Absent
GALNTL5	Other types of O glycan biosynthesis	Absent
GALNT10	Other types of O glycan biosynthesis	Absent
GALNT2	Other types of O glycan biosynthesis	Absent
GALNT3	Other types of O glycan biosynthesis	Absent
GALNT1	Other types of O glycan biosynthesis	Absent
GALNT6	Other types of O glycan biosynthesis	Absent
GALNT4	Other types of O glycan biosynthesis	Absent
GALNT9	Other types of O glycan biosynthesis	Absent
GALNT7	Other types of O glycan biosynthesis	Absent
GALNT8	Other types of O glycan biosynthesis	Absent
C1GALT1	Other types of O glycan biosynthesis	Absent
C1GALT1C1	Other types of O glycan biosynthesis	Absent
C1GALT1C1L	Other types of O glycan biosynthesis	Absent
GCNT1	Mucin type O glycan biosynthesis	Absent
GCNT3	Mucin type O glycan biosynthesis	Absent
GCNT4	Mucin type O glycan biosynthesis	Absent
ST3GAL1	Glycosphingolipid biosynthesis ganglio series	REACTOME_METABOLISM_OF_CARBOHYDRATES
ST3GAL2	Glycosphingolipid biosynthesis ganglio series	REACTOME_METABOLISM_OF_CARBOHYDRATES
ST6GALNAC1	Mucin type O glycan biosynthesis	Absent
ST6GALNAC2	Mucin type O glycan biosynthesis	Absent
ST6GALNAC3	Glycosphingolipid biosynthesis ganglio series	Absent
ST6GALNAC4	Glycosphingolipid biosynthesis ganglio series	Absent
B3GNT3	Glycosphingolipid biosynthesis lacto and neolacto series	REACTOME_METABOLISM_OF_CARBOHYDRATES
B3GNT6	Mucin type O glycan biosynthesis	Absent
B4GALT5	Mucin type O glycan biosynthesis	REACTOME_METABOLISM_OF_CARBOHYDRATES
POMT1	Other types of O glycan biosynthesis	Absent
POMT2	Other types of O glycan biosynthesis	Absent
POMGNT1	Mannose type O glycan biosynthesis	Absent
B3GAT1	Mannose type O glycan biosynthesis	REACTOME_METABOLISM_OF_CARBOHYDRATES
B3GAT2	Mannose type O glycan biosynthesis	REACTOME_METABOLISM_OF_CARBOHYDRATES
CHST10	Mannose type O glycan biosynthesis	Absent
FUT9	Glycosphingolipid biosynthesis globo and isoglobo series	REACTOME_METABOLISM_OF_CARBOHYDRATES
FUT4	Glycosphingolipid biosynthesis lacto and neolacto series	REACTOME_METABOLISM_OF_CARBOHYDRATES
POMGNT2	Mannose type O glycan biosynthesis	Absent

B3GALNT2	Mannose type O glycan biosynthesis	Absent
POMK	Mannose type O glycan biosynthesis	Absent
CRPPA	Mannose type O glycan biosynthesis	Absent
FKTN	Mannose type O glycan biosynthesis	Absent
FKRP	Mannose type O glycan biosynthesis	Absent
RXYLT1	Mannose type O glycan biosynthesis	Absent
B4GAT1	Mannose type O glycan biosynthesis	REACTOME_METABOLISM_OF_CARBOHYDRATES
LARGE1	Mannose type O glycan biosynthesis	Absent
LARGE2	Mannose type O glycan biosynthesis	Absent
OGT	Other types of O glycan biosynthesis	Absent
EOGT	Other types of O glycan biosynthesis	Absent
POFUT1	Other types of O glycan biosynthesis	Absent
POFUT2	Other types of O glycan biosynthesis	Absent
MFNG	Other types of O glycan biosynthesis	Absent
LFNG	Other types of O glycan biosynthesis	Absent
RFNG	Other types of O glycan biosynthesis	Absent
B3GLCT	Other types of O glycan biosynthesis	Absent
POGLUT1	Other types of O glycan biosynthesis	Absent
GXYLT1	Other types of O glycan biosynthesis	Absent
GXYLT2	Other types of O glycan biosynthesis	Absent
XXYLT1	Other types of O glycan biosynthesis	Absent
XYLT1	Glycosaminoglycan biosynthesis heparan sulfate heparin	REACTOME_METABOLISM_OF_CARBOHYDRATES
XYLT2	Glycosaminoglycan biosynthesis heparan sulfate heparin	REACTOME_METABOLISM_OF_CARBOHYDRATES
B4GALT7	Glycosaminoglycan biosynthesis heparan sulfate heparin	REACTOME_METABOLISM_OF_CARBOHYDRATES
B3GALT6	Glycosaminoglycan biosynthesis heparan sulfate heparin	REACTOME_METABOLISM_OF_CARBOHYDRATES
B3GAT3	Glycosaminoglycan biosynthesis heparan sulfate heparin	REACTOME_METABOLISM_OF_CARBOHYDRATES
CSGALNACT1	Glycosaminoglycan biosynthesis chondroitin sulfate dermatan sulfate	REACTOME_METABOLISM_OF_CARBOHYDRATES
CSGALNACT2	Glycosaminoglycan biosynthesis chondroitin sulfate dermatan sulfate	REACTOME_METABOLISM_OF_CARBOHYDRATES
CHSY3	Glycosaminoglycan biosynthesis chondroitin sulfate dermatan sulfate	REACTOME_METABOLISM_OF_CARBOHYDRATES
CHSY1	Glycosaminoglycan biosynthesis chondroitin sulfate dermatan sulfate	REACTOME_METABOLISM_OF_CARBOHYDRATES
CHPF	Glycosaminoglycan biosynthesis chondroitin sulfate dermatan sulfate	REACTOME_METABOLISM_OF_CARBOHYDRATES
CHPF2	Glycosaminoglycan biosynthesis chondroitin sulfate dermatan sulfate	REACTOME_METABOLISM_OF_CARBOHYDRATES
DSE	Glycosaminoglycan biosynthesis chondroitin sulfate dermatan sulfate	REACTOME_METABOLISM_OF_CARBOHYDRATES
CHST11	Glycosaminoglycan biosynthesis chondroitin sulfate dermatan sulfate	REACTOME_METABOLISM_OF_CARBOHYDRATES
CHST12	Glycosaminoglycan biosynthesis chondroitin sulfate dermatan sulfate	REACTOME_METABOLISM_OF_CARBOHYDRATES
CHST13	Glycosaminoglycan biosynthesis chondroitin sulfate dermatan sulfate	REACTOME_METABOLISM_OF_CARBOHYDRATES
CHST3	Glycosaminoglycan biosynthesis chondroitin sulfate dermatan sulfate	REACTOME_METABOLISM_OF_CARBOHYDRATES
CHST7	Glycosaminoglycan biosynthesis chondroitin sulfate dermatan sulfate	REACTOME_METABOLISM_OF_CARBOHYDRATES
CHST15	Glycosaminoglycan biosynthesis chondroitin sulfate dermatan sulfate	REACTOME_METABOLISM_OF_CARBOHYDRATES
UST	Glycosaminoglycan biosynthesis chondroitin sulfate dermatan sulfate	REACTOME_METABOLISM_OF_CARBOHYDRATES
CHST14	Glycosaminoglycan biosynthesis chondroitin sulfate dermatan sulfate	REACTOME_METABOLISM_OF_CARBOHYDRATES

EXTL2	Glycosaminoglycan biosynthesis heparan sulfate heparin	Absent
EXTL3	Glycosaminoglycan biosynthesis heparan sulfate heparin	Absent
EXTL1	Glycosaminoglycan biosynthesis heparan sulfate heparin	Absent
EXT1	Glycosaminoglycan biosynthesis heparan sulfate heparin	REACTOME_METABOLISM_OF_CARBOHYDRATES
EXT2	Glycosaminoglycan biosynthesis heparan sulfate heparin	REACTOME_METABOLISM_OF_CARBOHYDRATES
NDST1	Glycosaminoglycan biosynthesis heparan sulfate heparin	REACTOME_METABOLISM_OF_CARBOHYDRATES
NDST2	Glycosaminoglycan biosynthesis heparan sulfate heparin	REACTOME_METABOLISM_OF_CARBOHYDRATES
NDST3	Glycosaminoglycan biosynthesis heparan sulfate heparin	REACTOME_METABOLISM_OF_CARBOHYDRATES
NDST4	Glycosaminoglycan biosynthesis heparan sulfate heparin	REACTOME_METABOLISM_OF_CARBOHYDRATES
GLCE	Glycosaminoglycan biosynthesis heparan sulfate heparin	REACTOME_METABOLISM_OF_CARBOHYDRATES
HS2ST1	Glycosaminoglycan biosynthesis heparan sulfate heparin	REACTOME_METABOLISM_OF_CARBOHYDRATES
HS6ST1	Glycosaminoglycan biosynthesis heparan sulfate heparin	REACTOME_METABOLISM_OF_CARBOHYDRATES
HS6ST2	Glycosaminoglycan biosynthesis heparan sulfate heparin	REACTOME_METABOLISM_OF_CARBOHYDRATES
HS6ST3	Glycosaminoglycan biosynthesis heparan sulfate heparin	REACTOME_METABOLISM_OF_CARBOHYDRATES
HS3ST1	Glycosaminoglycan biosynthesis heparan sulfate heparin	REACTOME_METABOLISM_OF_CARBOHYDRATES
HS3ST2	Glycosaminoglycan biosynthesis heparan sulfate heparin	REACTOME_METABOLISM_OF_CARBOHYDRATES
HS3ST3B1	Glycosaminoglycan biosynthesis heparan sulfate heparin	REACTOME_METABOLISM_OF_CARBOHYDRATES
HS3ST3A1	Glycosaminoglycan biosynthesis heparan sulfate heparin	REACTOME_METABOLISM_OF_CARBOHYDRATES
HS3ST5	Glycosaminoglycan biosynthesis heparan sulfate heparin	REACTOME_METABOLISM_OF_CARBOHYDRATES
B3GNT2	Glycosphingolipid biosynthesis lacto and neolacto series	REACTOME_METABOLISM_OF_CARBOHYDRATES
CHST6	Glycosaminoglycan biosynthesis keratan sulfate	REACTOME_METABOLISM_OF_CARBOHYDRATES
B4GALT4	Glycosphingolipid biosynthesis lacto and neolacto series	REACTOME_METABOLISM_OF_CARBOHYDRATES
B3GNT7	Glycosaminoglycan biosynthesis keratan sulfate	REACTOME_METABOLISM_OF_CARBOHYDRATES
CHST1	Glycosaminoglycan biosynthesis keratan sulfate	REACTOME_METABOLISM_OF_CARBOHYDRATES
CHST2	Glycosaminoglycan biosynthesis keratan sulfate	REACTOME_METABOLISM_OF_CARBOHYDRATES
CHST4	Glycosaminoglycan biosynthesis keratan sulfate	Absent
HYAL2	Glycosaminoglycan degradation	REACTOME_METABOLISM_OF_CARBOHYDRATES
HYAL1	Glycosaminoglycan degradation	REACTOME_METABOLISM_OF_CARBOHYDRATES
SPAM1	Glycosaminoglycan degradation	Absent
HYAL4	Glycosaminoglycan degradation	Absent
HYAL3	Glycosaminoglycan degradation	REACTOME_METABOLISM_OF_CARBOHYDRATES

IDS	Glycosaminoglycan degradation	REACTOME_METABOLISM_OF_CARBOHYDRATES
IDUA	Glycosaminoglycan degradation	REACTOME_METABOLISM_OF_CARBOHYDRATES
ARSB	Glycosaminoglycan degradation	REACTOME_METABOLISM_OF_LIPIDS
HPSE	Glycosaminoglycan degradation	REACTOME_METABOLISM_OF_CARBOHYDRATES
HPSE2	Glycosaminoglycan degradation	REACTOME_METABOLISM_OF_CARBOHYDRATES
SGSH	Glycosaminoglycan degradation	REACTOME_METABOLISM_OF_CARBOHYDRATES
HGSNAT	Glycosaminoglycan degradation	REACTOME_METABOLISM_OF_CARBOHYDRATES
NAGLU	Glycosaminoglycan degradation	REACTOME_METABOLISM_OF_CARBOHYDRATES
GALNS	Glycosaminoglycan degradation	REACTOME_METABOLISM_OF_CARBOHYDRATES
GNS	Glycosaminoglycan degradation	REACTOME_METABOLISM_OF_CARBOHYDRATES
PIGA	Glycosylphosphatidylinositol anchor biosynthesis	Absent
PIGC	Glycosylphosphatidylinositol anchor biosynthesis	Absent
PIGH	Glycosylphosphatidylinositol anchor biosynthesis	Absent
PIGP	Glycosylphosphatidylinositol anchor biosynthesis	Absent
PIGQ	Glycosylphosphatidylinositol anchor biosynthesis	Absent
PIGY	Glycosylphosphatidylinositol anchor biosynthesis	Absent
PIGL	Glycosylphosphatidylinositol anchor biosynthesis	Absent
PIGW	Glycosylphosphatidylinositol anchor biosynthesis	Absent
GPLD1	Glycosylphosphatidylinositol anchor biosynthesis	Absent
PIGM	Glycosylphosphatidylinositol anchor biosynthesis	Absent
PIGX	Glycosylphosphatidylinositol anchor biosynthesis	Absent
PIGV	Glycosylphosphatidylinositol anchor biosynthesis	Absent
PIGN	Glycosylphosphatidylinositol anchor biosynthesis	Absent
PIGB	Glycosylphosphatidylinositol anchor biosynthesis	Absent
PIGO	Glycosylphosphatidylinositol anchor biosynthesis	Absent
PIGF	Glycosylphosphatidylinositol anchor biosynthesis	Absent
PIGG	Glycosylphosphatidylinositol anchor biosynthesis	Absent
GPAA1	Glycosylphosphatidylinositol anchor biosynthesis	Absent
PIGK	Glycosylphosphatidylinositol anchor biosynthesis	Absent
PIGS	Glycosylphosphatidylinositol anchor biosynthesis	Absent
PIGT	Glycosylphosphatidylinositol anchor biosynthesis	Absent

PIGU	Glycosylphosphatidylinositol anchor biosynthesis	Absent
PGAP1	Glycosylphosphatidylinositol anchor biosynthesis	Absent
MPPE1	Glycosylphosphatidylinositol anchor biosynthesis	Absent
PIGZ	Glycosylphosphatidylinositol anchor biosynthesis	Absent
B3GNT5	Glycosphingolipid biosynthesis lacto and neolacto series	Absent
B3GALT1	Glycosphingolipid biosynthesis lacto and neolacto series	REACTOME_METABOLISM_OF_CARBOHYDRATES
B3GALT2	Glycosphingolipid biosynthesis lacto and neolacto series	REACTOME_METABOLISM_OF_CARBOHYDRATES
B3GALT5	Glycosphingolipid biosynthesis globo and isoglobo series	REACTOME_METABOLISM_OF_CARBOHYDRATES
FUT1	Glycosphingolipid biosynthesis globo and isoglobo series	REACTOME_METABOLISM_OF_CARBOHYDRATES
FUT2	Glycosphingolipid biosynthesis globo and isoglobo series	REACTOME_METABOLISM_OF_CARBOHYDRATES
FUT3	Glycosphingolipid biosynthesis lacto and neolacto series	REACTOME_METABOLISM_OF_CARBOHYDRATES
ST3GAL4	Glycosphingolipid biosynthesis lacto and neolacto series	REACTOME_METABOLISM_OF_CARBOHYDRATES
ABO	Glycosphingolipid biosynthesis lacto and neolacto series	Absent
FUT5	Glycosphingolipid biosynthesis lacto and neolacto series	REACTOME_METABOLISM_OF_CARBOHYDRATES
FUT6	Glycosphingolipid biosynthesis lacto and neolacto series	REACTOME_METABOLISM_OF_CARBOHYDRATES
FUT7	Glycosphingolipid biosynthesis lacto and neolacto series	REACTOME_METABOLISM_OF_CARBOHYDRATES
ST3GAL6	Glycosphingolipid biosynthesis lacto and neolacto series	REACTOME_METABOLISM_OF_CARBOHYDRATES
ST8SIA1	Glycosphingolipid biosynthesis ganglio series	Absent
A4GALT	Glycosphingolipid biosynthesis globo and isoglobo series	Absent
B3GALNT1	Glycosphingolipid biosynthesis globo and isoglobo series	REACTOME_METABOLISM_OF_LIPIDS
B3GNT4	Glycosphingolipid biosynthesis lacto and neolacto series	REACTOME_METABOLISM_OF_CARBOHYDRATES
GCNT2	Glycosphingolipid biosynthesis lacto and neolacto series	Absent
A3GALT2	Glycosphingolipid biosynthesis globo and isoglobo series	Absent
GBGT1	Glycosphingolipid biosynthesis globo and isoglobo series	Absent
NAGA	Glycosphingolipid biosynthesis globo and isoglobo series	Absent
B4GALNT1	Glycosphingolipid biosynthesis ganglio	REACTOME_METABOLISM_OF_LIPIDS
B3GALT4	Glycosphingolipid biosynthesis ganglio	REACTOME_METABOLISM_OF_CARBOHYDRATES
ST8SIA5	Glycosphingolipid biosynthesis ganglio	Absent
ST3GAL5	Glycosphingolipid biosynthesis ganglio	Absent
SLC33A1	Glycosphingolipid biosynthesis ganglio	Absent
ST6GALNAC5	Glycosphingolipid biosynthesis ganglio	Absent
ST6GALNAC6	Glycosphingolipid biosynthesis ganglio series	REACTOME_METABOLISM_OF_CARBOHYDRATES
MAN2C1	Other glycan degradation	REACTOME_METABOLISM_OF_CARBOHYDRATES
MAN2B1	Other glycan degradation	REACTOME_METABOLISM_OF_CARBOHYDRATES
MAN2B2	Other glycan degradation	REACTOME_METABOLISM_OF_CARBOHYDRATES
MANBA	Other glycan degradation	REACTOME_METABOLISM_OF_CARBOHYDRATES
ENGASE	Other glycan degradation	Absent

FUCA1	Other glycan degradation	Absent
FUCA2	Other glycan degradation	Absent
AGA	Other glycan degradation	Absent
NUDT9	Purine metabolism	Absent
NUDT5	Purine metabolism	Absent
PRPS1L1	Purine metabolism	REACTOME_METABOLISM_OF_CARBOHYDRATES
PRPS2	Purine metabolism	REACTOME_METABOLISM_OF_CARBOHYDRATES
PRPS1	Purine metabolism	REACTOME_METABOLISM_OF_CARBOHYDRATES
GART	Purine metabolism	Absent
PFAS	Purine metabolism	Absent
PAICS	Purine metabolism	Absent
ATIC	Purine metabolism	Absent
APRT	Purine metabolism	Absent
NT5C2	Pyrimidine metabolism	Absent
NT5C1A	Pyrimidine metabolism	Absent
NT5C1B	Pyrimidine metabolism	Absent
NT5C	Pyrimidine metabolism	Absent
NT5M	Pyrimidine metabolism	Absent
NT5E	Pyrimidine metabolism	Absent
PNP	Pyrimidine metabolism	Absent
HPRT1	Purine metabolism	Absent
IMPDH1	Purine metabolism	Absent
IMPDH2	Purine metabolism	Absent
NME6	Pyrimidine metabolism	Absent
NME7	Pyrimidine metabolism	Absent
NME2	Pyrimidine metabolism	Absent
NME4	Pyrimidine metabolism	Absent
NME1	Pyrimidine metabolism	Absent
NME3	Pyrimidine metabolism	Absent
NME1-NME2	Pyrimidine metabolism	Absent
AK9	Pyrimidine metabolism	Absent
ENTPD3	Pyrimidine metabolism	Absent
ENTPD8	Pyrimidine metabolism	Absent
ENTPD1	Pyrimidine metabolism	Absent
CANT1	Pyrimidine metabolism	Absent
ENTPD4	Pyrimidine metabolism	Absent
ENTPD5	Pyrimidine metabolism	Absent
ENTPD6	Pyrimidine metabolism	Absent
NUDT16	Purine metabolism	Absent
ITPA	Purine metabolism	Absent
XDH	Purine metabolism	Absent
NUDT2	Pyrimidine metabolism	Absent
GMPS	Purine metabolism	Absent
GMPR	Purine metabolism	Absent
GMPR2	Purine metabolism	Absent
GDA	Purine metabolism	Absent
GUK1	Purine metabolism	Absent
RRM1	Pyrimidine metabolism	Absent
RRM2B	Pyrimidine metabolism	Absent
RRM2	Pyrimidine metabolism	Absent
HDDC2	Pyrimidine metabolism	Absent
DGUOK	Purine metabolism	Absent
DCK	Pyrimidine metabolism	Absent
HDDC3	Purine metabolism	Absent
PRUNE1	Purine metabolism	Absent
ADCY1	Purine metabolism	Absent
ADCY2	Purine metabolism	Absent
ADCY3	Purine metabolism	Absent

ADCY4	Purine metabolism	Absent
ADCY5	Purine metabolism	Absent
ADCY6	Purine metabolism	Absent
ADCY7	Purine metabolism	Absent
ADCY8	Purine metabolism	Absent
ADCY9	Purine metabolism	Absent
ADCY10	Purine metabolism	Absent
GUCY1A2	Purine metabolism	Absent
GUCY1A1	Purine metabolism	Absent
GUCY1B1	Purine metabolism	Absent
GUCY2C	Purine metabolism	Absent
GUCY2D	Purine metabolism	Absent
GUCY2F	Purine metabolism	Absent
NPR1	Purine metabolism	Absent
NPR2	Purine metabolism	Absent
PDE1A	Purine metabolism	Absent
PDE1B	Purine metabolism	Absent
PDE1C	Purine metabolism	Absent
PDE2A	Purine metabolism	Absent
PDE3A	Purine metabolism	Absent
PDE3B	Purine metabolism	Absent
PDE5A	Purine metabolism	Absent
PDE6A	Purine metabolism	Absent
PDE6B	Purine metabolism	Absent
PDE6C	Purine metabolism	Absent
PDE6D	Purine metabolism	Absent
PDE6G	Purine metabolism	Absent
PDE6H	Purine metabolism	Absent
PDE9A	Purine metabolism	Absent
PDE10A	Purine metabolism	Absent
PDE11A	Purine metabolism	Absent
AMPD2	Purine metabolism	Absent
AMPD3	Purine metabolism	Absent
AMPD1	Purine metabolism	Absent
ADK	Purine metabolism	Absent
ADA	Purine metabolism	Absent
ADA2	Purine metabolism	Absent
AK7	Purine metabolism	Absent
AK4	Purine metabolism	Absent
AK5	Purine metabolism	Absent
AK2	Purine metabolism	Absent
AK1	Purine metabolism	Absent
AK8	Purine metabolism	Absent
AK3	Purine metabolism	Absent
ENTPD2	Purine metabolism	Absent
NTPCR	Purine metabolism	Absent
PDE4A	Purine metabolism	Absent
PDE4B	Purine metabolism	Absent
PDE4C	Purine metabolism	Absent
PDE4D	Purine metabolism	Absent
PDE7A	Purine metabolism	Absent
PDE7B	Purine metabolism	Absent
PDE8B	Purine metabolism	Absent
PDE8A	Purine metabolism	Absent
FHIT	Purine metabolism	Absent
ENPP4	Purine metabolism	Absent
URAD	Purine metabolism	Absent
ALLC	Purine metabolism	Absent

DHODH	Pyrimidine metabolism	Absent
UMPS	Pyrimidine metabolism	Absent
CMPK1	Pyrimidine metabolism	Absent
CMPK2	Pyrimidine metabolism	Absent
ASMTL	Pyrimidine metabolism	Absent
CTPS1	Pyrimidine metabolism	Absent
CTPS2	Pyrimidine metabolism	Absent
UCK1	Pyrimidine metabolism	Absent
UCK2	Pyrimidine metabolism	Absent
UCKL1	Pyrimidine metabolism	Absent
UPP2	Pyrimidine metabolism	Absent
UPP1	Pyrimidine metabolism	Absent
DPYD	Pyrimidine metabolism	Absent
DPYS	Pyrimidine metabolism	Absent
UPB1	Pyrimidine metabolism	Absent
NT5C3A	Pyrimidine metabolism	Absent
NT5C3B	Pyrimidine metabolism	Absent
CDA	Pyrimidine metabolism	Absent
DCTPP1	Pyrimidine metabolism	Absent
DCTD	Pyrimidine metabolism	Absent
DTYMK	Pyrimidine metabolism	Absent
DUT	Pyrimidine metabolism	Absent
TYMP	Pyrimidine metabolism	Absent
TK2	Pyrimidine metabolism	Absent
TK1	Pyrimidine metabolism	Absent
TYMS	Pyrimidine metabolism	Absent
ADIPOR2	Absent	HALLMARK_FATTY_ACID_METABOLISM
ALAD	Absent	HALLMARK_FATTY_ACID_METABOLISM
ALDH1A1	Absent	HALLMARK_FATTY_ACID_METABOLISM
ALDOA	Fructose and mannose metabolism	HALLMARK_FATTY_ACID_METABOLISM
APEX1	Absent	HALLMARK_FATTY_ACID_METABOLISM
AQP7	Absent	HALLMARK_FATTY_ACID_METABOLISM
AUH	Absent	HALLMARK_FATTY_ACID_METABOLISM
BLVRA	Absent	HALLMARK_FATTY_ACID_METABOLISM
BMPR1B	Absent	HALLMARK_FATTY_ACID_METABOLISM
BPHL	Absent	HALLMARK_FATTY_ACID_METABOLISM
CD1D	Absent	HALLMARK_FATTY_ACID_METABOLISM
CD36	Absent	HALLMARK_FATTY_ACID_METABOLISM
CIDEA	Absent	HALLMARK_FATTY_ACID_METABOLISM
CPOX	Absent	HALLMARK_FATTY_ACID_METABOLISM
CRAT	Absent	HALLMARK_FATTY_ACID_METABOLISM
CRYZ	Absent	HALLMARK_FATTY_ACID_METABOLISM
CYP4A11	Absent	HALLMARK_FATTY_ACID_METABOLISM
CYP4A22	Absent	HALLMARK_FATTY_ACID_METABOLISM
D2HGDH	Absent	HALLMARK_FATTY_ACID_METABOLISM
DECR1	Absent	HALLMARK_FATTY_ACID_METABOLISM
ECH1	Absent	HALLMARK_FATTY_ACID_METABOLISM
ENO2	Glycolysis Gluconeogenesis	HALLMARK_FATTY_ACID_METABOLISM
ENO3	Glycolysis Gluconeogenesis	HALLMARK_FATTY_ACID_METABOLISM
EPHX1	Absent	HALLMARK_FATTY_ACID_METABOLISM
ERP29	Absent	HALLMARK_FATTY_ACID_METABOLISM
ETFDH	Absent	HALLMARK_FATTY_ACID_METABOLISM
FABP1	Absent	HALLMARK_FATTY_ACID_METABOLISM
FABP2	Absent	HALLMARK_FATTY_ACID_METABOLISM
FMO1	Absent	HALLMARK_FATTY_ACID_METABOLISM
G0S2	Absent	HALLMARK_FATTY_ACID_METABOLISM
GABARAPL1	Absent	HALLMARK_FATTY_ACID_METABOLISM
GAPDHS	Glycolysis Gluconeogenesis	HALLMARK_FATTY_ACID_METABOLISM

GSTZ1	Absent	HALLMARK_FATTY_ACID_METABOLISM
H2AZ1	Absent	HALLMARK_FATTY_ACID_METABOLISM
HCCS	Absent	HALLMARK_FATTY_ACID_METABOLISM
HPGD	Absent	HALLMARK_FATTY_ACID_METABOLISM
HSD17B10	Absent	HALLMARK_FATTY_ACID_METABOLISM
HSD17B11	Absent	HALLMARK_FATTY_ACID_METABOLISM
HSDL2	Absent	HALLMARK_FATTY_ACID_METABOLISM
HSP90AA1	Absent	HALLMARK_FATTY_ACID_METABOLISM
HSPH1	Absent	HALLMARK_FATTY_ACID_METABOLISM
IDH1	Citrate cycle TCA cycle	HALLMARK_FATTY_ACID_METABOLISM
IDH3B	Citrate cycle TCA cycle	HALLMARK_FATTY_ACID_METABOLISM
IDH3G	Citrate cycle TCA cycle	HALLMARK_FATTY_ACID_METABOLISM
IDI1	Absent	HALLMARK_FATTY_ACID_METABOLISM
LGALS1	Absent	HALLMARK_FATTY_ACID_METABOLISM
METAP1	Absent	HALLMARK_FATTY_ACID_METABOLISM
MIX23	Absent	HALLMARK_FATTY_ACID_METABOLISM
NBN	Absent	HALLMARK_FATTY_ACID_METABOLISM
NCAPH2	Absent	HALLMARK_FATTY_ACID_METABOLISM
NTHL1	Absent	HALLMARK_FATTY_ACID_METABOLISM
OSTC	Absent	HALLMARK_FATTY_ACID_METABOLISM
PCBD1	Absent	HALLMARK_FATTY_ACID_METABOLISM
PPARA	Absent	HALLMARK_FATTY_ACID_METABOLISM
PRDX6	Absent	HALLMARK_FATTY_ACID_METABOLISM
PSME1	Absent	HALLMARK_FATTY_ACID_METABOLISM
PTPRG	Absent	HALLMARK_FATTY_ACID_METABOLISM
PTS	Absent	HALLMARK_FATTY_ACID_METABOLISM
RAP1GDS1	Absent	HALLMARK_FATTY_ACID_METABOLISM
RDH11	Absent	HALLMARK_FATTY_ACID_METABOLISM
RDH16	Absent	HALLMARK_FATTY_ACID_METABOLISM
REEP6	Absent	HALLMARK_FATTY_ACID_METABOLISM
RETSAT	Absent	HALLMARK_FATTY_ACID_METABOLISM
S100A10	Absent	HALLMARK_FATTY_ACID_METABOLISM
SERINC1	Absent	HALLMARK_FATTY_ACID_METABOLISM
SLC22A5	Absent	HALLMARK_FATTY_ACID_METABOLISM
TP53INP2	Absent	HALLMARK_FATTY_ACID_METABOLISM
UBE2L6	Absent	HALLMARK_FATTY_ACID_METABOLISM
UROD	Absent	HALLMARK_FATTY_ACID_METABOLISM
UROS	Absent	HALLMARK_FATTY_ACID_METABOLISM
VNN1	Absent	HALLMARK_FATTY_ACID_METABOLISM
XIST	Absent	HALLMARK_FATTY_ACID_METABOLISM
YWHAH	Absent	HALLMARK_FATTY_ACID_METABOLISM
ACAD8	Absent	REACTOME_METABOLISM_OF_AMINO_ACIDS_AND_DERIVATIVES
ADO	Absent	REACTOME_METABOLISM_OF_AMINO_ACIDS_AND_DERIVATIVES
AIMP1	Absent	REACTOME_METABOLISM_OF_AMINO_ACIDS_AND_DERIVATIVES
AIMP2	Absent	REACTOME_METABOLISM_OF_AMINO_ACIDS_AND_DERIVATIVES
ASPG	Absent	REACTOME_METABOLISM_OF_AMINO_ACIDS_AND_DERIVATIVES
AZIN1	Absent	REACTOME_METABOLISM_OF_AMINO_ACIDS_AND_DERIVATIVES
BCKDK	Absent	REACTOME_METABOLISM_OF_AMINO_ACIDS_AND_DERIVATIVES
CGA	Absent	REACTOME_METABOLISM_OF_LIPIDS
CRYM	Absent	REACTOME_METABOLISM_OF_AMINO_ACIDS_AND_DERIVATIVES
CSAD	Absent	REACTOME_METABOLISM_OF_AMINO_ACIDS_AND_DERIVATIVES
DARS1	Absent	REACTOME_METABOLISM_OF_AMINO_ACIDS_AND_DERIVATIVES
DBH	Absent	REACTOME_METABOLISM_OF_AMINO_ACIDS_AND_DERIVATIVES
DCT	Absent	REACTOME_METABOLISM_OF_AMINO_ACIDS_AND_DERIVATIVES
DIO1	Absent	REACTOME_METABOLISM_OF_AMINO_ACIDS_AND_DERIVATIVES
DIO2	Absent	REACTOME_METABOLISM_OF_AMINO_ACIDS_AND_DERIVATIVES
DIO3	Absent	REACTOME_METABOLISM_OF_AMINO_ACIDS_AND_DERIVATIVES
DUOX1	Absent	REACTOME_METABOLISM_OF_AMINO_ACIDS_AND_DERIVATIVES

DUOX2	Absent	REACTOME_METABOLISM_OF_AMINO_ACIDS_AND_DERIVATIVES
EEF1E1	Absent	REACTOME_METABOLISM_OF_AMINO_ACIDS_AND_DERIVATIVES
EEFSEC	Absent	REACTOME_METABOLISM_OF_AMINO_ACIDS_AND_DERIVATIVES
EPRS1	Absent	REACTOME_METABOLISM_OF_AMINO_ACIDS_AND_DERIVATIVES
FAH	Absent	REACTOME_METABOLISM_OF_AMINO_ACIDS_AND_DERIVATIVES
FAU	Absent	REACTOME_METABOLISM_OF_AMINO_ACIDS_AND_DERIVATIVES
GADL1	Absent	REACTOME_METABOLISM_OF_AMINO_ACIDS_AND_DERIVATIVES
GSR	Absent	REACTOME_METABOLISM_OF_AMINO_ACIDS_AND_DERIVATIVES
HGD	Absent	REACTOME_METABOLISM_OF_AMINO_ACIDS_AND_DERIVATIVES
HIBADH	Absent	REACTOME_METABOLISM_OF_AMINO_ACIDS_AND_DERIVATIVES
IARS1	Absent	REACTOME_METABOLISM_OF_AMINO_ACIDS_AND_DERIVATIVES
IVD	Absent	REACTOME_METABOLISM_OF_AMINO_ACIDS_AND_DERIVATIVES
IYD	Absent	REACTOME_METABOLISM_OF_AMINO_ACIDS_AND_DERIVATIVES
KARS1	Absent	REACTOME_METABOLISM_OF_AMINO_ACIDS_AND_DERIVATIVES
LARS1	Absent	REACTOME_METABOLISM_OF_AMINO_ACIDS_AND_DERIVATIVES
LIAS	Absent	REACTOME_METABOLISM_OF_AMINO_ACIDS_AND_DERIVATIVES
LIPT1	Absent	REACTOME_METABOLISM_OF_AMINO_ACIDS_AND_DERIVATIVES
MARS1	Absent	REACTOME_METABOLISM_OF_AMINO_ACIDS_AND_DERIVATIVES
MCCC1	Absent	REACTOME_METABOLISM_OF_AMINO_ACIDS_AND_DERIVATIVES
MCCC2	Absent	REACTOME_METABOLISM_OF_AMINO_ACIDS_AND_DERIVATIVES
MTRR	Absent	REACTOME_METABOLISM_OF_AMINO_ACIDS_AND_DERIVATIVES
NAALAD2	Absent	REACTOME_METABOLISM_OF_AMINO_ACIDS_AND_DERIVATIVES
NAGS	Absent	REACTOME_METABOLISM_OF_AMINO_ACIDS_AND_DERIVATIVES
NMRAL1	Absent	REACTOME_METABOLISM_OF_AMINO_ACIDS_AND_DERIVATIVES
NNMT	Absent	REACTOME_METABOLISM_OF_AMINO_ACIDS_AND_DERIVATIVES
NQO1	Absent	REACTOME_METABOLISM_OF_AMINO_ACIDS_AND_DERIVATIVES
OAZ1	Absent	REACTOME_METABOLISM_OF_AMINO_ACIDS_AND_DERIVATIVES
OAZ2	Absent	REACTOME_METABOLISM_OF_AMINO_ACIDS_AND_DERIVATIVES
OAZ3	Absent	REACTOME_METABOLISM_OF_AMINO_ACIDS_AND_DERIVATIVES
OCA2	Absent	REACTOME_METABOLISM_OF_AMINO_ACIDS_AND_DERIVATIVES
OGDH	Citrate cycle TCA cycle	REACTOME_METABOLISM_OF_AMINO_ACIDS_AND_DERIVATIVES
OTC	Absent	REACTOME_METABOLISM_OF_AMINO_ACIDS_AND_DERIVATIVES
PAOX	Absent	REACTOME_METABOLISM_OF_AMINO_ACIDS_AND_DERIVATIVES
PDHX	Absent	REACTOME_METABOLISM_OF_AMINO_ACIDS_AND_DERIVATIVES
PNMT	Absent	REACTOME_METABOLISM_OF_AMINO_ACIDS_AND_DERIVATIVES
PPM1K	Absent	REACTOME_METABOLISM_OF_AMINO_ACIDS_AND_DERIVATIVES
PRODH2	Absent	REACTOME_METABOLISM_OF_AMINO_ACIDS_AND_DERIVATIVES
PSMA1	Absent	REACTOME_METABOLISM_OF_AMINO_ACIDS_AND_DERIVATIVES
PSMA2	Absent	REACTOME_METABOLISM_OF_AMINO_ACIDS_AND_DERIVATIVES
PSMA3	Absent	REACTOME_METABOLISM_OF_AMINO_ACIDS_AND_DERIVATIVES
PSMA4	Absent	REACTOME_METABOLISM_OF_AMINO_ACIDS_AND_DERIVATIVES
PSMA5	Absent	REACTOME_METABOLISM_OF_AMINO_ACIDS_AND_DERIVATIVES
PSMA6	Absent	REACTOME_METABOLISM_OF_AMINO_ACIDS_AND_DERIVATIVES
PSMA7	Absent	REACTOME_METABOLISM_OF_AMINO_ACIDS_AND_DERIVATIVES
PSMA8	Absent	REACTOME_METABOLISM_OF_AMINO_ACIDS_AND_DERIVATIVES
PSMB1	Absent	REACTOME_METABOLISM_OF_AMINO_ACIDS_AND_DERIVATIVES
PSMB10	Absent	REACTOME_METABOLISM_OF_AMINO_ACIDS_AND_DERIVATIVES
PSMB11	Absent	REACTOME_METABOLISM_OF_AMINO_ACIDS_AND_DERIVATIVES
PSMB2	Absent	REACTOME_METABOLISM_OF_AMINO_ACIDS_AND_DERIVATIVES
PSMB3	Absent	REACTOME_METABOLISM_OF_AMINO_ACIDS_AND_DERIVATIVES
PSMB4	Absent	REACTOME_METABOLISM_OF_AMINO_ACIDS_AND_DERIVATIVES
PSMB5	Absent	REACTOME_METABOLISM_OF_AMINO_ACIDS_AND_DERIVATIVES
PSMB6	Absent	REACTOME_METABOLISM_OF_AMINO_ACIDS_AND_DERIVATIVES
PSMB7	Absent	REACTOME_METABOLISM_OF_AMINO_ACIDS_AND_DERIVATIVES
PSMB8	Absent	REACTOME_METABOLISM_OF_AMINO_ACIDS_AND_DERIVATIVES
PSMB9	Absent	REACTOME_METABOLISM_OF_AMINO_ACIDS_AND_DERIVATIVES
PSMC1	Absent	REACTOME_METABOLISM_OF_AMINO_ACIDS_AND_DERIVATIVES
PSMC2	Absent	REACTOME_METABOLISM_OF_AMINO_ACIDS_AND_DERIVATIVES

PSMC3	Absent	REACTOME_METABOLISM_OF_AMINO_ACIDS_AND_DERIVATIVES
PSMC4	Absent	REACTOME_METABOLISM_OF_AMINO_ACIDS_AND_DERIVATIVES
PSMC5	Absent	REACTOME_METABOLISM_OF_AMINO_ACIDS_AND_DERIVATIVES
PSMC6	Absent	REACTOME_METABOLISM_OF_AMINO_ACIDS_AND_DERIVATIVES
PSMD1	Absent	REACTOME_METABOLISM_OF_AMINO_ACIDS_AND_DERIVATIVES
PSMD10	Absent	REACTOME_METABOLISM_OF_AMINO_ACIDS_AND_DERIVATIVES
PSMD11	Absent	REACTOME_METABOLISM_OF_AMINO_ACIDS_AND_DERIVATIVES
PSMD12	Absent	REACTOME_METABOLISM_OF_AMINO_ACIDS_AND_DERIVATIVES
PSMD13	Absent	REACTOME_METABOLISM_OF_AMINO_ACIDS_AND_DERIVATIVES
PSMD14	Absent	REACTOME_METABOLISM_OF_AMINO_ACIDS_AND_DERIVATIVES
PSMD2	Absent	REACTOME_METABOLISM_OF_AMINO_ACIDS_AND_DERIVATIVES
PSMD3	Absent	REACTOME_METABOLISM_OF_AMINO_ACIDS_AND_DERIVATIVES
PSMD4	Absent	REACTOME_METABOLISM_OF_AMINO_ACIDS_AND_DERIVATIVES
PSMD5	Absent	REACTOME_METABOLISM_OF_AMINO_ACIDS_AND_DERIVATIVES
PSMD6	Absent	REACTOME_METABOLISM_OF_AMINO_ACIDS_AND_DERIVATIVES
PSMD7	Absent	REACTOME_METABOLISM_OF_AMINO_ACIDS_AND_DERIVATIVES
PSMD8	Absent	REACTOME_METABOLISM_OF_AMINO_ACIDS_AND_DERIVATIVES
PSMD9	Absent	REACTOME_METABOLISM_OF_AMINO_ACIDS_AND_DERIVATIVES
PSME2	Absent	REACTOME_METABOLISM_OF_AMINO_ACIDS_AND_DERIVATIVES
PSME3	Absent	REACTOME_METABOLISM_OF_AMINO_ACIDS_AND_DERIVATIVES
PSME4	Absent	REACTOME_METABOLISM_OF_AMINO_ACIDS_AND_DERIVATIVES
PSMF1	Absent	REACTOME_METABOLISM_OF_AMINO_ACIDS_AND_DERIVATIVES
PSTK	Absent	REACTOME_METABOLISM_OF_AMINO_ACIDS_AND_DERIVATIVES
PXMP2	Absent	REACTOME_METABOLISM_OF_AMINO_ACIDS_AND_DERIVATIVES
QARS1	Absent	REACTOME_METABOLISM_OF_AMINO_ACIDS_AND_DERIVATIVES
QDPR	Absent	REACTOME_METABOLISM_OF_AMINO_ACIDS_AND_DERIVATIVES
RARS1	Absent	REACTOME_METABOLISM_OF_AMINO_ACIDS_AND_DERIVATIVES
RIDA	Absent	REACTOME_METABOLISM_OF_AMINO_ACIDS_AND_DERIVATIVES
RPL10	Absent	REACTOME_METABOLISM_OF_AMINO_ACIDS_AND_DERIVATIVES
RPL10A	Absent	REACTOME_METABOLISM_OF_AMINO_ACIDS_AND_DERIVATIVES
RPL10L	Absent	REACTOME_METABOLISM_OF_AMINO_ACIDS_AND_DERIVATIVES
RPL11	Absent	REACTOME_METABOLISM_OF_AMINO_ACIDS_AND_DERIVATIVES
RPL12	Absent	REACTOME_METABOLISM_OF_AMINO_ACIDS_AND_DERIVATIVES
RPL13	Absent	REACTOME_METABOLISM_OF_AMINO_ACIDS_AND_DERIVATIVES
RPL13A	Absent	REACTOME_METABOLISM_OF_AMINO_ACIDS_AND_DERIVATIVES
RPL14	Absent	REACTOME_METABOLISM_OF_AMINO_ACIDS_AND_DERIVATIVES
RPL15	Absent	REACTOME_METABOLISM_OF_AMINO_ACIDS_AND_DERIVATIVES
RPL17	Absent	REACTOME_METABOLISM_OF_AMINO_ACIDS_AND_DERIVATIVES
RPL18	Absent	REACTOME_METABOLISM_OF_AMINO_ACIDS_AND_DERIVATIVES
RPL18A	Absent	REACTOME_METABOLISM_OF_AMINO_ACIDS_AND_DERIVATIVES
RPL19	Absent	REACTOME_METABOLISM_OF_AMINO_ACIDS_AND_DERIVATIVES
RPL21	Absent	REACTOME_METABOLISM_OF_AMINO_ACIDS_AND_DERIVATIVES
RPL22	Absent	REACTOME_METABOLISM_OF_AMINO_ACIDS_AND_DERIVATIVES
RPL22L1	Absent	REACTOME_METABOLISM_OF_AMINO_ACIDS_AND_DERIVATIVES
RPL23	Absent	REACTOME_METABOLISM_OF_AMINO_ACIDS_AND_DERIVATIVES
RPL23A	Absent	REACTOME_METABOLISM_OF_AMINO_ACIDS_AND_DERIVATIVES
RPL24	Absent	REACTOME_METABOLISM_OF_AMINO_ACIDS_AND_DERIVATIVES
RPL26	Absent	REACTOME_METABOLISM_OF_AMINO_ACIDS_AND_DERIVATIVES
RPL26L1	Absent	REACTOME_METABOLISM_OF_AMINO_ACIDS_AND_DERIVATIVES
RPL27	Absent	REACTOME_METABOLISM_OF_AMINO_ACIDS_AND_DERIVATIVES
RPL27A	Absent	REACTOME_METABOLISM_OF_AMINO_ACIDS_AND_DERIVATIVES
RPL28	Absent	REACTOME_METABOLISM_OF_AMINO_ACIDS_AND_DERIVATIVES
RPL29	Absent	REACTOME_METABOLISM_OF_AMINO_ACIDS_AND_DERIVATIVES
RPL3	Absent	REACTOME_METABOLISM_OF_AMINO_ACIDS_AND_DERIVATIVES
RPL30	Absent	REACTOME_METABOLISM_OF_AMINO_ACIDS_AND_DERIVATIVES
RPL31	Absent	REACTOME_METABOLISM_OF_AMINO_ACIDS_AND_DERIVATIVES
RPL32	Absent	REACTOME_METABOLISM_OF_AMINO_ACIDS_AND_DERIVATIVES
RPL34	Absent	REACTOME_METABOLISM_OF_AMINO_ACIDS_AND_DERIVATIVES

RPL35	Absent	REACTOME_METABOLISM_OF_AMINO_ACIDS_AND_DERIVATIVES
RPL35A	Absent	REACTOME_METABOLISM_OF_AMINO_ACIDS_AND_DERIVATIVES
RPL36	Absent	REACTOME_METABOLISM_OF_AMINO_ACIDS_AND_DERIVATIVES
RPL36A	Absent	REACTOME_METABOLISM_OF_AMINO_ACIDS_AND_DERIVATIVES
RPL36AL	Absent	REACTOME_METABOLISM_OF_AMINO_ACIDS_AND_DERIVATIVES
RPL37	Absent	REACTOME_METABOLISM_OF_AMINO_ACIDS_AND_DERIVATIVES
RPL37A	Absent	REACTOME_METABOLISM_OF_AMINO_ACIDS_AND_DERIVATIVES
RPL38	Absent	REACTOME_METABOLISM_OF_AMINO_ACIDS_AND_DERIVATIVES
RPL39	Absent	REACTOME_METABOLISM_OF_AMINO_ACIDS_AND_DERIVATIVES
RPL39L	Absent	REACTOME_METABOLISM_OF_AMINO_ACIDS_AND_DERIVATIVES
RPL3L	Absent	REACTOME_METABOLISM_OF_AMINO_ACIDS_AND_DERIVATIVES
RPL4	Absent	REACTOME_METABOLISM_OF_AMINO_ACIDS_AND_DERIVATIVES
RPL41	Absent	REACTOME_METABOLISM_OF_AMINO_ACIDS_AND_DERIVATIVES
RPL5	Absent	REACTOME_METABOLISM_OF_AMINO_ACIDS_AND_DERIVATIVES
RPL6	Absent	REACTOME_METABOLISM_OF_AMINO_ACIDS_AND_DERIVATIVES
RPL7	Absent	REACTOME_METABOLISM_OF_AMINO_ACIDS_AND_DERIVATIVES
RPL7A	Absent	REACTOME_METABOLISM_OF_AMINO_ACIDS_AND_DERIVATIVES
RPL8	Absent	REACTOME_METABOLISM_OF_AMINO_ACIDS_AND_DERIVATIVES
RPL9	Absent	REACTOME_METABOLISM_OF_AMINO_ACIDS_AND_DERIVATIVES
RPLP0	Absent	REACTOME_METABOLISM_OF_AMINO_ACIDS_AND_DERIVATIVES
RPLP1	Absent	REACTOME_METABOLISM_OF_AMINO_ACIDS_AND_DERIVATIVES
RPLP2	Absent	REACTOME_METABOLISM_OF_AMINO_ACIDS_AND_DERIVATIVES
RPS10	Absent	REACTOME_METABOLISM_OF_AMINO_ACIDS_AND_DERIVATIVES
RPS11	Absent	REACTOME_METABOLISM_OF_AMINO_ACIDS_AND_DERIVATIVES
RPS12	Absent	REACTOME_METABOLISM_OF_AMINO_ACIDS_AND_DERIVATIVES
RPS13	Absent	REACTOME_METABOLISM_OF_AMINO_ACIDS_AND_DERIVATIVES
RPS14	Absent	REACTOME_METABOLISM_OF_AMINO_ACIDS_AND_DERIVATIVES
RPS15	Absent	REACTOME_METABOLISM_OF_AMINO_ACIDS_AND_DERIVATIVES
RPS15A	Absent	REACTOME_METABOLISM_OF_AMINO_ACIDS_AND_DERIVATIVES
RPS16	Absent	REACTOME_METABOLISM_OF_AMINO_ACIDS_AND_DERIVATIVES
RPS17	Absent	REACTOME_METABOLISM_OF_AMINO_ACIDS_AND_DERIVATIVES
RPS18	Absent	REACTOME_METABOLISM_OF_AMINO_ACIDS_AND_DERIVATIVES
RPS19	Absent	REACTOME_METABOLISM_OF_AMINO_ACIDS_AND_DERIVATIVES
RPS2	Absent	REACTOME_METABOLISM_OF_AMINO_ACIDS_AND_DERIVATIVES
RPS20	Absent	REACTOME_METABOLISM_OF_AMINO_ACIDS_AND_DERIVATIVES
RPS21	Absent	REACTOME_METABOLISM_OF_AMINO_ACIDS_AND_DERIVATIVES
RPS23	Absent	REACTOME_METABOLISM_OF_AMINO_ACIDS_AND_DERIVATIVES
RPS24	Absent	REACTOME_METABOLISM_OF_AMINO_ACIDS_AND_DERIVATIVES
RPS25	Absent	REACTOME_METABOLISM_OF_AMINO_ACIDS_AND_DERIVATIVES
RPS26	Absent	REACTOME_METABOLISM_OF_AMINO_ACIDS_AND_DERIVATIVES
RPS27	Absent	REACTOME_METABOLISM_OF_AMINO_ACIDS_AND_DERIVATIVES
RPS27A	Absent	REACTOME_METABOLISM_OF_CARBOHYDRATES
RPS27L	Absent	REACTOME_METABOLISM_OF_AMINO_ACIDS_AND_DERIVATIVES
RPS28	Absent	REACTOME_METABOLISM_OF_AMINO_ACIDS_AND_DERIVATIVES
RPS29	Absent	REACTOME_METABOLISM_OF_AMINO_ACIDS_AND_DERIVATIVES
RPS3	Absent	REACTOME_METABOLISM_OF_AMINO_ACIDS_AND_DERIVATIVES
RPS3A	Absent	REACTOME_METABOLISM_OF_AMINO_ACIDS_AND_DERIVATIVES
RPS4X	Absent	REACTOME_METABOLISM_OF_AMINO_ACIDS_AND_DERIVATIVES
RPS4Y1	Absent	REACTOME_METABOLISM_OF_AMINO_ACIDS_AND_DERIVATIVES
RPS4Y2	Absent	REACTOME_METABOLISM_OF_AMINO_ACIDS_AND_DERIVATIVES
RPS5	Absent	REACTOME_METABOLISM_OF_AMINO_ACIDS_AND_DERIVATIVES
RPS6	Absent	REACTOME_METABOLISM_OF_AMINO_ACIDS_AND_DERIVATIVES
RPS7	Absent	REACTOME_METABOLISM_OF_AMINO_ACIDS_AND_DERIVATIVES
RPS8	Absent	REACTOME_METABOLISM_OF_AMINO_ACIDS_AND_DERIVATIVES
RPS9	Absent	REACTOME_METABOLISM_OF_AMINO_ACIDS_AND_DERIVATIVES
RPSA	Absent	REACTOME_METABOLISM_OF_AMINO_ACIDS_AND_DERIVATIVES
SARS1	Absent	REACTOME_METABOLISM_OF_AMINO_ACIDS_AND_DERIVATIVES
SCLY	Absent	REACTOME_METABOLISM_OF_AMINO_ACIDS_AND_DERIVATIVES

SECISBP2	Absent	REACTOME_METABOLISM_OF_AMINO_ACIDS_AND_DERIVATIVES
SEM1	Absent	REACTOME_METABOLISM_OF_AMINO_ACIDS_AND_DERIVATIVES
SEPHS2	Absent	REACTOME_METABOLISM_OF_AMINO_ACIDS_AND_DERIVATIVES
SEPSECS	Absent	REACTOME_METABOLISM_OF_AMINO_ACIDS_AND_DERIVATIVES
SERINC2	Absent	REACTOME_METABOLISM_OF_AMINO_ACIDS_AND_DERIVATIVES
SERINC3	Absent	REACTOME_METABOLISM_OF_AMINO_ACIDS_AND_DERIVATIVES
SERINC4	Absent	REACTOME_METABOLISM_OF_AMINO_ACIDS_AND_DERIVATIVES
SERINC5	Absent	REACTOME_METABOLISM_OF_AMINO_ACIDS_AND_DERIVATIVES
SLC25A10	Absent	REACTOME_METABOLISM_OF_CARBOHYDRATES
SLC25A12	Absent	REACTOME_METABOLISM_OF_CARBOHYDRATES
SLC25A13	Absent	REACTOME_METABOLISM_OF_CARBOHYDRATES
SLC25A15	Absent	REACTOME_METABOLISM_OF_AMINO_ACIDS_AND_DERIVATIVES
SLC25A2	Absent	REACTOME_METABOLISM_OF_AMINO_ACIDS_AND_DERIVATIVES
SLC25A21	Absent	REACTOME_METABOLISM_OF_AMINO_ACIDS_AND_DERIVATIVES
SLC25A44	Absent	REACTOME_METABOLISM_OF_AMINO_ACIDS_AND_DERIVATIVES
SLC36A4	Absent	REACTOME_METABOLISM_OF_AMINO_ACIDS_AND_DERIVATIVES
SLC3A2	Absent	REACTOME_METABOLISM_OF_AMINO_ACIDS_AND_DERIVATIVES
SLC44A1	Absent	REACTOME_PHOSPHOLIPID_METABOLISM
SLC45A2	Absent	REACTOME_METABOLISM_OF_AMINO_ACIDS_AND_DERIVATIVES
SLC5A5	Absent	REACTOME_METABOLISM_OF_AMINO_ACIDS_AND_DERIVATIVES
SLC6A11	Absent	REACTOME_METABOLISM_OF_AMINO_ACIDS_AND_DERIVATIVES
SLC6A12	Absent	REACTOME_METABOLISM_OF_AMINO_ACIDS_AND_DERIVATIVES
SLC6A7	Absent	REACTOME_METABOLISM_OF_AMINO_ACIDS_AND_DERIVATIVES
SLC6A8	Absent	REACTOME_METABOLISM_OF_AMINO_ACIDS_AND_DERIVATIVES
SLC7A5	Absent	REACTOME_METABOLISM_OF_AMINO_ACIDS_AND_DERIVATIVES
TH	Absent	REACTOME_METABOLISM_OF_AMINO_ACIDS_AND_DERIVATIVES
TPO	Absent	REACTOME_METABOLISM_OF_AMINO_ACIDS_AND_DERIVATIVES
TSHB	Absent	REACTOME_METABOLISM_OF_AMINO_ACIDS_AND_DERIVATIVES
TSTD1	Absent	REACTOME_METABOLISM_OF_AMINO_ACIDS_AND_DERIVATIVES
TXN2	Absent	REACTOME_METABOLISM_OF_AMINO_ACIDS_AND_DERIVATIVES
TXNRD1	Absent	REACTOME_METABOLISM_OF_LIPIDS
TYR	Absent	REACTOME_METABOLISM_OF_AMINO_ACIDS_AND_DERIVATIVES
TYRP1	Absent	REACTOME_METABOLISM_OF_AMINO_ACIDS_AND_DERIVATIVES
UBA52	Absent	REACTOME_METABOLISM_OF_CARBOHYDRATES
AAAS	Absent	REACTOME_METABOLISM_OF_CARBOHYDRATES
ABCC5	Absent	REACTOME_METABOLISM_OF_CARBOHYDRATES
ACAN	Absent	REACTOME_METABOLISM_OF_CARBOHYDRATES
ADPGK	Glycolysis Gluconeogenesis	REACTOME_METABOLISM_OF_CARBOHYDRATES
AGRN	Absent	REACTOME_METABOLISM_OF_CARBOHYDRATES
ALDOB	Fructose and mannose metabolism	REACTOME_METABOLISM_OF_CARBOHYDRATES
ALDOC	Fructose and mannose metabolism	REACTOME_METABOLISM_OF_CARBOHYDRATES
B4GALNT2	Absent	REACTOME_METABOLISM_OF_CARBOHYDRATES
BCAN	Absent	REACTOME_METABOLISM_OF_CARBOHYDRATES
BGN	Absent	REACTOME_METABOLISM_OF_CARBOHYDRATES
CALM1	Absent	REACTOME_METABOLISM_OF_CARBOHYDRATES
CD44	Absent	REACTOME_METABOLISM_OF_CARBOHYDRATES
CEMIP	Absent	REACTOME_METABOLISM_OF_CARBOHYDRATES
CHP1	Absent	REACTOME_METABOLISM_OF_CARBOHYDRATES
CHST5	Absent	REACTOME_METABOLISM_OF_CARBOHYDRATES
CRYL1	Pentose and glucuronate interconversions	REACTOME_METABOLISM_OF_CARBOHYDRATES
CSPG4	Absent	REACTOME_METABOLISM_OF_CARBOHYDRATES
CSPG5	Absent	REACTOME_METABOLISM_OF_CARBOHYDRATES
DCN	Absent	REACTOME_METABOLISM_OF_CARBOHYDRATES
DCXR	Pentose and glucuronate interconversions	REACTOME_METABOLISM_OF_CARBOHYDRATES
DERA	Pentose phosphate pathway	REACTOME_METABOLISM_OF_CARBOHYDRATES
DSEL	Absent	REACTOME_METABOLISM_OF_CARBOHYDRATES
ENO1	Glycolysis Gluconeogenesis	REACTOME_METABOLISM_OF_CARBOHYDRATES
EPM2A	Absent	REACTOME_METABOLISM_OF_CARBOHYDRATES

FBP1	Fructose and mannose metabolism	REACTOME_METABOLISM_OF_CARBOHYDRATES
FBP2	Fructose and mannose metabolism	REACTOME_METABOLISM_OF_CARBOHYDRATES
FMOD	Absent	REACTOME_METABOLISM_OF_CARBOHYDRATES
FUT10	Absent	REACTOME_METABOLISM_OF_CARBOHYDRATES
FUT11	Absent	REACTOME_METABOLISM_OF_CARBOHYDRATES
G6PD	Pentose phosphate pathway	REACTOME_METABOLISM_OF_CARBOHYDRATES
GAPDH	Glycolysis Gluconeogenesis	REACTOME_METABOLISM_OF_CARBOHYDRATES
GCKR	Absent	REACTOME_METABOLISM_OF_CARBOHYDRATES
GLB1L	Absent	REACTOME_METABOLISM_OF_LIPIDS
GPC1	Absent	REACTOME_METABOLISM_OF_CARBOHYDRATES
GPC2	Absent	REACTOME_METABOLISM_OF_CARBOHYDRATES
GPC3	Absent	REACTOME_METABOLISM_OF_CARBOHYDRATES
GPC4	Absent	REACTOME_METABOLISM_OF_CARBOHYDRATES
GPC5	Absent	REACTOME_METABOLISM_OF_CARBOHYDRATES
GPC6	Absent	REACTOME_METABOLISM_OF_CARBOHYDRATES
HAS1	Absent	REACTOME_METABOLISM_OF_CARBOHYDRATES
HAS2	Absent	REACTOME_METABOLISM_OF_CARBOHYDRATES
HAS3	Absent	REACTOME_METABOLISM_OF_CARBOHYDRATES
HMMR	Absent	REACTOME_METABOLISM_OF_CARBOHYDRATES
HS3ST4	Absent	REACTOME_METABOLISM_OF_CARBOHYDRATES
HS3ST6	Absent	REACTOME_METABOLISM_OF_CARBOHYDRATES
HSPG2	Absent	REACTOME_METABOLISM_OF_CARBOHYDRATES
KERA	Absent	REACTOME_METABOLISM_OF_CARBOHYDRATES
KHK	Fructose and mannose metabolism	REACTOME_METABOLISM_OF_CARBOHYDRATES
LUM	Absent	REACTOME_METABOLISM_OF_CARBOHYDRATES
LYVE1	Absent	REACTOME_METABOLISM_OF_CARBOHYDRATES
NCAN	Absent	REACTOME_METABOLISM_OF_CARBOHYDRATES
NDC1	Absent	REACTOME_METABOLISM_OF_CARBOHYDRATES
NHLRC1	Absent	REACTOME_METABOLISM_OF_CARBOHYDRATES
NUP107	Absent	REACTOME_METABOLISM_OF_CARBOHYDRATES
NUP133	Absent	REACTOME_METABOLISM_OF_CARBOHYDRATES
NUP153	Absent	REACTOME_METABOLISM_OF_CARBOHYDRATES
NUP155	Absent	REACTOME_METABOLISM_OF_CARBOHYDRATES
NUP160	Absent	REACTOME_METABOLISM_OF_CARBOHYDRATES
NUP188	Absent	REACTOME_METABOLISM_OF_CARBOHYDRATES
NUP205	Absent	REACTOME_METABOLISM_OF_CARBOHYDRATES
NUP210	Absent	REACTOME_METABOLISM_OF_CARBOHYDRATES
NUP214	Absent	REACTOME_METABOLISM_OF_CARBOHYDRATES
NUP35	Absent	REACTOME_METABOLISM_OF_CARBOHYDRATES
NUP37	Absent	REACTOME_METABOLISM_OF_CARBOHYDRATES
NUP42	Absent	REACTOME_METABOLISM_OF_CARBOHYDRATES
NUP43	Absent	REACTOME_METABOLISM_OF_CARBOHYDRATES
NUP50	Absent	REACTOME_METABOLISM_OF_CARBOHYDRATES
NUP54	Absent	REACTOME_METABOLISM_OF_CARBOHYDRATES
NUP58	Absent	REACTOME_METABOLISM_OF_CARBOHYDRATES
NUP62	Absent	REACTOME_METABOLISM_OF_CARBOHYDRATES
NUP85	Absent	REACTOME_METABOLISM_OF_CARBOHYDRATES
NUP88	Absent	REACTOME_METABOLISM_OF_CARBOHYDRATES
NUP93	Absent	REACTOME_METABOLISM_OF_CARBOHYDRATES
NUP98	Absent	REACTOME_METABOLISM_OF_CARBOHYDRATES
OGN	Absent	REACTOME_METABOLISM_OF_CARBOHYDRATES
OMD	Absent	REACTOME_METABOLISM_OF_CARBOHYDRATES
PFKFB1	Fructose and mannose metabolism	REACTOME_METABOLISM_OF_CARBOHYDRATES
PFKFB2	Fructose and mannose metabolism	REACTOME_METABOLISM_OF_CARBOHYDRATES
PFKFB3	Fructose and mannose metabolism	REACTOME_METABOLISM_OF_CARBOHYDRATES
PFKFB4	Fructose and mannose metabolism	REACTOME_METABOLISM_OF_CARBOHYDRATES
PGD	Pentose phosphate pathway	REACTOME_METABOLISM_OF_CARBOHYDRATES
PGK1	Glycolysis Gluconeogenesis	REACTOME_METABOLISM_OF_CARBOHYDRATES

PGK2	Glycolysis Gluconeogenesis	REACTOME_METABOLISM_OF_CARBOHYDRATES
PGLS	Pentose phosphate pathway	REACTOME_METABOLISM_OF_CARBOHYDRATES
PHKA1	Absent	REACTOME_METABOLISM_OF_CARBOHYDRATES
PHKA2	Absent	REACTOME_METABOLISM_OF_CARBOHYDRATES
PHKB	Absent	REACTOME_METABOLISM_OF_CARBOHYDRATES
PHKG1	Absent	REACTOME_METABOLISM_OF_CARBOHYDRATES
PHKG2	Absent	REACTOME_METABOLISM_OF_CARBOHYDRATES
POM121	Absent	REACTOME_METABOLISM_OF_CARBOHYDRATES
POM121C	Absent	REACTOME_METABOLISM_OF_CARBOHYDRATES
PPP1R3C	Absent	REACTOME_METABOLISM_OF_CARBOHYDRATES
PPP2CA	Absent	REACTOME_METABOLISM_OF_CARBOHYDRATES
PPP2CB	Absent	REACTOME_METABOLISM_OF_CARBOHYDRATES
PPP2R1A	Absent	REACTOME_METABOLISM_OF_CARBOHYDRATES
PPP2R1B	Absent	REACTOME_METABOLISM_OF_CARBOHYDRATES
PPP2R5D	Absent	REACTOME_METABOLISM_OF_CARBOHYDRATES
PRELP	Absent	REACTOME_METABOLISM_OF_CARBOHYDRATES
PRKACA	Absent	REACTOME_METABOLISM_OF_LIPIDS
PRKACB	Absent	REACTOME_METABOLISM_OF_LIPIDS
PRKACG	Absent	REACTOME_METABOLISM_OF_LIPIDS
RAE1	Absent	REACTOME_METABOLISM_OF_CARBOHYDRATES
RANBP2	Absent	REACTOME_METABOLISM_OF_CARBOHYDRATES
RBKS	Pentose phosphate pathway	REACTOME_METABOLISM_OF_CARBOHYDRATES
RHCE	Absent	REACTOME_METABOLISM_OF_CARBOHYDRATES
RHD	Absent	REACTOME_METABOLISM_OF_CARBOHYDRATES
RPE	Pentose and glucuronate interconversions	REACTOME_METABOLISM_OF_CARBOHYDRATES
RPEL1	Pentose and glucuronate interconversions	REACTOME_METABOLISM_OF_CARBOHYDRATES
RPIA	Pentose phosphate pathway	REACTOME_METABOLISM_OF_CARBOHYDRATES
SDC1	Absent	REACTOME_METABOLISM_OF_CARBOHYDRATES
SDC2	Absent	REACTOME_METABOLISM_OF_CARBOHYDRATES
SDC3	Absent	REACTOME_METABOLISM_OF_CARBOHYDRATES
SDC4	Absent	REACTOME_METABOLISM_OF_CARBOHYDRATES
SEC13	Absent	REACTOME_METABOLISM_OF_CARBOHYDRATES
SEH1L	Absent	REACTOME_METABOLISM_OF_CARBOHYDRATES
SHPK	Absent	REACTOME_METABOLISM_OF_CARBOHYDRATES
SLC25A1	Absent	REACTOME_METABOLISM_OF_LIPIDS
SLC25A11	Absent	REACTOME_METABOLISM_OF_CARBOHYDRATES
SLC26A1	Absent	REACTOME_METABOLISM_OF_CARBOHYDRATES
SLC26A2	Absent	REACTOME_METABOLISM_OF_CARBOHYDRATES
SLC2A1	Absent	REACTOME_METABOLISM_OF_CARBOHYDRATES
SLC35B2	Absent	REACTOME_METABOLISM_OF_CARBOHYDRATES
SLC35B3	Absent	REACTOME_METABOLISM_OF_CARBOHYDRATES
SLC35D2	Absent	REACTOME_METABOLISM_OF_CARBOHYDRATES
SLC37A1	Absent	REACTOME_METABOLISM_OF_CARBOHYDRATES
SLC37A2	Absent	REACTOME_METABOLISM_OF_CARBOHYDRATES
SLC37A4	Absent	REACTOME_METABOLISM_OF_CARBOHYDRATES
SLC9A1	Absent	REACTOME_METABOLISM_OF_CARBOHYDRATES
SORD	Fructose and mannose metabolism	REACTOME_METABOLISM_OF_CARBOHYDRATES
STAB2	Absent	REACTOME_METABOLISM_OF_CARBOHYDRATES
TALDO1	Pentose phosphate pathway	REACTOME_METABOLISM_OF_CARBOHYDRATES
TKT	Pentose phosphate pathway	REACTOME_METABOLISM_OF_CARBOHYDRATES
TPR	Absent	REACTOME_METABOLISM_OF_CARBOHYDRATES
UBB	Absent	REACTOME_METABOLISM_OF_CARBOHYDRATES
UBC	Absent	REACTOME_METABOLISM_OF_CARBOHYDRATES
VCAN	Absent	REACTOME_METABOLISM_OF_CARBOHYDRATES
XYLB	Pentose and glucuronate interconversions	REACTOME_METABOLISM_OF_CARBOHYDRATES
ABCA1	Absent	REACTOME_METABOLISM_OF_LIPIDS
ABCB11	Absent	REACTOME_METABOLISM_OF_LIPIDS
ABCB4	Absent	REACTOME_METABOLISM_OF_LIPIDS

ABCC1	Absent	REACTOME_METABOLISM_OF_LIPIDS
ABCC3	Absent	REACTOME_METABOLISM_OF_LIPIDS
ABCD1	Absent	REACTOME_METABOLISM_OF_LIPIDS
ABHD3	Absent	REACTOME_PHOSPHOLIPID_METABOLISM
ABHD4	Absent	REACTOME_PHOSPHOLIPID_METABOLISM
ABHD5	Absent	REACTOME_METABOLISM_OF_LIPIDS
ACAD10	Absent	REACTOME_METABOLISM_OF_LIPIDS
ACAD11	Absent	REACTOME_METABOLISM_OF_LIPIDS
ACBD4	Absent	REACTOME_METABOLISM_OF_LIPIDS
ACBD5	Absent	REACTOME_METABOLISM_OF_LIPIDS
ACBD6	Absent	REACTOME_METABOLISM_OF_LIPIDS
ACBD7	Absent	REACTOME_METABOLISM_OF_LIPIDS
ACLY	Citrate cycle TCA cycle	REACTOME_METABOLISM_OF_LIPIDS
ACOT11	Absent	REACTOME_METABOLISM_OF_LIPIDS
ACOT13	Absent	REACTOME_METABOLISM_OF_LIPIDS
ACOT6	Absent	REACTOME_METABOLISM_OF_LIPIDS
ACOT9	Absent	REACTOME_METABOLISM_OF_LIPIDS
ACOXL	Absent	REACTOME_METABOLISM_OF_LIPIDS
ACP6	Absent	REACTOME_PHOSPHOLIPID_METABOLISM
ACSF2	Absent	REACTOME_METABOLISM_OF_LIPIDS
AGMO	Absent	REACTOME_METABOLISM_OF_LIPIDS
AGT	Absent	REACTOME_METABOLISM_OF_LIPIDS
AHR	Absent	REACTOME_METABOLISM_OF_LIPIDS
AHRR	Absent	REACTOME_METABOLISM_OF_LIPIDS
AKR1B15	Absent	REACTOME_METABOLISM_OF_LIPIDS
ALB	Absent	REACTOME_METABOLISM_OF_LIPIDS
ALOX5AP	Absent	REACTOME_METABOLISM_OF_LIPIDS
ALOXE3	Absent	REACTOME_METABOLISM_OF_LIPIDS
ALPI	Absent	REACTOME_PHOSPHOLIPID_METABOLISM
ANGPTL4	Absent	REACTOME_METABOLISM_OF_LIPIDS
ANKRD1	Absent	REACTOME_METABOLISM_OF_LIPIDS
APOA1	Absent	REACTOME_METABOLISM_OF_LIPIDS
APOA2	Absent	REACTOME_METABOLISM_OF_LIPIDS
APOA5	Absent	REACTOME_METABOLISM_OF_LIPIDS
ARF1	Absent	REACTOME_PHOSPHOLIPID_METABOLISM
ARF3	Absent	REACTOME_PHOSPHOLIPID_METABOLISM
ARNT	Absent	REACTOME_METABOLISM_OF_LIPIDS
ARNT2	Absent	REACTOME_METABOLISM_OF_LIPIDS
ARNTL	Absent	REACTOME_METABOLISM_OF_LIPIDS
ARSD	Absent	REACTOME_METABOLISM_OF_LIPIDS
ARSF	Absent	REACTOME_METABOLISM_OF_LIPIDS
ARSG	Absent	REACTOME_METABOLISM_OF_LIPIDS
ARSH	Absent	REACTOME_METABOLISM_OF_LIPIDS
ARSI	Absent	REACTOME_METABOLISM_OF_LIPIDS
ARSJ	Absent	REACTOME_METABOLISM_OF_LIPIDS
ARSK	Absent	REACTOME_METABOLISM_OF_LIPIDS
ARSL	Absent	REACTOME_METABOLISM_OF_LIPIDS
ARV1	Absent	REACTOME_METABOLISM_OF_LIPIDS
AWAT1	Absent	REACTOME_METABOLISM_OF_LIPIDS
AWAT2	Absent	REACTOME_PHOSPHOLIPID_METABOLISM
BCHE	Absent	REACTOME_PHOSPHOLIPID_METABOLISM
BMX	Absent	REACTOME_PHOSPHOLIPID_METABOLISM
CARM1	Absent	REACTOME_METABOLISM_OF_LIPIDS
CAV1	Absent	REACTOME_METABOLISM_OF_LIPIDS
CCNC	Absent	REACTOME_METABOLISM_OF_LIPIDS
CDK19	Absent	REACTOME_METABOLISM_OF_LIPIDS
CDK8	Absent	REACTOME_METABOLISM_OF_LIPIDS
CERT1	Absent	REACTOME_METABOLISM_OF_LIPIDS

CHD9	Absent	REACTOME_METABOLISM_OF_LIPIDS
CIDEC	Absent	REACTOME_METABOLISM_OF_LIPIDS
CLOCK	Absent	REACTOME_METABOLISM_OF_LIPIDS
CPNE1	Absent	REACTOME_PHOSPHOLIPID_METABOLISM
CPNE3	Absent	REACTOME_PHOSPHOLIPID_METABOLISM
CPNE6	Absent	REACTOME_PHOSPHOLIPID_METABOLISM
CPNE7	Absent	REACTOME_PHOSPHOLIPID_METABOLISM
CPTP	Absent	REACTOME_METABOLISM_OF_LIPIDS
CREBBP	Absent	REACTOME_METABOLISM_OF_LIPIDS
CROT	Absent	REACTOME_METABOLISM_OF_LIPIDS
CSNK1G2	Absent	REACTOME_METABOLISM_OF_LIPIDS
CSNK2A1	Absent	REACTOME_PHOSPHOLIPID_METABOLISM
CSNK2A2	Absent	REACTOME_PHOSPHOLIPID_METABOLISM
CSNK2B	Absent	REACTOME_PHOSPHOLIPID_METABOLISM
CTSA	Absent	REACTOME_METABOLISM_OF_LIPIDS
CUBN	Absent	REACTOME_METABOLISM_OF_LIPIDS
CYP2D6	Absent	REACTOME_METABOLISM_OF_LIPIDS
CYP4B1	Absent	REACTOME_METABOLISM_OF_LIPIDS
CYP4F11	Absent	REACTOME_METABOLISM_OF_LIPIDS
CYP4F22	Absent	REACTOME_METABOLISM_OF_LIPIDS
DBI	Absent	REACTOME_METABOLISM_OF_LIPIDS
DDHD1	Absent	REACTOME_PHOSPHOLIPID_METABOLISM
DDHD2	Absent	REACTOME_PHOSPHOLIPID_METABOLISM
DECR2	Absent	REACTOME_METABOLISM_OF_LIPIDS
DGAT2L6	Absent	REACTOME_PHOSPHOLIPID_METABOLISM
DHRS7B	Absent	REACTOME_METABOLISM_OF_LIPIDS
DPEP1	Absent	REACTOME_METABOLISM_OF_LIPIDS
DPEP2	Absent	REACTOME_METABOLISM_OF_LIPIDS
DPEP3	Absent	REACTOME_METABOLISM_OF_LIPIDS
EP300	Absent	REACTOME_METABOLISM_OF_LIPIDS
ESRRA	Absent	REACTOME_METABOLISM_OF_LIPIDS
ESYT1	Absent	REACTOME_METABOLISM_OF_LIPIDS
ESYT2	Absent	REACTOME_METABOLISM_OF_LIPIDS
ESYT3	Absent	REACTOME_METABOLISM_OF_LIPIDS
FA2H	Absent	REACTOME_METABOLISM_OF_LIPIDS
FAAH	Absent	REACTOME_METABOLISM_OF_LIPIDS
FAAH2	Absent	REACTOME_METABOLISM_OF_LIPIDS
FABP12	Absent	REACTOME_METABOLISM_OF_LIPIDS
FABP3	Absent	REACTOME_METABOLISM_OF_LIPIDS
FABP4	Absent	REACTOME_METABOLISM_OF_LIPIDS
FABP5	Absent	REACTOME_METABOLISM_OF_LIPIDS
FABP6	Absent	REACTOME_METABOLISM_OF_LIPIDS
FABP7	Absent	REACTOME_METABOLISM_OF_LIPIDS
FABP9	Absent	REACTOME_METABOLISM_OF_LIPIDS
FAM120B	Absent	REACTOME_METABOLISM_OF_LIPIDS
FAR1	Absent	REACTOME_METABOLISM_OF_LIPIDS
FAR2	Absent	REACTOME_METABOLISM_OF_LIPIDS
FDPS	Absent	REACTOME_METABOLISM_OF_LIPIDS
FDX1	Absent	REACTOME_METABOLISM_OF_LIPIDS
FDX2	Absent	REACTOME_METABOLISM_OF_LIPIDS
FDXR	Absent	REACTOME_METABOLISM_OF_LIPIDS
FHL2	Absent	REACTOME_METABOLISM_OF_LIPIDS
FITM1	Absent	REACTOME_METABOLISM_OF_LIPIDS
FITM2	Absent	REACTOME_METABOLISM_OF_LIPIDS
GC	Absent	REACTOME_METABOLISM_OF_LIPIDS
GDE1	Absent	REACTOME_PHOSPHOLIPID_METABOLISM
GDPD5	Absent	REACTOME_PHOSPHOLIPID_METABOLISM
GGPS1	Absent	REACTOME_METABOLISM_OF_LIPIDS

GLIPR1	Absent	REACTOME_METABOLISM_OF_LIPIDS
GLTP	Absent	REACTOME_METABOLISM_OF_LIPIDS
GM2A	Absent	REACTOME_METABOLISM_OF_LIPIDS
GPS2	Absent	REACTOME_METABOLISM_OF_LIPIDS
GPX4	Absent	REACTOME_METABOLISM_OF_LIPIDS
GRHL1	Absent	REACTOME_METABOLISM_OF_LIPIDS
GSTM4	Absent	REACTOME_METABOLISM_OF_LIPIDS
HACL1	Absent	REACTOME_METABOLISM_OF_LIPIDS
HDAC3	Absent	REACTOME_METABOLISM_OF_LIPIDS
HELZ2	Absent	REACTOME_METABOLISM_OF_LIPIDS
HILPDA	Absent	REACTOME_METABOLISM_OF_LIPIDS
HMGCR	Absent	REACTOME_METABOLISM_OF_LIPIDS
HSD17B13	Absent	REACTOME_METABOLISM_OF_LIPIDS
HSD17B14	Absent	REACTOME_METABOLISM_OF_LIPIDS
IDI2	Absent	REACTOME_METABOLISM_OF_LIPIDS
INSIG1	Absent	REACTOME_METABOLISM_OF_LIPIDS
INSIG2	Absent	REACTOME_METABOLISM_OF_LIPIDS
KPNB1	Absent	REACTOME_METABOLISM_OF_LIPIDS
LGMN	Absent	REACTOME_METABOLISM_OF_LIPIDS
LHB	Absent	REACTOME_METABOLISM_OF_LIPIDS
LIPE	Absent	REACTOME_METABOLISM_OF_LIPIDS
LIPH	Absent	REACTOME_PHOSPHOLIPID_METABOLISM
LIPI	Absent	REACTOME_PHOSPHOLIPID_METABOLISM
LRP2	Absent	REACTOME_METABOLISM_OF_LIPIDS
MAPKAPK2	Absent	REACTOME_METABOLISM_OF_LIPIDS
MBTPS1	Absent	REACTOME_METABOLISM_OF_LIPIDS
MBTPS2	Absent	REACTOME_METABOLISM_OF_LIPIDS
MED1	Absent	REACTOME_METABOLISM_OF_LIPIDS
MED10	Absent	REACTOME_METABOLISM_OF_LIPIDS
MED11	Absent	REACTOME_METABOLISM_OF_LIPIDS
MED12	Absent	REACTOME_METABOLISM_OF_LIPIDS
MED13	Absent	REACTOME_METABOLISM_OF_LIPIDS
MED13L	Absent	REACTOME_METABOLISM_OF_LIPIDS
MED14	Absent	REACTOME_METABOLISM_OF_LIPIDS
MED15	Absent	REACTOME_METABOLISM_OF_LIPIDS
MED16	Absent	REACTOME_METABOLISM_OF_LIPIDS
MED17	Absent	REACTOME_METABOLISM_OF_LIPIDS
MED18	Absent	REACTOME_METABOLISM_OF_LIPIDS
MED19	Absent	REACTOME_METABOLISM_OF_LIPIDS
MED20	Absent	REACTOME_METABOLISM_OF_LIPIDS
MED21	Absent	REACTOME_METABOLISM_OF_LIPIDS
MED22	Absent	REACTOME_METABOLISM_OF_LIPIDS
MED23	Absent	REACTOME_METABOLISM_OF_LIPIDS
MED24	Absent	REACTOME_METABOLISM_OF_LIPIDS
MED25	Absent	REACTOME_METABOLISM_OF_LIPIDS
MED26	Absent	REACTOME_METABOLISM_OF_LIPIDS
MED27	Absent	REACTOME_METABOLISM_OF_LIPIDS
MED28	Absent	REACTOME_METABOLISM_OF_LIPIDS
MED29	Absent	REACTOME_METABOLISM_OF_LIPIDS
MED30	Absent	REACTOME_METABOLISM_OF_LIPIDS
MED31	Absent	REACTOME_METABOLISM_OF_LIPIDS
MED4	Absent	REACTOME_METABOLISM_OF_LIPIDS
MED6	Absent	REACTOME_METABOLISM_OF_LIPIDS
MED7	Absent	REACTOME_METABOLISM_OF_LIPIDS
MED8	Absent	REACTOME_METABOLISM_OF_LIPIDS
MED9	Absent	REACTOME_METABOLISM_OF_LIPIDS
MFSD2A	Absent	REACTOME_PHOSPHOLIPID_METABOLISM
MID1IP1	Absent	REACTOME_METABOLISM_OF_LIPIDS

MIGA1	Absent	REACTOME_PHOSPHOLIPID_METABOLISM
MIGA2	Absent	REACTOME_PHOSPHOLIPID_METABOLISM
MMAA	Absent	REACTOME_METABOLISM_OF_LIPIDS
MORC2	Absent	REACTOME_METABOLISM_OF_LIPIDS
MTF1	Absent	REACTOME_METABOLISM_OF_LIPIDS
MTMR10	Absent	REACTOME_PHOSPHOLIPID_METABOLISM
MTMR12	Absent	REACTOME_PHOSPHOLIPID_METABOLISM
MTMR9	Absent	REACTOME_PHOSPHOLIPID_METABOLISM
MVD	Absent	REACTOME_METABOLISM_OF_LIPIDS
MVK	Absent	REACTOME_METABOLISM_OF_LIPIDS
NCOA1	Absent	REACTOME_METABOLISM_OF_LIPIDS
NCOA2	Absent	REACTOME_METABOLISM_OF_LIPIDS
NCOA3	Absent	REACTOME_METABOLISM_OF_LIPIDS
NCOA6	Absent	REACTOME_METABOLISM_OF_LIPIDS
NCOR1	Absent	REACTOME_METABOLISM_OF_LIPIDS
NCOR2	Absent	REACTOME_METABOLISM_OF_LIPIDS
NFYA	Absent	REACTOME_METABOLISM_OF_LIPIDS
NFYB	Absent	REACTOME_METABOLISM_OF_LIPIDS
NFYC	Absent	REACTOME_METABOLISM_OF_LIPIDS
NPAS2	Absent	REACTOME_METABOLISM_OF_LIPIDS
NR1D1	Absent	REACTOME_METABOLISM_OF_LIPIDS
NR1H2	Absent	REACTOME_METABOLISM_OF_LIPIDS
NR1H3	Absent	REACTOME_METABOLISM_OF_LIPIDS
NR1H4	Absent	REACTOME_METABOLISM_OF_LIPIDS
NRF1	Absent	REACTOME_METABOLISM_OF_LIPIDS
NUDT19	Absent	REACTOME_METABOLISM_OF_LIPIDS
NUDT7	Absent	REACTOME_METABOLISM_OF_LIPIDS
ORMDL1	Absent	REACTOME_METABOLISM_OF_LIPIDS
ORMDL2	Absent	REACTOME_METABOLISM_OF_LIPIDS
ORMDL3	Absent	REACTOME_METABOLISM_OF_LIPIDS
OSBP	Absent	REACTOME_METABOLISM_OF_LIPIDS
OSBPL10	Absent	REACTOME_PHOSPHOLIPID_METABOLISM
OSBPL1A	Absent	REACTOME_METABOLISM_OF_LIPIDS
OSBPL2	Absent	REACTOME_METABOLISM_OF_LIPIDS
OSBPL3	Absent	REACTOME_METABOLISM_OF_LIPIDS
OSBPL5	Absent	REACTOME_PHOSPHOLIPID_METABOLISM
OSBPL6	Absent	REACTOME_METABOLISM_OF_LIPIDS
OSBPL7	Absent	REACTOME_METABOLISM_OF_LIPIDS
OSBPL8	Absent	REACTOME_PHOSPHOLIPID_METABOLISM
OSBPL9	Absent	REACTOME_METABOLISM_OF_LIPIDS
PCTP	Absent	REACTOME_PHOSPHOLIPID_METABOLISM
PECR	Absent	REACTOME_METABOLISM_OF_LIPIDS
PEX11A	Absent	REACTOME_METABOLISM_OF_LIPIDS
PHYH	Absent	REACTOME_METABOLISM_OF_LIPIDS
PIAS4	Absent	REACTOME_METABOLISM_OF_LIPIDS
PIK3R1	Absent	REACTOME_PHOSPHOLIPID_METABOLISM
PIK3R2	Absent	REACTOME_PHOSPHOLIPID_METABOLISM
PIK3R3	Absent	REACTOME_PHOSPHOLIPID_METABOLISM
PIK3R4	Absent	REACTOME_PHOSPHOLIPID_METABOLISM
PIK3R5	Absent	REACTOME_PHOSPHOLIPID_METABOLISM
PIK3R6	Absent	REACTOME_PHOSPHOLIPID_METABOLISM
PIP4P1	Absent	REACTOME_PHOSPHOLIPID_METABOLISM
PITPNB	Absent	REACTOME_PHOSPHOLIPID_METABOLISM
PITPNM1	Absent	REACTOME_PHOSPHOLIPID_METABOLISM
PITPNM2	Absent	REACTOME_PHOSPHOLIPID_METABOLISM
PITPNM3	Absent	REACTOME_PHOSPHOLIPID_METABOLISM
PLA2R1	Absent	REACTOME_PHOSPHOLIPID_METABOLISM
PLAAT1	Absent	REACTOME_PHOSPHOLIPID_METABOLISM

PLAAT2	Absent	REACTOME_PHOSPHOLIPID_METABOLISM
PLAAT4	Absent	REACTOME_PHOSPHOLIPID_METABOLISM
PLAAT5	Absent	REACTOME_PHOSPHOLIPID_METABOLISM
PLBD1	Absent	REACTOME_PHOSPHOLIPID_METABOLISM
PLD6	Absent	REACTOME_PHOSPHOLIPID_METABOLISM
PLEKHA1	Absent	REACTOME_PHOSPHOLIPID_METABOLISM
PLEKHA2	Absent	REACTOME_PHOSPHOLIPID_METABOLISM
PLEKHA3	Absent	REACTOME_PHOSPHOLIPID_METABOLISM
PLEKHA4	Absent	REACTOME_PHOSPHOLIPID_METABOLISM
PLEKHA5	Absent	REACTOME_PHOSPHOLIPID_METABOLISM
PLEKHA6	Absent	REACTOME_PHOSPHOLIPID_METABOLISM
PLEKHA8	Absent	REACTOME_PHOSPHOLIPID_METABOLISM
PLIN1	Absent	REACTOME_METABOLISM_OF_LIPIDS
PLIN2	Absent	REACTOME_METABOLISM_OF_LIPIDS
PLIN3	Absent	REACTOME_METABOLISM_OF_LIPIDS
PLPP6	Absent	REACTOME_METABOLISM_OF_LIPIDS
PMVK	Absent	REACTOME_METABOLISM_OF_LIPIDS
PNPLA4	Absent	REACTOME_METABOLISM_OF_LIPIDS
PNPLA5	Absent	REACTOME_METABOLISM_OF_LIPIDS
PNPLA8	Absent	REACTOME_PHOSPHOLIPID_METABOLISM
POMC	Absent	REACTOME_METABOLISM_OF_LIPIDS
PON1	Absent	REACTOME_METABOLISM_OF_LIPIDS
PON2	Absent	REACTOME_METABOLISM_OF_LIPIDS
PON3	Absent	REACTOME_METABOLISM_OF_LIPIDS
PPARD	Absent	REACTOME_METABOLISM_OF_LIPIDS
PPARG	Absent	REACTOME_METABOLISM_OF_LIPIDS
PPARGC1A	Absent	REACTOME_METABOLISM_OF_LIPIDS
PPARGC1B	Absent	REACTOME_METABOLISM_OF_LIPIDS
PPM1L	Absent	REACTOME_METABOLISM_OF_LIPIDS
PPP1CA	Absent	REACTOME_METABOLISM_OF_LIPIDS
PPP1CB	Absent	REACTOME_METABOLISM_OF_LIPIDS
PPP1CC	Absent	REACTOME_METABOLISM_OF_LIPIDS
PRKAA2	Absent	REACTOME_METABOLISM_OF_LIPIDS
PRKAB2	Absent	REACTOME_METABOLISM_OF_LIPIDS
PRKAG2	Absent	REACTOME_METABOLISM_OF_LIPIDS
PRKD1	Absent	REACTOME_METABOLISM_OF_LIPIDS
PRKD2	Absent	REACTOME_METABOLISM_OF_LIPIDS
PRKD3	Absent	REACTOME_METABOLISM_OF_LIPIDS
PTGR1	Absent	REACTOME_METABOLISM_OF_LIPIDS
PTGR2	Absent	REACTOME_METABOLISM_OF_LIPIDS
PTPMT1	Absent	REACTOME_PHOSPHOLIPID_METABOLISM
PTPN13	Absent	REACTOME_PHOSPHOLIPID_METABOLISM
RAB14	Absent	REACTOME_PHOSPHOLIPID_METABOLISM
RAB4A	Absent	REACTOME_PHOSPHOLIPID_METABOLISM
RAB5A	Absent	REACTOME_PHOSPHOLIPID_METABOLISM
RAN	Absent	REACTOME_METABOLISM_OF_LIPIDS
RGL1	Absent	REACTOME_METABOLISM_OF_LIPIDS
RORA	Absent	REACTOME_METABOLISM_OF_LIPIDS
RUFY1	Absent	REACTOME_PHOSPHOLIPID_METABOLISM
RXRA	Absent	REACTOME_METABOLISM_OF_LIPIDS
RXRB	Absent	REACTOME_METABOLISM_OF_LIPIDS
SAMD8	Absent	REACTOME_METABOLISM_OF_LIPIDS
SAR1B	Absent	REACTOME_METABOLISM_OF_LIPIDS
SBF1	Absent	REACTOME_PHOSPHOLIPID_METABOLISM
SBF2	Absent	REACTOME_PHOSPHOLIPID_METABOLISM
SCAP	Absent	REACTOME_METABOLISM_OF_LIPIDS
SEC23A	Absent	REACTOME_METABOLISM_OF_LIPIDS
SEC24A	Absent	REACTOME_METABOLISM_OF_LIPIDS

SEC24B	Absent	REACTOME_METABOLISM_OF_LIPIDS
SEC24C	Absent	REACTOME_METABOLISM_OF_LIPIDS
SEC24D	Absent	REACTOME_METABOLISM_OF_LIPIDS
SERPINA6	Absent	REACTOME_METABOLISM_OF_LIPIDS
SIN3A	Absent	REACTOME_METABOLISM_OF_LIPIDS
SIN3B	Absent	REACTOME_METABOLISM_OF_LIPIDS
SLC10A1	Absent	REACTOME_METABOLISM_OF_LIPIDS
SLC10A2	Absent	REACTOME_METABOLISM_OF_LIPIDS
SLC25A17	Absent	REACTOME_METABOLISM_OF_LIPIDS
SLC25A20	Absent	REACTOME_METABOLISM_OF_LIPIDS
SLC27A1	Absent	REACTOME_METABOLISM_OF_LIPIDS
SLC27A2	Absent	REACTOME_METABOLISM_OF_LIPIDS
SLC27A3	Absent	REACTOME_METABOLISM_OF_LIPIDS
SLC44A2	Absent	REACTOME_PHOSPHOLIPID_METABOLISM
SLC44A3	Absent	REACTOME_PHOSPHOLIPID_METABOLISM
SLC44A4	Absent	REACTOME_PHOSPHOLIPID_METABOLISM
SLC44A5	Absent	REACTOME_PHOSPHOLIPID_METABOLISM
SLC51A	Absent	REACTOME_METABOLISM_OF_LIPIDS
SLC51B	Absent	REACTOME_METABOLISM_OF_LIPIDS
SLCO1A2	Absent	REACTOME_METABOLISM_OF_LIPIDS
SLCO1B1	Absent	REACTOME_METABOLISM_OF_LIPIDS
SLCO1B3	Absent	REACTOME_METABOLISM_OF_LIPIDS
SMARCD3	Absent	REACTOME_METABOLISM_OF_LIPIDS
SP1	Absent	REACTOME_METABOLISM_OF_LIPIDS
SPNS2	Absent	REACTOME_METABOLISM_OF_LIPIDS
SPTSSA	Absent	REACTOME_METABOLISM_OF_LIPIDS
SPTSSB	Absent	REACTOME_METABOLISM_OF_LIPIDS
SREBF1	Absent	REACTOME_METABOLISM_OF_LIPIDS
SREBF2	Absent	REACTOME_METABOLISM_OF_LIPIDS
STAR	Absent	REACTOME_METABOLISM_OF_LIPIDS
STARD10	Absent	REACTOME_PHOSPHOLIPID_METABOLISM
STARD3	Absent	REACTOME_METABOLISM_OF_LIPIDS
STARD3NL	Absent	REACTOME_METABOLISM_OF_LIPIDS
STARD4	Absent	REACTOME_METABOLISM_OF_LIPIDS
STARD5	Absent	REACTOME_METABOLISM_OF_LIPIDS
STARD6	Absent	REACTOME_METABOLISM_OF_LIPIDS
STARD7	Absent	REACTOME_PHOSPHOLIPID_METABOLISM
SULT2A1	Absent	REACTOME_METABOLISM_OF_LIPIDS
SUMF1	Absent	REACTOME_METABOLISM_OF_LIPIDS
SUMF2	Absent	REACTOME_METABOLISM_OF_LIPIDS
SUMO2	Absent	REACTOME_METABOLISM_OF_LIPIDS
TBL1X	Absent	REACTOME_METABOLISM_OF_LIPIDS
TBL1XR1	Absent	REACTOME_METABOLISM_OF_LIPIDS
TECRL	Absent	REACTOME_METABOLISM_OF_LIPIDS
TGS1	Absent	REACTOME_METABOLISM_OF_LIPIDS
THRAP3	Absent	REACTOME_METABOLISM_OF_LIPIDS
THRSP	Absent	REACTOME_METABOLISM_OF_LIPIDS
TIAM2	Absent	REACTOME_METABOLISM_OF_LIPIDS
TNFAIP8	Absent	REACTOME_PHOSPHOLIPID_METABOLISM
TNFAIP8L1	Absent	REACTOME_PHOSPHOLIPID_METABOLISM
TNFAIP8L2	Absent	REACTOME_PHOSPHOLIPID_METABOLISM
TNFAIP8L3	Absent	REACTOME_PHOSPHOLIPID_METABOLISM
TNFRSF21	Absent	REACTOME_METABOLISM_OF_LIPIDS
TPTE	Absent	REACTOME_PHOSPHOLIPID_METABOLISM
TPTE2	Absent	REACTOME_PHOSPHOLIPID_METABOLISM
TRIB3	Absent	REACTOME_METABOLISM_OF_LIPIDS
TSPO	Absent	REACTOME_METABOLISM_OF_LIPIDS
TSPOAP1	Absent	REACTOME_METABOLISM_OF_LIPIDS

UBE2I	Absent	REACTOME_METABOLISM_OF_LIPIDS
VAC14	Absent	REACTOME_PHOSPHOLIPID_METABOLISM
VAPA	Absent	REACTOME_METABOLISM_OF_LIPIDS
VAPB	Absent	REACTOME_METABOLISM_OF_LIPIDS
VDR	Absent	REACTOME_METABOLISM_OF_LIPIDS
