# Supplementary material for: Comprehensive analyses of A 12-metabolism-associated gene signature and its connection with tumor metastases in clear cell renal cell carcinoma
Source: BMC Cancer. 2023 Mar 23;23:264. doi: 10.1186/s12885-023-10740-6 (PMC10035225; doi:10.1186/s12885-023-10740-6)
Supplement: Supplementary file 2 — Additional file 2: Supplementary Table S2. The genes and matching information in WGCNA. [file 12885_2023_10740_MOESM2_ESM.pdf]

Supplementary Table S2: The genes and matching information in WGCNA

Genesymbol	moduleColor	GS.variable	p.GS.variable
B4GALT4	black	0.432092276	0.003401026
RPL18A	black	0.414776181	0.00511926
RPL30	black	0.358146895	0.01698334
SARS1	black	0.340510607	0.023714211
RPS7	black	0.317786936	0.035543744
RPL13A	black	-0.288338917	0.057683549
RPL27	black	0.283825267	0.061889705
RPL19	black	0.275493346	0.070293828
RPS18	black	0.273996502	0.071895013
CHKB	black	-0.272934673	0.073048212
RPS2	black	0.270041249	0.076264722
RPL7A	black	-0.255509067	0.094134901
RPS17	black	-0.254137355	0.095976022
PI4KB	black	0.253036172	0.097474034
CERT1	black	-0.246874996	0.106190083
RPS12	black	-0.24554478	0.108147741
RPS8	black	0.235099086	0.124489875
KARS1	black	0.230087028	0.132960731
THRAP3	black	-0.225527195	0.141034285
RPL39	black	0.22461654	0.142689271
RPL18	black	0.222408917	0.146760872
CPT1B	black	-0.220835811	0.149714025
ACOX3	black	0.208614591	0.174158643
RPL23A	black	0.208492703	0.174416091
CSNK2B	black	0.205012075	0.18188372
MTMR4	black	0.202201119	0.1880793
RPL17	black	-0.193712805	0.207695231
PRKD3	black	0.184735381	0.229951233
APEX1	black	-0.18349576	0.233148095
RPLP0	black	0.179803653	0.242849107
RPS11	black	0.176126228	0.252779817
RPL21	black	-0.174792572	0.256447716
RPL5	black	0.173396108	0.260326349
RPS28	black	0.155018312	0.315010896
GNS	black	0.146815989	0.341605508
RPL23	black	0.143898987	0.351387481
CSPG5	black	-0.134266594	0.384886378
RPL36	black	0.131892683	0.393422012
CDK8	black	-0.126172167	0.414439196
ST3GAL1	black	-0.123623181	0.424006345
RPS14	black	0.123215827	0.425546721
PLA2G15	black	0.122140024	0.429629887
SMPD1	black	-0.119538564	0.439593736
OAZ1	black	0.117718767	0.446639029
RPL35A	black	0.115054427	0.457064728
PLA2G2C	black	-0.103029281	0.505718613
OSBPL8	black	0.102302246	0.508742028
INPP5F	black	-0.100964077	0.514330644
RPS25	black	0.093291283	0.546957571
INPPL1	black	-0.089557801	0.563182446
RPL7	black	-0.089473433	0.563551656
RPS5	black	0.088504552	0.567799731
NR1H2	black	-0.083639541	0.589350524

HSD17B11	black	0.081741293	0.597856719
MAPKAPK2	black	-0.081003359	0.601177903
RPS9	black	-0.079891323	0.606197885
RPL12	black	-0.078637748	0.611878364
RPL26	black	0.07801786	0.6146957
RPL27A	black	0.076991847	0.619370881
RPL4	black	-0.076646034	0.620949994
SUMO2	black	-0.07464217	0.630133364
RPL13	black	-0.069808668	0.652510242
VAC14	black	-0.068255129	0.659768124
ASL	black	0.068192844	0.66005976
NDC1	black	0.064522866	0.677330209
RPS13	black	0.062211355	0.688293098
ACBD6	black	0.061961644	0.689481246
FABP2	black	0.057475793	0.710948676
PLCD4	black	-0.05464859	0.724594172
RPL10A	black	0.052815362	0.73348783
PHOSPHO1	black	-0.042504065	0.784125151
GPS2	black	0.040130877	0.795913894
RPS4X	black	-0.036852846	0.812271014
MED30	black	-0.032416358	0.834533833
RPS10	black	0.030120205	0.846107756
CYP46A1	black	-0.029078961	0.851366941
SMYD3	black	0.026753771	0.863133857
RPS15A	black	0.022404006	0.88522323
RPL31	black	-0.016687047	0.914384251
CTSA	black	-0.012242996	0.937131408
RPS3	black	-0.011675066	0.940042338
RPLP1	black	-0.008381398	0.956938124
NUP35	black	-0.005042467	0.974085113
ACER2	black	-0.004194375	0.978442608
NUP54	black	-0.002377639	0.987778927
MTMR3	black	0.002277459	0.988293816
RBKS	blue	0.406834679	0.006132631
MED6	blue	-0.399511635	0.007216956
DERA	blue	0.398325476	0.007407346
DIO2	blue	0.377543582	0.011519853
AKR1C3	blue	0.367054761	0.014250145
ASRGL1	blue	-0.362561131	0.015578122
RAN	blue	0.357154656	0.017313546
ALDH4A1	blue	-0.345715006	0.021528934
TDO2	blue	0.338171547	0.024755111
SPNS2	blue	-0.333037384	0.027174039
SLC26A1	blue	0.326544111	0.030511703
SLC6A12	blue	-0.317322473	0.035828623
LPIN1	blue	-0.314863935	0.037367996
SLC27A2	blue	-0.313945689	0.037956675
CBR3	blue	0.308152139	0.041848497
CHDH	blue	-0.304229885	0.044663532
CERS2	blue	0.303626842	0.045109683
GLB1L	blue	0.294033221	0.052707247
ACSM5	blue	0.292184579	0.054283377
BCAT1	blue	0.287133962	0.058783334
RPSA	blue	-0.282765655	0.062911738
CHST13	blue	0.281328245	0.064319613
ALDH3A2	blue	-0.281129153	0.064516573
FASN	blue	0.272655431	0.073353892
PSMD10	blue	0.270825683	0.075381899

PRODH2	blue	-0.265828055	0.081146214
AGMAT	blue	-0.264973633	0.082165331
CYP4A11	blue	-0.260465612	0.087708864
SAR1B	blue	0.26023819	0.087996042
ENOSF1	blue	-0.258749605	0.089893844
DECR2	blue	0.258537613	0.090166676
AGT	blue	0.257734721	0.091205817
CYP2C8	blue	0.255919295	0.093589625
SLC22A5	blue	0.255426906	0.094244403
RPL32	blue	-0.254884259	0.094970109
RGL1	blue	0.251813676	0.099158102
GATM	blue	-0.249623205	0.102231474
ACAA2	blue	-0.247534675	0.105229346
CYP3A5	blue	0.246033044	0.107425998
NIT2	blue	0.241961679	0.113557872
RPL14	blue	-0.241569802	0.114161809
CYP27A1	blue	-0.237160267	0.121126513
GLUD1	blue	0.232881396	0.128186503
NUP50	blue	0.232476822	0.128869657
GPC4	blue	-0.229748831	0.133547426
CYB5R3	blue	0.225698644	0.140724298
DHTKD1	blue	-0.225387923	0.141286468
MED29	blue	-0.22407618	0.143678065
PRKD1	blue	-0.221063971	0.149283019
GALK2	blue	0.21602585	0.159014462
ST6GALNAC6	blue	-0.215940913	0.159182395
MRI1	blue	-0.214766206	0.161518235
ST3GAL3	blue	0.2140184	0.163018141
SLC44A3	blue	-0.213941772	0.163172407
RPL34	blue	-0.212785078	0.165513936
SLC10A2	blue	-0.211286563	0.168583531
ACADL	blue	-0.205450839	0.180929971
HSD11B1	blue	0.201557878	0.189517906
HAS3	blue	0.198315506	0.196888475
ADPRM	blue	-0.196743741	0.200533277
DDC	blue	-0.194908277	0.204849335
BHMT2	blue	0.194713305	0.205311604
UROD	blue	0.192224204	0.211277416
HAS2	blue	0.191564898	0.212877656
UGT1A10	blue	0.190047251	0.216593216
H2AZ1	blue	0.189734821	0.217363667
CRYL1	blue	-0.18722071	0.223632633
GPT	blue	-0.183945095	0.231985812
APOA1	blue	0.183538379	0.233037682
PLD1	blue	0.182464985	0.23582939
HGD	blue	0.182144303	0.236667831
D2HGDH	blue	-0.182092415	0.236803687
NUP37	blue	0.179401304	0.243922561
BBOX1	blue	-0.175616527	0.254177444
LIPC	blue	-0.175361837	0.254877756
PLA2G12B	blue	0.175224221	0.255256692
SLC37A4	blue	0.172766519	0.262087737
CARM1	blue	-0.171015307	0.267028726
AMDHD1	blue	-0.169978428	0.269983155
SEPSECS	blue	-0.168904259	0.273066539
NUP93	blue	0.166123088	0.281157307
PYGB	blue	0.165793416	0.282126653
PAOX	blue	0.165338054	0.283469152

PTGIS	blue	0.16392633	0.28765767
PSMB5	blue	-0.162774167	0.29110575
SHMT1	blue	-0.162533062	0.291830681
RXRB	blue	-0.159963965	0.299627722
PLEKHA1	blue	0.159955808	0.29965269
BHMT	blue	-0.158191415	0.305084631
GBA3	blue	-0.157033114	0.308684649
PEDS1-UBE2V1	blue	-0.152523134	0.322958428
ALDH1A1	blue	-0.152481861	0.323090938
DGKB	blue	-0.152396239	0.323365944
ARSL	blue	0.152002804	0.324631497
RPL29	blue	0.151549232	0.326094339
CPNE1	blue	0.151090351	0.3275785
MAOB	blue	0.150465474	0.329606323
B3GALNT1	blue	0.150141242	0.330661589
SLC25A13	blue	0.149693803	0.332121308
PLEKHA6	blue	-0.148777788	0.335122192
CRYM	blue	-0.147744938	0.338525953
AGMO	blue	0.147445235	0.339517615
CRPPA	blue	-0.145776091	0.345073294
MLYCD	blue	0.145631584	0.345556893
PNPLA3	blue	0.145605996	0.345642566
GPX3	blue	-0.144335602	0.349912539
L3HYPDH	blue	0.14348498	0.352789548
SDC1	blue	0.142191634	0.357191499
PSMD13	blue	0.141220409	0.360518914
CEMIP	blue	0.140361531	0.363476991
LUM	blue	0.140202028	0.364027944
PTGES3	blue	0.139490109	0.366493178
CYP1B1	blue	0.139158581	0.367644609
UGT2B10	blue	-0.138994791	0.368214269
ACAD11	blue	0.136551042	0.376776369
SERPINA6	blue	-0.136198832	0.378020078
EXT2	blue	0.134412515	0.384365288
ACBD4	blue	-0.131971181	0.393138011
NCOR1	blue	-0.131962756	0.393168487
PLPP3	blue	-0.131924975	0.393305166
ENO4	blue	0.131854196	0.393561297
GLS	blue	-0.129563431	0.401903491
PLA2G4C	blue	0.128534068	0.405685119
MBOAT7	blue	-0.127716605	0.408702819
FMO1	blue	-0.126472668	0.413319489
CSNK2A1	blue	-0.124896812	0.419210536
GPD1	blue	-0.123671962	0.423822093
PLA2G3	blue	0.123534758	0.424340443
PRODH	blue	0.122338389	0.428875357
PEX11A	blue	-0.121900329	0.430542619
UGT1A7	blue	0.121031526	0.433859994
RPS26	blue	-0.119830421	0.43846957
DDO	blue	0.118659085	0.442990911
ASPG	blue	-0.117876989	0.44602403
RPS21	blue	0.117323938	0.448175731
MARS1	blue	0.115568113	0.45504446
GNPDA1	blue	-0.114491281	0.459285087
MGAM	blue	-0.113951179	0.461420045
SLC51A	blue	-0.112853464	0.465775598
CRYZ	blue	0.110305435	0.475970047
LHB	blue	0.108270461	0.484195687

FADS2	blue	0.108125923	0.48478274
NUP210	blue	0.10746863	0.487457072
ACSM2A	blue	-0.104495393	0.499649553
UGT2B17	blue	-0.103495125	0.503786178
UST	blue	-0.103461489	0.503925585
MPI	blue	0.102582518	0.507575421
CPT1C	blue	0.102514337	0.507859091
SERINC4	blue	0.101938515	0.510258038
PECR	blue	-0.101831207	0.510705728
KL	blue	-0.100878832	0.514687691
B4GALT3	blue	-0.098711502	0.523807082
NUP188	blue	0.098618265	0.524201174
PLBD1	blue	-0.098467369	0.52483929
ACSM2B	blue	-0.096896101	0.531506631
HAAO	blue	-0.09659251	0.532799609
FCSK	blue	0.096396288	0.533636121
MFSD2A	blue	-0.093112408	0.547729806
ENPP2	blue	0.090832501	0.557617804
ACACA	blue	0.08851315	0.567761968
CNDP2	blue	0.086399195	0.577081268
UGT2B11	blue	-0.08607113	0.578533729
ARNT	blue	-0.085536305	0.580905143
PIP5K1C	blue	0.083651749	0.589295993
CYB5R2	blue	-0.083427562	0.590297745
ALPI	blue	0.080549708	0.6032236
LPCAT4	blue	0.079467737	0.608114789
CUBN	blue	-0.079088213	0.6098345
CERS5	blue	0.078422906	0.612854178
AGXT2	blue	-0.078345567	0.613205613
LIPE	blue	-0.077408448	0.617470772
MAOA	blue	-0.076412845	0.62201577
KHK	blue	-0.075645733	0.625527204
ACMSD	blue	0.074908916	0.628907689
SYNJ1	blue	0.07463645	0.630159658
TBL1X	blue	-0.07184122	0.64306225
CHAT	blue	-0.071519651	0.644553361
DCN	blue	0.071314113	0.645507163
ELOVL3	blue	0.070682338	0.64844243
PLAAT3	blue	0.068620683	0.658057507
PAFAH1B3	blue	-0.067274617	0.66436491
CHST9	blue	-0.065487144	0.672776133
RPL11	blue	-0.064754912	0.676233257
CIDEA	blue	0.063929099	0.680140116
ASMT	blue	-0.06357415	0.68182191
HSD17B14	blue	-0.062740766	0.685776562
GGT1	blue	0.061871197	0.689911788
GPC2	blue	0.061389178	0.692207877
SERINC2	blue	-0.061125027	0.69346732
EHHADH	blue	-0.061043354	0.693856894
HYAL3	blue	-0.060300378	0.697404389
FAH	blue	0.059767907	0.699950716
AKR1A1	blue	-0.059707539	0.700239609
ASPA	blue	-0.058898058	0.704117407
MTMR12	blue	-0.058629742	0.705404412
ECH1	blue	-0.055761825	0.7192108
PKLR	blue	-0.055096447	0.722426834
B4GAT1	blue	0.055035705	0.722720658
TKT	blue	0.053952075	0.727969076

OSBPL9	blue	-0.053731198	0.729040384
TIAM2	blue	-0.0504744	0.744894869
UGT1A6	blue	0.048971759	0.752245626
GAMT	blue	-0.048396278	0.755066603
AMY2B	blue	-0.047916124	0.757422705
SERINC1	blue	0.047666731	0.758647326
PLPP1	blue	0.047370055	0.76010489
PSMD1	blue	0.047195378	0.760963458
HS3ST3A1	blue	0.04609351	0.766385843
AMY2A	blue	0.045202621	0.770778069
RIMKLB	blue	-0.044453026	0.774479196
SETD7	blue	0.044359226	0.774942682
AOC1	blue	-0.043711546	0.778145124
SLC35D2	blue	-0.043084947	0.781246787
MAT2A	blue	-0.042548476	0.783904985
PSMF1	blue	0.041628595	0.788468623
SGMS2	blue	0.040416855	0.794490873
AMACR	blue	-0.039992787	0.796601267
CSGALNACT1	blue	0.039273998	0.800181622
PFKL	blue	0.038337641	0.804851774
TNFRSF21	blue	-0.036657922	0.813246234
ACOT9	blue	-0.035198904	0.820554591
SCLY	blue	0.034908462	0.822011255
PLD6	blue	0.034200559	0.825564098
PRELP	blue	0.031796352	0.83765572
DMGDH	blue	0.030894793	0.842199682
SEC13	blue	0.030309915	0.845150258
AZIN1	blue	-0.027833452	0.857666195
UGT2B7	blue	0.027191749	0.860915095
DSEL	blue	0.026997661	0.861898198
ALDH2	blue	0.026296306	0.865452446
SGPL1	blue	-0.026281895	0.865525505
SMS	blue	0.02484182	0.872831617
RIDA	blue	-0.024561676	0.874254143
GFUS	blue	-0.021684605	0.888885315
HNMT	blue	-0.021561945	0.889509941
RAE1	blue	-0.021456959	0.890044619
RORA	blue	0.020974519	0.892502227
ADSS1	blue	-0.020831974	0.893228561
GPD2	blue	-0.020325226	0.895811369
ACADVL	blue	-0.019173724	0.901684306
RPS27A	blue	0.018603362	0.904595229
SQLE	blue	-0.018546514	0.904885429
PSMD7	blue	-0.017421177	0.910632568
UGT2B28	blue	-0.016896539	0.913313474
INPP1	blue	-0.016524613	0.915214598
LRP2	blue	0.015657571	0.91964834
UGT1A9	blue	-0.015502184	0.920443191
GSTM4	blue	-0.011795203	0.939426507
AMD1	blue	-0.011236609	0.942290185
ST3GAL2	blue	0.01117988	0.942581053
CHST2	blue	0.006922554	0.964428063
PNPLA7	blue	-0.004924994	0.974688649
B3GALT6	blue	-0.004755937	0.975557229
PMM1	blue	0.004593232	0.976393195
UGT2A3	blue	0.00308786	0.984128797
NANS	blue	-0.0021186	0.989110302
SAMD8	blue	0.001267862	0.993483006

ARF3	blue	-0.000135443	0.999303798
RDH11	brown	-0.711152431	6.31E-08
P4HA1	brown	0.699314339	1.29E-07
CKMT1A	brown	-0.647252397	2.05E-06
GPD1L	brown	-0.645404526	2.24E-06
PIP5K1B	brown	-0.635070697	3.65E-06
HSD17B12	brown	-0.633220126	3.97E-06
HSD11B2	brown	-0.627844895	5.07E-06
PLA2G4F	brown	-0.617928616	7.86E-06
CKMT1B	brown	-0.610252608	1.09E-05
TM7SF2	brown	-0.597690107	1.84E-05
PRDM16	brown	-0.573287979	4.75E-05
INPP5J	brown	-0.571626337	5.06E-05
PIP4P1	brown	-0.563712596	6.76E-05
MIGA2	brown	-0.549005454	0.000113848
PFKM	brown	-0.543071962	0.00013953
HACD3	brown	-0.542556937	0.00014199
PIP4K2C	brown	-0.537454509	0.000168575
BDH1	brown	-0.531914823	0.000202478
PRPS1	brown	0.510434424	0.000400247
GPAT3	brown	-0.500022832	0.000548125
PLCG2	brown	-0.499320861	0.000559668
SLC27A3	brown	0.494681868	0.000641594
SC5D	brown	-0.47164323	0.00122985
ACACB	brown	-0.465916284	0.001435829
LDHD	brown	-0.465282239	0.001460419
CHP1	brown	-0.462985506	0.00155265
DNMT3A	brown	-0.453325075	0.001999669
OSBPL2	brown	-0.448086645	0.002286802
STARD10	brown	-0.44448365	0.002504846
CRAT	brown	-0.441106142	0.002725675
TMLHE	brown	-0.44072397	0.00275171
PRPS2	brown	-0.43558524	0.003123651
HS2ST1	brown	0.434167906	0.003233709
MED9	brown	-0.430939931	0.003497156
CHKA	brown	-0.429613825	0.003610723
PFKFB2	brown	-0.427161755	0.00382925
CYP51A1	brown	-0.424476446	0.004081763
SLC3A2	brown	-0.423642765	0.004163065
HSP90AA1	brown	-0.423400569	0.004186948
ITPK1	brown	-0.422752775	0.004251416
CKB	brown	-0.4210265	0.004427469
SACM1L	brown	-0.420236767	0.004510108
SETMAR	brown	-0.41733776	0.004825122
SUMF1	brown	-0.415331817	0.005054165
MED23	brown	0.412010815	0.005454208
PTPMT1	brown	-0.411715711	0.005491053
AHCYL2	brown	-0.408634459	0.005888872
ACSS3	brown	-0.398978814	0.007301949
PTGES2	brown	-0.397246391	0.007584305
GABARAPL1	brown	-0.394957992	0.007971699
GOT2	brown	-0.394857656	0.007989068
OSTC	brown	0.394684574	0.008019106
ABCC5	brown	-0.389996685	0.008870602
CDK19	brown	0.384773508	0.009909897
HACD1	brown	-0.381864311	0.010532845
CROT	brown	-0.380042961	0.010939695
NUP160	brown	0.378831994	0.011217583

FUT1	brown	-0.378765036	0.011233123
ABHD4	brown	-0.378169696	0.011372104
PSMB1	brown	0.375615168	0.011985273
PISD	brown	-0.375402385	0.012037598
PRKACB	brown	-0.373950419	0.012399884
ST3GAL6	brown	-0.371981315	0.012906039
HDAC3	brown	0.36649152	0.014411132
CYP3A7	brown	-0.363769712	0.015211038
IDI1	brown	-0.357716794	0.017125813
BPGM	brown	-0.35741291	0.017227084
CYP4F8	brown	-0.354922232	0.018076352
SGMS1	brown	-0.349461912	0.020062442
FA2H	brown	-0.349337607	0.020109709
MTMR6	brown	0.348930087	0.020265329
AGPAT2	brown	-0.343916535	0.022264157
BMPR1B	brown	-0.341418083	0.023320353
TNFAIP8	brown	0.340636286	0.023659335
VDR	brown	-0.340607471	0.023671908
ACOT8	brown	-0.339898854	0.023982847
CKMT2	brown	-0.339607134	0.024111841
GDE1	brown	-0.338849321	0.024449646
MECR	brown	-0.338680103	0.024525614
ESRRA	brown	-0.338327923	0.024684353
MTMR9	brown	-0.337498819	0.025061445
SPTSSA	brown	-0.333917297	0.02674605
DHCR7	brown	-0.328191617	0.029634313
NAT8L	brown	-0.327985821	0.029742755
CS	brown	-0.327823084	0.02982874
DGAT1	brown	-0.323401073	0.032245201
GLTP	brown	-0.3222866	0.032879073
ABHD3	brown	-0.309248827	0.041087827
PRKAG2	brown	-0.308197202	0.041817016
IDH3A	brown	-0.305018901	0.044085215
ST3GAL4	brown	-0.304785088	0.044255952
RPS20	brown	0.304603984	0.044388569
CPTP	brown	-0.303194264	0.045431944
BCAT2	brown	-0.301444002	0.046754989
FDX2	brown	-0.299045596	0.048618536
ACSF3	brown	-0.298222027	0.049272136
SLC25A12	brown	-0.297937118	0.049499892
FPGT	brown	0.29582926	0.051211473
SLC25A17	brown	-0.288052334	0.057943612
ARSB	brown	0.282471644	0.063197692
HCCS	brown	-0.278567483	0.067093869
HSD17B1	brown	-0.277016028	0.068694067
NUP43	brown	0.274506482	0.071346282
EBP	brown	-0.273461803	0.072473913
MED25	brown	-0.273149162	0.072814099
AGPS	brown	0.270669319	0.07555723
IDI2	brown	0.268378085	0.078163362
MSMO1	brown	-0.259185473	0.089334897
PCBD1	brown	-0.245828711	0.10772759
SLC27A1	brown	-0.245558151	0.108127927
GAD1	brown	-0.245305598	0.108502642
HS6ST3	brown	-0.241791386	0.113820018
ORMDL2	brown	-0.240667178	0.115562144
ARSG	brown	0.239618274	0.117205723
SDC4	brown	-0.235600265	0.123665683

UGT1A1	brown	0.234636856	0.125253668
PLA2G4A	brown	-0.226988935	0.138407576
PLEKHA2	brown	0.226780402	0.138780067
FAM120B	brown	0.224192978	0.143463912
TNFAIP8L3	brown	-0.218022097	0.155104713
HMGCR	brown	-0.21578741	0.15948622
DDHD1	brown	-0.214135913	0.162781772
PGP	brown	-0.210310161	0.170605642
ELOVL6	brown	-0.209331716	0.172649494
LSS	brown	-0.208329973	0.174760231
CEL	brown	-0.207238565	0.177080959
PHKG2	brown	-0.201768287	0.189046469
AGRN	brown	0.197000153	0.19993546
NUP98	brown	-0.19311836	0.209120583
ALAS1	brown	-0.192750601	0.210005803
PIK3R1	brown	0.188492107	0.220446991
PTDSS2	brown	-0.1882284	0.22110515
ETNK1	brown	-0.187538212	0.222834136
CSGALNACT2	brown	0.187273101	0.223500738
GGPS1	brown	-0.18460886	0.230276135
PTS	brown	-0.175564267	0.254321035
GNPNAT1	brown	-0.173478431	0.260096619
VAPA	brown	-0.171399871	0.265938437
INSIG1	brown	-0.170746974	0.267791236
PNPLA8	brown	-0.157151103	0.308316703
GPAM	brown	-0.155252354	0.314271845
GPAT2	brown	0.152484775	0.323081582
SLCO1B3	brown	-0.150952891	0.328023909
ARSK	brown	-0.148431239	0.336261869
TPH2	brown	-0.148168904	0.337126192
TNFAIP8L1	brown	0.146394104	0.343009782
CPT1A	brown	-0.143413529	0.353031868
THEM4	brown	0.139249951	0.367327055
DGKI	brown	-0.137864835	0.372158655
ELOVL4	brown	-0.137499035	0.37344096
MED19	brown	-0.137231246	0.374381354
ADIPOR2	brown	0.135860885	0.379215713
PTGS2	brown	-0.134863097	0.382758878
AACS	brown	-0.132771756	0.390248416
NUP58	brown	0.132181514	0.392377627
NSD3	brown	-0.128404426	0.40616284
CDS2	brown	-0.125367433	0.417446239
ADSL	brown	0.122620397	0.427803946
HMGCS1	brown	-0.121549493	0.431880514
ALOXE3	brown	0.117725489	0.446612894
B4GALT6	brown	-0.112466334	0.467316899
RAB5A	brown	-0.110025224	0.477098298
SLC9A1	brown	-0.109148708	0.480636611
MID1IP1	brown	-0.102212431	0.509116158
UAP1L1	brown	-0.100621266	0.515767264
PGK2	brown	-0.095316234	0.538251914
PRDM9	brown	-0.083600464	0.589525087
INPP4B	brown	-0.078525499	0.612388117
CPNE3	brown	0.075957582	0.62409873
PIK3R2	brown	0.074212845	0.632108135
PRKAB2	brown	-0.072535875	0.639845876
ARSJ	brown	-0.069205556	0.655324168
COMT	brown	0.06793684	0.661258963

LPCAT2	brown	-0.066243488	0.669212152
PLCB3	brown	0.050872019	0.742953465
CERK	brown	0.048048748	0.756771703
HSPH1	brown	-0.047627641	0.75883933
RAP1GDS1	brown	-0.043599126	0.778701351
SELENOI	brown	-0.042357412	0.784852294
CYP11B2	brown	0.04217491	0.785757436
PHKA1	brown	-0.038442567	0.804328112
LBR	brown	0.037307122	0.809999326
LTA4H	brown	0.033924859	0.826948728
MDH2	brown	-0.03264536	0.833381382
CH25H	brown	-0.019862843	0.898168992
HACD2	brown	0.019399507	0.900532337
UAP1	brown	0.018696691	0.904118825
ACSBG1	brown	-0.010168544	0.947767679
GMPPB	brown	-0.009949803	0.948889778
PLCE1	brown	0.007214844	0.96292714
DLST	green	-0.700622619	1.19E-07
OGDHL	green	-0.658283594	1.19E-06
GSTZ1	green	-0.635251526	3.62E-06
PPARGC1A	green	-0.634844516	3.69E-06
CDS1	green	-0.612993868	9.72E-06
ELOVL5	green	0.596140346	1.96E-05
AUH	green	-0.558935457	8.03E-05
GBA2	green	-0.545060716	0.00013039
SECISBP2	green	-0.535343999	0.000180833
MED8	green	0.530977205	0.000208791
UGT8	green	-0.529711952	0.000217593
H6PD	green	0.527121728	0.000236666
MTMR10	green	-0.511953144	0.000381982
UGDH	green	0.499200434	0.00056167
SDHD	green	-0.492379662	0.000686111
PON2	green	0.491849178	0.000696752
CAT	green	-0.486773778	0.000806287
NUP107	green	0.475642748	0.001102032
HELZ2	green	0.469353698	0.00130881
FABP3	green	-0.465275273	0.001460691
HYAL1	green	-0.461088201	0.0016327
ACOT1	green	-0.459101071	0.001720443
HMMR	green	0.451745681	0.002082681
ETFDH	green	-0.450038756	0.002175808
NUP153	green	0.448784112	0.002246584
HSDL2	green	-0.443113306	0.002592465
MED1	green	0.439974434	0.002803408
TXN2	green	-0.439706336	0.002822106
OXCT1	green	-0.424953684	0.00403585
EZH2	green	0.423205045	0.004206316
FUT4	green	0.423025159	0.004224204
OXSM	green	-0.422703052	0.0042564
PSMB3	green	0.419235588	0.004616807
PLIN3	green	0.415181204	0.005071739
PSMA4	green	0.410063864	0.005701314
PAFAH2	green	-0.40989023	0.005723817
ACOT4	green	-0.399853215	0.007162919
PHYH	green	-0.399468703	0.007223772
CAD	green	0.396409126	0.007724113
DECR1	green	-0.395809075	0.007825674
PNPLA4	green	-0.38597362	0.009662285

MED11	green	-0.385051442	0.009852073
OGDH	green	-0.377268081	0.011585383
CHSY1	green	0.376575559	0.011751515
PSMD14	green	0.364912738	0.014870644
CHST1	green	0.360570975	0.016198957
SDHC	green	-0.359879566	0.016419491
PIK3C2A	green	-0.359588688	0.016513027
PSMA5	green	0.359078497	0.016678175
NUDT7	green	-0.356430839	0.017557833
PRKAA2	green	-0.341915696	0.023106714
PPAT	green	0.340746213	0.023611424
SERINC3	green	0.338217244	0.024734416
GPCPD1	green	0.333609417	0.02689516
HSD17B4	green	-0.330859798	0.028257758
ECI1	green	-0.328860447	0.02928414
PSMB2	green	0.326548929	0.030509106
CBR1	green	-0.32490663	0.03140495
GOT1	green	-0.323931925	0.031946839
PYCR1	green	0.318282082	0.035242098
ACO2	green	-0.3182532	0.035259635
HIBADH	green	-0.316200352	0.036524628
PDHX	green	-0.314118252	0.037845471
OSBPL1A	green	-0.313718134	0.038103728
SEC24C	green	0.313322457	0.038360537
SLC35B2	green	0.312851519	0.03866804
CHPT1	green	-0.311466879	0.039583862
DNMT1	green	0.300660399	0.047357363
ME3	green	-0.298886452	0.048744286
FH	green	-0.29812621	0.049348638
PPARG	green	-0.297953109	0.049487087
PGS1	green	0.288427115	0.057603701
PNPLA2	green	-0.283508825	0.062193528
DLD	green	-0.282753132	0.062923897
HADHB	green	-0.27743762	0.068256268
NUP85	green	0.276530648	0.069200855
CLOCK	green	-0.270868533	0.075333907
OSBP	green	-0.269913454	0.076409313
BCKDHA	green	-0.266313789	0.080571276
RPIA	green	0.26335058	0.084128758
BCKDHB	green	-0.257555673	0.09143881
ADO	green	0.257302161	0.091769491
PPP2R5D	green	0.255393208	0.094289344
PTDSS1	green	0.25276268	0.097848862
SLC25A2	green	0.25197562	0.098933737
AGL	green	-0.244144626	0.110237936
SBF2	green	-0.24313597	0.111762646
IDH2	green	-0.242604778	0.112572027
ORMDL1	green	0.240956369	0.115112081
ACADS	green	-0.239589179	0.117251564
CYB5R1	green	-0.238293128	0.11930737
ACBD5	green	-0.238026896	0.119733018
MTR	green	-0.234940111	0.12475217
DLAT	green	-0.233973807	0.126355437
CA2	green	-0.233612762	0.126958424
LIAS	green	-0.22375126	0.144275063
PMVK	green	-0.223560973	0.144625541
ARV1	green	-0.220833678	0.149718059
PRDX6	green	-0.2171946	0.156716782

HAS1	green	0.21210039	0.1669114
PLPP2	green	0.199488342	0.194199388
CERS4	green	-0.196067882	0.202115043
NFYA	green	0.195285263	0.203957594
IMPA2	green	-0.192132559	0.211499351
SYNJ2	green	0.189567916	0.217776034
UGT2A2	green	0.16779961	0.276261531
HADHA	green	-0.165719047	0.282345622
OSBPL7	green	0.159410515	0.301324773
SDHA	green	-0.156015951	0.311868232
ELOVL7	green	-0.147722781	0.338599204
NCOA3	green	0.146527004	0.342567033
GPX7	green	0.146411694	0.342951163
PHKB	green	-0.143866042	0.351498926
STARD3	green	0.143602262	0.352392018
SLC26A2	green	0.141020522	0.361206044
IDH3B	green	-0.138332791	0.37052209
NRF1	green	0.13530724	0.381179308
KDSR	green	-0.131829838	0.393649467
CHST7	green	0.131260731	0.395712736
LPCAT3	green	0.130234129	0.399450515
KPNB1	green	0.12987443	0.400764978
GSR	green	-0.122329639	0.428908624
SMOX	green	0.121537997	0.431924394
NDUFAB1	green	-0.117365398	0.448014231
PSMA2	green	-0.109578588	0.478899555
SLC25A1	green	-0.105427943	0.495808694
AGPAT5	green	-0.100161124	0.517698732
ACSS1	green	-0.100124019	0.517854637
ACSL1	green	-0.094078916	0.543563415
MMAA	green	-0.092488647	0.550426744
NUDT19	green	-0.087862264	0.57062397
SDC2	green	-0.084419995	0.585868931
PLD2	green	0.083993028	0.587772487
PLCG1	green	0.062479841	0.687016441
PSAPL1	green	-0.050313565	0.745680596
DDHD2	green	-0.048496662	0.754574295
TP53INP2	green	-0.04611136	0.766297914
UROS	green	0.045023281	0.771663107
SEH1L	green	-0.038626647	0.803409609
GPX4	green	-0.024545818	0.874334675
TPTE2	green	0.021669412	0.88896268
TXNRD1	greenyellow	0.41023973	0.0056786
AKR1B10	greenyellow	0.29148435	0.054890189
CYP21A2	greenyellow	0.289919504	0.056265975
AKR1B15	greenyellow	0.274459896	0.07139627
PIK3CB	greenyellow	0.244132591	0.110256034
FDX1	greenyellow	-0.231272631	0.130919161
CYP4F11	greenyellow	0.229351917	0.134238442
SULT2A1	greenyellow	0.204640803	0.182693562
GCLM	greenyellow	0.184694857	0.230055262
CYP11B1	greenyellow	0.179314724	0.244153972
HSD3B2	greenyellow	0.173150092	0.26101368
KMT2C	greenyellow	0.168811744	0.273333183
ACAT2	greenyellow	-0.165081345	0.28422782
CARNMT1	greenyellow	-0.157508575	0.307203647
GCLC	greenyellow	-0.139820574	0.365347596
SMPD2	greenyellow	0.128820735	0.404629921

NQO1	greenyellow	0.126047885	0.41490279
CYP11A1	greenyellow	0.110128365	0.476682846
EEFSEC	greenyellow	0.106043968	0.49327985
DHCR24	greenyellow	0.105558523	0.495272092
STAR	greenyellow	0.100275861	0.517216782
NSDHL	greenyellow	0.097350924	0.52957244
CYP17A1	greenyellow	-0.073274324	0.63643387
EHMT2	greenyellow	0.069896076	0.652102819
AKR1C2	greenyellow	0.064511906	0.677382033
AKR1C4	greenyellow	0.063661875	0.681406117
PCTP	greenyellow	0.059202791	0.702656714
TALDO1	greenyellow	-0.042085477	0.786201092
OAZ2	greenyellow	-0.035429826	0.819396862
ESYT3	greenyellow	0.028549279	0.85404475
PON1	greenyellow	0.01825876	0.906354555
LDHC	greenyellow	0.017738464	0.9090117
MVD	greenyellow	0.012302234	0.936827828
EPHX1	greenyellow	-0.008196914	0.957885101
MOGAT2	grey	0.43416629	0.003233837
PLEKHA5	grey	0.379541004	0.011054158
ACSL6	grey	-0.373035681	0.012632869
ELOVL2	grey	0.356836024	0.017420728
RPL41	grey	0.33882345	0.024461247
SETDB2	grey	-0.333202725	0.027093186
PSMB4	grey	0.324556241	0.031598868
PPP2CA	grey	0.324039629	0.031886584
GOT1L1	grey	0.321581738	0.033285253
RPLP2	grey	0.315787506	0.036783477
MGAM2	grey	0.315558174	0.036927914
STAB2	grey	0.314070631	0.037876133
NDST4	grey	-0.298352023	0.049168499
PLA2G6	grey	-0.292442298	0.054061403
PLA2G2A	grey	0.289829072	0.05634632
RPL26L1	grey	0.287126049	0.058790611
CIDEC	grey	0.271996996	0.074078657
SRD5A1	grey	0.270328136	0.075940914
B3GAT1	grey	0.266216897	0.080685706
ARNTL	grey	0.263117626	0.084413543
MOGAT3	grey	0.258245934	0.090543113
PPM1L	grey	-0.257157561	0.091958521
FOLH1	grey	0.257115561	0.092013482
SOAT2	grey	0.255076905	0.094711982
PSMD4	grey	0.24828388	0.104146301
DGAT2	grey	-0.246437931	0.106830294
CYP2C19	grey	-0.24641904	0.106858032
OMD	grey	0.244431853	0.109806666
PLA2G1B	grey	0.243521559	0.111177897
PGAM1	grey	0.241638589	0.114055623
POMC	grey	-0.240098928	0.11645038
PI4KA	grey	-0.239603563	0.1172289
PLAAT5	grey	0.238875795	0.118379796
HYAL2	grey	-0.237928694	0.119890311
SPTLC3	grey	0.236717826	0.121842634
IARS1	grey	0.233973303	0.126356277
ACOXL	grey	-0.228197443	0.136263492
KMT2D	grey	-0.226683216	0.138953918
LTC4S	grey	0.22458885	0.142739817
FABP1	grey	-0.218648542	0.153892415

HDC	grey	-0.21806149	0.155028275
NOS3	grey	0.2178465	0.155445781
SRD5A2	grey	0.216796923	0.157495846
SIN3A	grey	-0.215950713	0.159163011
PPP2R1B	grey	-0.212818355	0.165446235
SLC35B3	grey	0.212369475	0.166361176
RPL10L	grey	-0.209940321	0.171376129
PSMC1	grey	0.208081349	0.17528696
SETD1B	grey	0.205881534	0.179997249
MAN2C1	grey	-0.202923337	0.186473335
MBTPS1	grey	0.200802495	0.191217272
OGN	grey	0.199036016	0.19523339
LYVE1	grey	-0.198704684	0.195993267
LDHAL6A	grey	0.193557562	0.208066813
PNLIP	grey	0.19347559	0.208263206
TKTL2	grey	0.191711439	0.212521249
PPP2R1A	grey	-0.191317819	0.213479521
AANAT	grey	0.190635849	0.215146886
BAAT	grey	0.189793449	0.217218948
PSMD9	grey	0.189076907	0.218992288
ETHE1	grey	-0.187551216	0.222801476
PITPNM3	grey	-0.187511749	0.222900615
CYP2C9	grey	0.187443529	0.223072053
NOS2	grey	0.185879925	0.227026359
SI	grey	0.183130449	0.234095963
PNPLA5	grey	0.179872826	0.242664878
SULT1E1	grey	0.179819658	0.242806473
PIP5K1A	grey	0.179606205	0.24337549
UGT1A3	grey	0.178675306	0.245867614
PSMB6	grey	0.178387243	0.246642271
SLC44A5	grey	0.178262976	0.24697696
APOA2	grey	0.176387835	0.252064484
SLCO1B1	grey	-0.17444943	0.257397178
RPL39L	grey	0.174140835	0.258253052
CERS3	grey	0.174089573	0.25839541
LIPF	grey	0.173820064	0.259144712
PAPSS2	grey	0.173160782	0.260983789
GGT5	grey	0.172982979	0.261481258
DIO3	grey	0.171765103	0.264905696
AWAT2	grey	0.171593771	0.265389826
NEU3	grey	0.171011857	0.267038521
G6PC2	grey	0.170154454	0.269480079
UGT1A8	grey	0.169172347	0.272294833
CYP19A1	grey	0.168913876	0.273038831
SLC10A1	grey	0.168666822	0.273751219
MED26	grey	-0.168315316	0.274766904
ACBD7	grey	-0.161515703	0.294902436
CRLS1	grey	0.160914798	0.296726545
AIMP2	grey	-0.160860476	0.296891801
TAT	grey	-0.16062379	0.297612538
PLCD3	grey	0.160169578	0.298998817
POM121	grey	-0.158836949	0.303090004
B3GALT5	grey	0.157528111	0.307142893
NUP88	grey	-0.155227436	0.314350479
PLCH1	grey	0.154972539	0.315155566
COLGALT2	grey	0.154188754	0.317639304
INPP5E	grey	-0.152662025	0.322512755
RPL37	grey	-0.151071039	0.327641053

GK2	grey	0.148885725	0.334767715
B4GALNT2	grey	-0.147772915	0.338433473
MBTPS2	grey	0.146986448	0.34103913
PIK3C2B	grey	0.14497049	0.347774575
PIKFYVE	grey	0.144657145	0.348828751
RPL3L	grey	0.141945734	0.358032185
ACER1	grey	0.139692247	0.365792195
PLEKHA8	grey	-0.138720286	0.369170181
DUOX2	grey	-0.13850993	0.369903711
GPAT4	grey	0.137980265	0.371754564
PPT2	grey	0.137863546	0.37216317
KMT5C	grey	0.136113138	0.378323046
XIST	grey	0.135934603	0.378954711
PSMA8	grey	0.134181473	0.385190538
CHST11	grey	0.133484374	0.387686794
FITM1	grey	0.133306853	0.388323995
THRSP	grey	-0.133152061	0.388880117
PNLIPRP2	grey	0.131628706	0.394377942
PRDM7	grey	0.130078264	0.400019791
CHIT1	grey	-0.127859028	0.408176135
EP300	grey	-0.125650614	0.416386668
ADH7	grey	-0.125243271	0.417911298
GANC	grey	0.125121901	0.41836618
NCAN	grey	-0.124906185	0.419175355
LPL	grey	-0.124791321	0.419606583
CKM	grey	0.124648145	0.420144458
TPO	grey	0.124279975	0.421529359
NEU2	grey	-0.12348297	0.424536185
DGKZ	grey	-0.123191127	0.425640222
PSMD8	grey	-0.123055918	0.426152263
XYLT1	grey	0.122877791	0.426827366
STARD4	grey	-0.121066416	0.433726501
PNLIPRP1	grey	0.118474948	0.443704004
OSBPL5	grey	0.118388874	0.444037552
ALAS2	grey	-0.117980757	0.445620944
CA6	grey	0.117842857	0.446156661
ALOX12	grey	0.117769279	0.446442642
DGKK	grey	0.116461521	0.451542362
SMYD1	grey	-0.114874236	0.45777455
LCLAT1	grey	0.114419529	0.459568406
RPS23	grey	0.114202444	0.460426162
PRKACG	grey	0.113755884	0.462193336
AKR1C1	grey	-0.113749998	0.462216652
AOC3	grey	0.113654198	0.462596249
OLAH	grey	0.113219189	0.464322022
ARSH	grey	0.110899724	0.47358186
PITPNM1	grey	-0.109809773	0.477966751
DUOX1	grey	0.109584645	0.478875104
AHRR	grey	-0.108964205	0.481383164
CPS1	grey	0.108262176	0.484229327
GPC6	grey	-0.10783186	0.485978246
FUT10	grey	-0.10694889	0.489577164
ACSM4	grey	-0.105690991	0.494728041
HSD3B1	grey	0.105265479	0.496476733
FIG4	grey	-0.105150775	0.496948669
GALC	grey	0.102437913	0.508177156
RHD	grey	0.102219163	0.509088111
PIK3R3	grey	0.102021387	0.509912432

AOC2	grey	0.101827749	0.51072016
ACHE	grey	0.101137872	0.513603088
PLCZ1	grey	0.100834988	0.514871382
ASH1L	grey	-0.100218849	0.517456228
HSD17B6	grey	-0.100090513	0.517995442
AGK	grey	-0.099655986	0.519823218
FABP4	grey	-0.098655857	0.524042261
RPL22L1	grey	0.098201393	0.525964993
FABP12	grey	0.098177967	0.526064198
CHST5	grey	0.095887333	0.53580882
GYS2	grey	0.09578444	0.536248588
LDHAL6B	grey	0.095679825	0.536695893
ACSM6	grey	0.095510343	0.537420937
APOA5	grey	-0.095053469	0.539377795
B3GNT7	grey	-0.095044819	0.53941488
HS3ST4	grey	0.094644778	0.541131192
PLA2G4E	grey	-0.094186025	0.54310263
SDC3	grey	-0.093259972	0.547092707
MTMR7	grey	-0.093029675	0.548087152
GAD2	grey	-0.092706074	0.549485945
PLIN1	grey	0.092474704	0.550487102
FUT9	grey	-0.092128708	0.55198587
RPL28	grey	-0.091731086	0.553710646
PFKFB1	grey	0.086985265	0.574490646
ACSM1	grey	-0.086693472	0.575779804
PRKACA	grey	-0.086605564	0.576168443
TECRL	grey	0.086128664	0.578278889
ABCB11	grey	0.086076099	0.57851172
PYGM	grey	0.084016532	0.587667625
RPS4Y1	grey	-0.083887908	0.588241567
SLC37A2	grey	0.083555	0.589728214
SMPD4	grey	-0.083184354	0.591385346
TSHB	grey	-0.082813547	0.593045255
PCYT1B	grey	0.082496989	0.594463947
INPP5B	grey	-0.081603639	0.598475643
HMGCLL1	grey	0.081102517	0.600731158
CYP2B6	grey	0.080265109	0.604508511
ADH4	grey	0.080170963	0.604933825
MED4	grey	0.079709652	0.607019701
LCT	grey	-0.078955788	0.610435039
PSMD5	grey	0.078062813	0.614491206
IPPK	grey	-0.077841888	0.615496478
PLA2G4D	grey	0.077279384	0.618059168
CEPT1	grey	0.077268373	0.618109377
CSNK2A2	grey	0.076459882	0.62180073
NEU1	grey	0.074810079	0.629361723
HAO1	grey	0.073996042	0.633106331
SREBF2	grey	0.073635276	0.634768781
IPMK	grey	0.07361753	0.634850602
CYP24A1	grey	-0.071567184	0.644332864
POM121C	grey	0.071410332	0.645060586
HS3ST6	grey	0.071322887	0.645466435
CDO1	grey	0.071251872	0.645796103
GNMT	grey	-0.07018548	0.650754572
GCK	grey	0.066912288	0.666066655
PIAS4	grey	-0.066670322	0.667204019
GLUD2	grey	-0.06617174	0.669549928
PEMT	grey	-0.064955118	0.675287359

SUV39H2	grey	0.064147039	0.679108251
UGT2B15	grey	-0.061567832	0.691356543
FBP2	grey	-0.061567249	0.691359323
PI4K2B	grey	0.061453789	0.691899946
HPSE2	grey	-0.060061333	0.698547119
SREBF1	grey	0.05727696	0.711905488
BMX	grey	0.057109438	0.712711967
CDIPT	grey	0.055069067	0.722559271
FUT7	grey	0.054241448	0.726566321
PSMB11	grey	0.053830656	0.728557926
UXS1	grey	-0.053264126	0.731307475
RPL6	grey	0.052933158	0.732915307
CHIA	grey	-0.052490705	0.735066502
UGT1A4	grey	-0.051138787	0.741651838
KMT5B	grey	-0.050450616	0.745011041
MED17	grey	0.049756187	0.748405542
FABP9	grey	0.048799265	0.753090847
RPS4Y2	grey	-0.048723052	0.753464386
CERS1	grey	-0.047700624	0.758480865
MTMR8	grey	-0.047693567	0.758515523
UGP2	grey	0.047565215	0.759145982
PGAM2	grey	0.047177145	0.761053094
MTAP	grey	-0.046659839	0.763597493
RPS16	grey	-0.044993195	0.771811606
GPX6	grey	-0.044065736	0.776393385
NFYB	grey	0.043980655	0.776814075
BCHE	grey	0.043420439	0.779585684
BCAN	grey	0.042672926	0.783288125
MED16	grey	-0.041828688	0.787475336
UROC1	grey	0.040828056	0.792445892
RPS24	grey	0.040667413	0.793244635
SLC5A5	grey	-0.037861519	0.807229053
ANKRD1	grey	-0.037293209	0.810068879
PLA2G2F	grey	0.03626201	0.815227873
PYCR2	grey	-0.03588713	0.817105293
SLC6A11	grey	-0.034214174	0.825495733
NR1D1	grey	-0.033845389	0.827347941
ARSI	grey	-0.03352359	0.828964914
PHKG1	grey	-0.032054787	0.836354134
EHMT1	grey	0.032005258	0.836603545
PRPS1L1	grey	-0.031950512	0.836879252
RPS27L	grey	-0.031882606	0.837221261
AMY1C	grey	-0.031698599	0.838148159
SMPD3	grey	-0.03165259	0.838379951
RPL10	grey	0.030052687	0.846448581
INPP4A	grey	0.029995573	0.846736912
RAB14	grey	0.02995722	0.846930539
PDHA2	grey	0.029942873	0.847002973
HS3ST3B1	grey	0.029853881	0.847452303
VNN1	grey	0.029339236	0.850051716
CARNS1	grey	0.029311709	0.850190797
PSMB7	grey	-0.029061087	0.851457274
SLC6A7	grey	-0.028068214	0.856478189
DGAT2L6	grey	-0.027843068	0.857617527
UGT2B4	grey	-0.027438098	0.859667575
TAFAZZIN	grey	-0.027192493	0.86091133
CYP2U1	grey	0.026980699	0.861984129
B3GALT2	grey	-0.026760231	0.863101125

B3GALT1	grey	0.026502008	0.864409739
TYR	grey	-0.025653453	0.868712534
GAPDHS	grey	0.022846821	0.882970265
NDST3	grey	-0.021548798	0.889576892
PAFAH1B1	grey	0.021404479	0.890311911
CYP7B1	grey	-0.021277423	0.89095908
CPNE7	grey	0.021070679	0.892012293
DGKE	grey	0.020524818	0.894793956
GALE	grey	0.020326926	0.895802702
LALBA	grey	-0.020026082	0.897336559
SERINC5	grey	0.019662882	0.899188829
EZH1	grey	-0.017471718	0.910374354
PITPNM2	grey	-0.01540295	0.920950841
PPP1CB	grey	-0.014083199	0.927705173
AIMP1	grey	0.012452551	0.93605753
SUMF2	grey	0.011156297	0.94270197
ACAN	grey	-0.010878387	0.94412702
HKDC1	grey	0.010762471	0.944721457
DGKH	grey	-0.010210235	0.947553823
OTC	grey	-0.008778494	0.954899975
IDH1	grey	0.007967295	0.959063849
FUT5	grey	0.00728465	0.962568699
NCOA6	grey	-0.004798237	0.975339898
NHLRC1	grey	0.004118205	0.978834002
NOS1	grey	0.004049919	0.979184887
B3GAT2	grey	-0.00332553	0.982907375
SGPP2	grey	0.002939289	0.984892344
PLD4	grey	0.00255875	0.986848092
SLCO1A2	grey	-0.002317718	0.988086899
MIX23	grey	0.001277556	0.99343318
CYP2D6	grey	-0.001131979	0.994181455
GNE	grey	0.000553346	0.997155697
EPRS1	magenta	0.467824554	0.001364026
GNPAT	magenta	0.359471999	0.016550677
HAL	magenta	-0.345918522	0.021447034
ENOPH1	magenta	-0.298220638	0.049273245
ARG1	magenta	-0.274057278	0.071829445
IMPA1	magenta	0.256664702	0.092605081
OSBPL10	magenta	0.24931552	0.102668962
KERA	magenta	0.237805937	0.120087153
MBOAT2	magenta	-0.2260484	0.14009349
GNPDA2	magenta	-0.218486255	0.154205805
SLC44A4	magenta	-0.209378461	0.172551449
CYP7A1	magenta	0.208391713	0.174629607
ARF1	magenta	0.200280755	0.192397308
ACYP2	magenta	-0.194926187	0.20480691
FUT6	magenta	-0.187998718	0.221679493
MED18	magenta	-0.181210569	0.23912069
PLA2G2D	magenta	-0.178361796	0.246710782
JMJD7-PLA2G4B	magenta	-0.177866226	0.248047573
RPL8	magenta	0.175137513	0.255495642
KMT2E	magenta	-0.172461748	0.262943236
RPL24	magenta	0.170876962	0.267421676
PTGR2	magenta	-0.165652046	0.282542994
PPM1K	magenta	-0.152364574	0.323467686
AKR1D1	magenta	-0.14441651	0.349639639
PIK3R4	magenta	0.13186555	0.393520203
PNPLA6	magenta	-0.131179827	0.396006562

SMYD2	magenta	0.129461589	0.402276723
HSD17B7	magenta	-0.126688607	0.412515939
RPL9	magenta	-0.123133247	0.425859375
HYKK	magenta	-0.120213659	0.436995848
CSNK1G2	magenta	-0.118136916	0.44501472
GDPD1	magenta	0.117025076	0.449340845
RHCE	magenta	-0.111835354	0.469834884
CYB5RL	magenta	-0.108993572	0.481264297
DOT1L	magenta	-0.107318906	0.488067327
RPS3A	magenta	-0.097502872	0.52892703
MIGA1	magenta	-0.097287671	0.529841222
PIP4K2B	magenta	-0.097281949	0.529865542
HACD4	magenta	-0.081942111	0.5969543
RAB4A	magenta	0.061213817	0.693043889
FAU	magenta	0.06022214	0.697778325
GYG2	magenta	0.058982087	0.70371452
STARD6	magenta	-0.056622072	0.715059988
MED31	magenta	0.052986632	0.732655456
RPL37A	magenta	-0.048746066	0.753351583
RXRA	magenta	-0.04300666	0.781634544
MED7	magenta	-0.042462772	0.784329875
AHR	magenta	-0.04091606	0.792008412
PIK3C3	magenta	0.038189245	0.805592533
SIN3B	magenta	-0.033120546	0.830991117
SPTLC1	magenta	0.028523803	0.854173585
UBA52	magenta	0.02713659	0.86119447
SEC24B	magenta	0.022807933	0.883168081
PLA2G12A	magenta	-0.020219406	0.896350852
PSMD12	magenta	-0.019114864	0.901984645
HSPG2	magenta	0.017840184	0.908492141
PSMB8	pink	0.607247251	1.24E-05
PSMB9	pink	0.561011137	7.45E-05
STARD5	pink	0.450211129	0.002166239
GUSB	pink	0.406207046	0.006219671
ACSL5	pink	0.402238204	0.006795284
PSMB10	pink	0.387799794	0.009295702
TBXAS1	pink	0.382585421	0.010375386
IL4I1	pink	0.370100498	0.013405841
PIK3CG	pink	0.368891752	0.013735673
DSE	pink	0.361933138	0.015771811
UBE2L6	pink	0.361513706	0.015902307
HEXB	pink	0.357134876	0.017320184
GLCE	pink	-0.354394248	0.018260851
MAN2B1	pink	0.345184474	0.021743669
GLIPR1	pink	0.341309652	0.023367124
GM2A	pink	0.33920622	0.024290064
PPT1	pink	0.338982603	0.024389949
PSME4	pink	0.334297107	0.026563051
CD44	pink	0.331711191	0.027829849
INPP5D	pink	0.30637461	0.043105768
GBA	pink	0.305221088	0.043938005
BLVRA	pink	0.304779975	0.044259692
PLB1	pink	0.301086909	0.04702872
PSAP	pink	0.29766039	0.049721923
ALOX5AP	pink	0.293007048	0.053577534
LIPA	pink	0.288309058	0.057710601
SOAT1	pink	0.282082503	0.06357776
NAGK	pink	0.276276481	0.069467411

SLC25A15	pink	-0.270373171	0.075890181
DPEP2	pink	0.252235208	0.098574905
MTF1	pink	0.242696014	0.112432694
PIP4K2A	pink	0.242601084	0.112577671
TNFAIP8L2	pink	0.237525853	0.120537186
SDS	pink	0.235489892	0.123846838
NUP214	pink	-0.227231492	0.137975245
PLAAT4	pink	0.226317241	0.139610045
PLA2G2E	pink	0.22574362	0.140643063
HSD11B1L	pink	-0.217015777	0.157066753
LGMN	pink	-0.21168092	0.167771758
HK3	pink	0.203901873	0.184313023
AHCY	pink	-0.19596775	0.202350133
GYG1	pink	0.184240726	0.231223277
PSME2	pink	0.18149614	0.238368687
PLCB2	pink	0.178573622	0.246140873
MCAT	pink	-0.175995024	0.253139089
KYNU	pink	0.174676632	0.256768254
HPSE	pink	0.170741975	0.267805455
PI4K2A	pink	-0.163568833	0.288724698
ALOX15B	pink	-0.146239295	0.343525968
TKTL1	pink	-0.144596978	0.349031392
FITM2	pink	0.139608781	0.366081543
NPL	pink	0.138013041	0.371639874
LAP3	pink	0.133996997	0.385850209
GPX1	pink	0.127979679	0.407730266
HS3ST2	pink	-0.126453932	0.413389249
MAN2B2	pink	0.121161169	0.433364074
GLA	pink	0.12041527	0.43622167
SAT1	pink	-0.113549597	0.463010909
MPST	pink	-0.113304822	0.463982024
CHST12	pink	0.100862352	0.514756733
HPGDS	pink	0.094657841	0.541075107
UGCG	pink	-0.091553506	0.554481759
PIK3CD	pink	0.084102609	0.587283673
INPP5A	pink	-0.083069971	0.591897158
PLA2G7	pink	0.054502465	0.725301781
PSPH	pink	-0.050651445	0.744030242
DGKA	pink	0.043945802	0.776986425
CYB5R4	pink	0.042556987	0.783862798
TIGAR	pink	0.035538451	0.818852404
PIK3R5	pink	0.022893968	0.88273044
FDPS	pink	-0.017971509	0.907821421
DPEP3	pink	0.013172874	0.932367068
PLD3	pink	-0.00828466	0.957434683
ME2	pink	0.001302576	0.993304575
ADH1C	purple	-0.472042675	0.001216516
HS6ST1	purple	-0.415406011	0.005045528
PITPNB	purple	0.392717748	0.008367358
CYP39A1	purple	-0.392626493	0.008383828
DEGS2	purple	-0.392081497	0.00848277
ORMDL3	purple	-0.376649429	0.011733697
LIPH	purple	-0.365254143	0.014770237
ISYNA1	purple	-0.357645224	0.017149619
ALDH3B2	purple	-0.356619319	0.017493943
HS3ST5	purple	-0.3468927	0.02105861
SULT2B1	purple	-0.339811508	0.02402141
ACYP1	purple	-0.307604813	0.0422324

FUT2	purple	-0.278884194	0.066770871
ASAH2	purple	0.253110264	0.097372681
PNMT	purple	-0.248789402	0.103420358
CYP1A2	purple	-0.246305424	0.107024972
MED28	purple	-0.236834464	0.121653538
SLC44A1	purple	-0.23337859	0.127350672
CYP4B1	purple	-0.224769542	0.142410212
OXCT2	purple	-0.219787595	0.151705926
SPTSSB	purple	-0.210789915	0.169609903
CYP1A1	purple	-0.203294928	0.185650861
TH	purple	-0.200463806	0.19198271
PTGES	purple	-0.187819477	0.222128419
GPX2	purple	-0.183272834	0.233726205
B3GNT3	purple	-0.154948632	0.315231143
PRXL2B	purple	-0.15143421	0.32646596
NPAS2	purple	0.130038962	0.400163411
PFKFB3	purple	-0.121396165	0.432465951
PLPP5	purple	0.110545125	0.475006073
PTGS1	purple	-0.109234285	0.48029055
ALOX15	purple	0.074430283	0.631107665
PNLIPRP3	purple	-0.069701028	0.65301211
TGS1	purple	-0.066896064	0.666142893
HSD17B2	purple	0.066788133	0.666650156
AWAT1	purple	0.061732448	0.690572433
CHST6	purple	-0.057264932	0.711963383
CBS	purple	0.057218842	0.712185241
OSBPL3	purple	0.053324	0.731016726
PLA2G10	purple	-0.02906406	0.851442248
AKR1B1	purple	-0.027623065	0.858731119
CYP4F22	purple	-0.025851567	0.867707613
LIPI	purple	-0.024165422	0.87626692
B3GALT4	purple	-0.022009112	0.887233136
PSME1	purple	0.01842325	0.905514714
NTHL1	purple	-0.011876465	0.939009977
ELOVL1	purple	0.010299186	0.947097555
CERS6	purple	-0.006845725	0.964822608
PLOD2	red	0.771761781	8.64E-10
PLOD3	red	0.602357326	1.52E-05
COLGALT1	red	0.597554532	1.85E-05
SEC24D	red	0.596785964	1.91E-05
KYAT3	red	-0.596771944	1.91E-05
GPX8	red	0.595088531	2.04E-05
ASAH1	red	-0.593982363	2.13E-05
LGALS1	red	0.588265403	2.68E-05
PMM2	red	0.571506688	5.08E-05
CHSY3	red	0.561348953	7.36E-05
PIK3CA	red	0.555009241	9.23E-05
MORC2	red	0.539307772	0.000158438
ABCD1	red	0.530815054	0.000209901
TSTD1	red	-0.520460611	0.000292845
CHST14	red	0.518665353	0.000309916
TSPOAP1	red	-0.509368017	0.000413538
PEDS1	red	0.502260201	0.000512745
ALDH18A1	red	0.50191348	0.00051809
ESYT2	red	0.500443764	0.000541306
ENO2	red	0.498988813	0.000565204
SRM	red	0.49623484	0.000613044
FAAH2	red	-0.494754317	0.000640236

RPL36AL	red	-0.475705886	0.001100113
NCOR2	red	0.459921249	0.001683734
NCOA1	red	-0.457946617	0.001773315
PTGR1	red	-0.457338994	0.001801717
GFPT2	red	0.440044011	0.002798574
B4GALT7	red	0.439187023	0.002858637
RPL22	red	-0.439032648	0.002869577
PKM	red	0.432949799	0.003331002
PRDM2	red	0.42559625	0.003974745
DGKG	red	0.423477653	0.004179334
B3GNT2	red	-0.416410612	0.004929836
ITPKA	red	0.408548389	0.005900334
B4GALNT1	red	0.408149962	0.005953649
CHPF	red	0.406209512	0.006219327
SUCLA2	red	-0.406196162	0.00622119
SP1	red	0.405962015	0.006253942
CMAS	red	0.404697369	0.006433435
RPS6	red	-0.394965843	0.007970341
GYS1	red	0.392716259	0.008367627
CPOX	red	0.384481708	0.009970919
ACER3	red	0.358060957	0.017011727
CHPF2	red	0.355298032	0.017945994
NSD1	red	0.352584189	0.018905459
SLC44A2	red	-0.348354898	0.020486698
GRHL1	red	-0.343096923	0.02260613
HEXA	red	0.343011826	0.022641887
UBC	red	0.341176641	0.023424604
PSMD2	red	0.339658851	0.024088931
EXT1	red	0.332249054	0.027562305
RPL3	red	-0.332008682	0.027681606
KMT5A	red	0.330026012	0.028682097
MED14	red	0.329328488	0.029041139
ADH5	red	-0.322472816	0.03277245
TPR	red	0.319828617	0.034313468
XYLT2	red	0.318457225	0.035135905
MAT1A	red	0.315855067	0.036741014
GCKR	red	0.312812981	0.038693293
PLAAT1	red	0.306370965	0.043108377
B3GAT3	red	0.304485343	0.044475621
LYPLA1	red	-0.303908844	0.044900601
DNMT3B	red	0.298174435	0.049310122
AFMID	red	-0.298172781	0.049311443
MED15	red	0.296494594	0.050666142
MED24	red	0.296446143	0.050705695
GPC1	red	0.288874225	0.057200281
RPS29	red	-0.287064614	0.058847136
GAA	red	0.285233219	0.060552219
LIPT1	red	-0.270082699	0.076217871
PIK3R6	red	0.27008008	0.07622083
PSMA7	red	0.266259382	0.080635516
PYCR3	red	0.258196177	0.090607451
SLC7A5	red	0.245272304	0.108552114
DHRS7B	red	-0.241644217	0.114046938
P4HA3	red	0.241585804	0.114137099
SETD1A	red	0.237825494	0.120055778
UGT1A5	red	-0.236978058	0.121421046
NAALAD2	red	-0.228543203	0.135654629
CHST3	red	0.227578233	0.137358961

PSMC6	red	-0.2253395	0.141374225
RPE	red	-0.225024327	0.141946409
KYAT1	red	-0.217422874	0.156270858
PPP2CB	red	-0.216658973	0.157766756
CSAD	red	0.204504874	0.182990703
OCRL	red	0.187957313	0.22178314
DGKQ	red	0.182620127	0.235424489
RPS27	red	-0.170854086	0.267486687
PCYT1A	red	0.162410955	0.292198265
GLO1	red	-0.158261614	0.304867318
GLUL	red	-0.157398137	0.307547241
PLEKHA3	red	-0.1562054	0.311273707
SLC36A4	red	0.155831723	0.312447065
G6PC3	red	0.152492282	0.323057478
INMT	red	-0.150411422	0.329782098
AAAS	red	0.143004197	0.354422035
ALOX12B	red	0.139505857	0.366438535
TMEM86B	red	0.136255281	0.377820586
B4GALT1	red	0.131536498	0.394712172
KMT2B	red	0.117374032	0.447980603
FAR1	red	0.10753015	0.487206441
ODC1	red	-0.104688492	0.498852997
B4GALT2	red	0.087323105	0.5729997
MTM1	red	0.052393022	0.735541701
SGSH	red	-0.051175737	0.741471606
MAT2B	red	-0.0496751	0.748802214
RPEL1	red	-0.04102516	0.791466143
NUP133	red	-0.031027251	0.841531761
PTEN	red	0.02557627	0.869104095
EEF1E1	red	-0.013438633	0.931005845
ACOT11	turquoise	-0.650813886	1.73E-06
L2HGDH	turquoise	-0.646559539	2.12E-06
DHRS11	turquoise	-0.603519913	1.45E-05
PDHA1	turquoise	-0.598820268	1.75E-05
HADH	turquoise	-0.584080425	3.15E-05
IVD	turquoise	-0.575191658	4.42E-05
SCD5	turquoise	-0.573519043	4.71E-05
DCXR	turquoise	-0.567639424	5.86E-05
DBT	turquoise	-0.566704958	6.06E-05
CA4	turquoise	-0.562625423	7.03E-05
AMT	turquoise	-0.561990466	7.20E-05
SPHK2	turquoise	-0.561532838	7.32E-05
SUOX	turquoise	-0.557215878	8.54E-05
PCCA	turquoise	-0.551973657	0.000102685
CAV1	turquoise	0.551835518	0.000103182
HPGD	turquoise	-0.551688283	0.000103714
ALDH6A1	turquoise	-0.541748631	0.00014593
ACAD8	turquoise	-0.539956394	0.000155023
ACAA1	turquoise	-0.533873351	0.000189845
DHDH	turquoise	-0.525034666	0.000253118
ACSF2	turquoise	-0.519611572	0.000300809
ACOT2	turquoise	-0.517137789	0.000325142
SUCLG1	turquoise	-0.513840433	0.000360331
NUP62	turquoise	0.498744115	0.000569315
DIO1	turquoise	-0.498209231	0.000578394
BPHL	turquoise	-0.497878964	0.000584065
SUCLG2	turquoise	-0.494646323	0.000642261
CBR4	turquoise	-0.489241535	0.000751242

REEP6	turquoise	-0.488314161	0.000771521
GPC5	turquoise	-0.488174744	0.000774611
ECHS1	turquoise	-0.485024174	0.000847469
NDST1	turquoise	0.483826074	0.000876743
SAT2	turquoise	-0.481805722	0.000928159
PSMC2	turquoise	0.479184165	0.000998879
ACOT12	turquoise	-0.472284976	0.001208492
PTGDS	turquoise	-0.470438881	0.00127084
FBP1	turquoise	-0.469969173	0.001287153
ALDOB	turquoise	-0.467547973	0.001374231
SLC25A20	turquoise	-0.466739926	0.001404436
GALT	turquoise	-0.465984401	0.001433209
DPEP1	turquoise	-0.464583682	0.001487942
BGN	turquoise	0.460258436	0.001668845
ARSF	turquoise	-0.446770844	0.002364408
GCAT	turquoise	-0.444493403	0.002504232
FMOD	turquoise	0.444458099	0.002506456
PLPP4	turquoise	-0.443106976	0.002592876
EPHX2	turquoise	-0.440810664	0.002745785
CD1D	turquoise	0.440432498	0.002771713
CHST15	turquoise	0.439491558	0.002837165
TYRP1	turquoise	-0.43939466	0.002843982
PPP1CC	turquoise	0.438225368	0.002927384
SGPP1	turquoise	-0.435275506	0.003147417
ALAD	turquoise	-0.434669536	0.003194374
PHGDH	turquoise	-0.432437134	0.003372712
UGT2A1	turquoise	-0.43233488	0.003381086
VCAN	turquoise	0.431909867	0.003416087
ABAT	turquoise	-0.426437447	0.003895976
SHPK	turquoise	-0.425904088	0.003945759
AQP7	turquoise	-0.425015817	0.004029906
MDH1	turquoise	-0.418705026	0.004674239
SCP2	turquoise	-0.414505298	0.005151259
MIOX	turquoise	-0.41414303	0.005194325
EPM2A	turquoise	-0.411181922	0.005558248
IYD	turquoise	-0.410048254	0.005703334
SLC51B	turquoise	-0.408684336	0.005882238
RARS1	turquoise	0.406796734	0.006137863
TREH	turquoise	-0.405659926	0.006296418
ALDH1B1	turquoise	-0.404307883	0.006489607
HMGCS2	turquoise	-0.402862692	0.006701765
FTCD	turquoise	-0.400708456	0.007029157
PGM2	turquoise	0.400428523	0.0070727
ACOX2	turquoise	-0.391565904	0.008577298
HPD	turquoise	-0.391346423	0.008617812
HIBCH	turquoise	-0.391261091	0.008633608
HK1	turquoise	0.389030185	0.009055513
G6PC1	turquoise	-0.387724749	0.009310527
FABP5	turquoise	0.383620068	0.010152992
IDNK	turquoise	-0.383151902	0.010253111
SDHB	turquoise	-0.381702685	0.010568416
LDHB	turquoise	-0.37924698	0.011121681
PSMA1	turquoise	0.37856512	0.01127963
SPHK1	turquoise	0.377736384	0.011474182
CYP4F2	turquoise	-0.37712576	0.01161936
PAH	turquoise	-0.376242706	0.011832086
PRKD2	turquoise	0.374827665	0.012179903
FUT3	turquoise	-0.374416831	0.012282507

ASS1	turquoise	-0.372464563	0.012780218
HOGA1	turquoise	-0.372302149	0.012822388
CYP27B1	turquoise	-0.372110347	0.012872341
NSD2	turquoise	-0.370734875	0.013235453
DAO	turquoise	-0.37064233	0.013260194
LPIN2	turquoise	-0.369314392	0.01361957
FADS1	turquoise	0.369036953	0.01369569
SEPHS2	turquoise	-0.366814935	0.014318505
PGM2L1	turquoise	0.364282689	0.015057465
HMGCL	turquoise	-0.363412276	0.015318831
CYP4A22	turquoise	-0.363049904	0.015428772
PCK2	turquoise	-0.35910791	0.016668616
TKFC	turquoise	-0.355883981	0.017744321
NEU4	turquoise	-0.354919164	0.01807742
ARG2	turquoise	-0.354573934	0.018197884
ACADSB	turquoise	-0.353771285	0.018480578
ACO1	turquoise	-0.349522734	0.020039348
FAAH	turquoise	-0.349465971	0.0200609
NUP155	turquoise	0.348914374	0.020271349
HS6ST2	turquoise	-0.348778469	0.020323485
GCDH	turquoise	-0.347841776	0.020685891
GLYCTK	turquoise	-0.34739203	0.020861817
ASNS	turquoise	0.344078662	0.022197027
GK	turquoise	-0.339925133	0.023971256
ACADM	turquoise	-0.337935834	0.024862088
SUV39H1	turquoise	0.337718939	0.024960865
ADH6	turquoise	-0.337042881	0.025270854
GALM	turquoise	-0.33401007	0.026701254
GDPD5	turquoise	0.333556094	0.026921055
PIPOX	turquoise	-0.332602868	0.027387479
ACSS2	turquoise	-0.330654882	0.02836156
CYP4F3	turquoise	-0.329253088	0.029080172
MED13	turquoise	0.327448207	0.030027596
CPT2	turquoise	-0.324788198	0.031470384
NFYC	turquoise	-0.322192733	0.032932927
GLDC	turquoise	-0.321880349	0.033112675
MED10	turquoise	0.32170487	0.033214001
AGXT	turquoise	-0.320137571	0.03413039
PGLS	turquoise	0.319710053	0.03438394
CCNC	turquoise	-0.31943665	0.0345469
UBE2I	turquoise	0.31867808	0.035002371
ALDH9A1	turquoise	-0.3175803	0.035670253
OAZ3	turquoise	0.317140113	0.035940986
ENPP6	turquoise	-0.315653565	0.036867778
SETDB1	turquoise	0.313432403	0.038289036
NDST2	turquoise	0.313292611	0.038379966
CNDP1	turquoise	-0.313229992	0.038420755
QDPR	turquoise	-0.309659696	0.040805768
HAO2	turquoise	-0.308976406	0.041275718
RPL38	turquoise	0.306748307	0.042838925
FHL2	turquoise	0.306437907	0.043060475
ACAT1	turquoise	-0.305845734	0.043485731
ALOX5	turquoise	0.30419788	0.04468712
GRHPR	turquoise	-0.302124669	0.0462368
SEM1	turquoise	0.301975447	0.046350001
SLC25A21	turquoise	-0.301791876	0.046489569
RPL36A	turquoise	0.299693555	0.04810925
HSD17B10	turquoise	-0.298206221	0.049284749

G6PD	turquoise	0.29766398	0.049719037
SORD	turquoise	-0.296467428	0.050688315
TST	turquoise	-0.295769704	0.051260518
YWHAH	turquoise	0.295742695	0.051282772
RPS19	turquoise	0.294327906	0.052459443
PSMD3	turquoise	0.290849195	0.055445311
ALDH7A1	turquoise	-0.287744857	0.05822368
PSMA6	turquoise	0.286431697	0.059432002
PCK1	turquoise	-0.282781276	0.062896574
NCOA2	turquoise	-0.282346295	0.063319921
ALB	turquoise	-0.282042166	0.06361726
AADAT	turquoise	-0.281278895	0.06436839
BDH2	turquoise	-0.279808024	0.065835747
ADPGK	turquoise	0.279359136	0.066288815
AGPAT3	turquoise	-0.278617039	0.067043247
CALM1	turquoise	-0.271305667	0.074845689
SLC37A1	turquoise	0.270960684	0.075230779
SLC25A11	turquoise	-0.270215566	0.076067842
AOX1	turquoise	-0.269448144	0.076937598
IDS	turquoise	0.265539244	0.081489583
RGN	turquoise	-0.264397963	0.082857586
CTH	turquoise	-0.264037025	0.083293939
CREBBP	turquoise	0.26183813	0.085991146
ALDH5A1	turquoise	-0.260313207	0.087901234
GDPD3	turquoise	-0.259573803	0.088839187
PLA2R1	turquoise	-0.258880485	0.089725722
SDSL	turquoise	-0.258487173	0.090231687
RETSAT	turquoise	-0.257300562	0.091771579
THEM5	turquoise	0.255654556	0.093941236
ARSD	turquoise	-0.255350018	0.094346968
MOGAT1	turquoise	-0.252591639	0.098083839
NUP205	turquoise	0.249247238	0.102766245
MCCC2	turquoise	-0.246347728	0.106962791
MMUT	turquoise	-0.245667406	0.10796613
ADI1	turquoise	-0.245117491	0.108782381
PCYT2	turquoise	-0.241756828	0.113873273
TSPO	turquoise	0.237732482	0.120205057
SEC23A	turquoise	0.237043914	0.12131453
SLC25A10	turquoise	-0.234980784	0.124685023
ACSBG2	turquoise	-0.234742049	0.125079538
CPNE6	turquoise	-0.230383449	0.132448091
IDUA	turquoise	0.228547793	0.135646559
ACOT6	turquoise	-0.223105961	0.145466145
ACOT13	turquoise	-0.222072645	0.147388511
G0S2	turquoise	-0.221764567	0.147965259
LRTOMT	turquoise	-0.219585615	0.152091967
MCCC1	turquoise	-0.218208385	0.154743481
ETNK2	turquoise	-0.216366288	0.158342665
ETNPPL	turquoise	-0.214936047	0.161178982
PLCH2	turquoise	-0.21475942	0.161531801
ALDH8A1	turquoise	-0.213979012	0.163097422
PPARA	turquoise	-0.213531346	0.164000473
PON3	turquoise	-0.209955914	0.171343593
ECI2	turquoise	-0.209550859	0.172190203
RENBP	turquoise	-0.206503456	0.178656484
CYP2R1	turquoise	0.20583745	0.180092559
GALK1	turquoise	0.205577306	0.180655735
PLEKHA4	turquoise	0.204600511	0.182781604

FAR2	turquoise	0.202895241	0.186535629
NUP42	turquoise	0.198517108	0.19642438
AASS	turquoise	-0.197008317	0.199916447
UBB	turquoise	-0.195293331	0.203938538
NAGS	turquoise	-0.193391883	0.208463888
GSS	turquoise	-0.191699576	0.212550087
CAMKMT	turquoise	-0.191662317	0.212640673
CYP2E1	turquoise	-0.191489303	0.213061673
SLC25A44	turquoise	-0.187486742	0.222963447
PXMP2	turquoise	-0.187215672	0.22364532
IDH3G	turquoise	-0.186700079	0.224946251
MCEE	turquoise	-0.184534173	0.230468076
ITPKC	turquoise	0.182971981	0.234507955
AGPAT4	turquoise	-0.180277766	0.241588304
PRDM6	turquoise	0.17960414	0.243380999
VAPB	turquoise	-0.175217215	0.255275993
ENO3	turquoise	-0.174859995	0.256261435
ACAD10	turquoise	-0.172634244	0.262458808
NAGLU	turquoise	-0.172054782	0.264088487
GADL1	turquoise	-0.17005525	0.269763526
CYP8B1	turquoise	-0.168490178	0.274261326
ACOX1	turquoise	-0.167568918	0.276931852
ESYT1	turquoise	0.166253678	0.280773933
GCSH	turquoise	-0.160094245	0.29922914
ME1	turquoise	0.155485827	0.313535689
IDO1	turquoise	0.15358723	0.319553838
GPX5	turquoise	-0.149275195	0.333490592
PLAAT2	turquoise	-0.1483191	0.33663117
NCAPH2	turquoise	0.147722056	0.338601602
XYLB	turquoise	-0.145005833	0.347655794
GPT2	turquoise	-0.144172663	0.350462526
DBH	turquoise	-0.140479513	0.363069783
KMT2A	turquoise	0.136103594	0.378356797
ACSM3	turquoise	-0.135256822	0.381358423
HAGH	turquoise	-0.133455199	0.387791475
DBI	turquoise	-0.128460882	0.405954766
CYP3A4	turquoise	-0.125317396	0.417633622
MED12	turquoise	-0.110577638	0.474875393
AHCYL1	turquoise	-0.106834787	0.490043244
PSAT1	turquoise	-0.102646651	0.507308663
PSMC5	turquoise	0.098284642	0.525612526
GC	turquoise	-0.088160215	0.569313031
OSBPL6	turquoise	-0.07808384	0.614395562
SARDH	turquoise	-0.075308206	0.627074826
STARD7	turquoise	-0.075292304	0.627147779
AGPAT1	turquoise	0.061002333	0.694052588
RANBP2	turquoise	-0.056821163	0.714100497
RPS15	turquoise	0.053139679	0.731911902
RPL35	turquoise	0.049414457	0.750077711
ENPP7	turquoise	-0.040913753	0.792019879
RDH16	turquoise	0.02484933	0.872793485
ACOT7	turquoise	-0.024545024	0.874338709
TPTE	turquoise	-0.02024599	0.896215319
PC	turquoise	-0.018406167	0.905601928
HSD17B13	turquoise	-0.016835346	0.913626235
ENPP1	turquoise	0.016124864	0.917258464
ECHDC1	turquoise	0.001498994	0.992294987
SEC24A	yellow	0.766736319	1.29E-09

PLCD1	yellow	-0.699382992	1.28E-07
LDHA	yellow	0.667688575	7.39E-07
PDHB	yellow	-0.655111837	1.40E-06
LPGAT1	yellow	0.65434506	1.45E-06
FABP6	yellow	0.648670639	1.92E-06
HSD3B7	yellow	0.639060589	3.03E-06
GMPPA	yellow	0.63768047	3.23E-06
ANGPTL4	yellow	0.636615036	3.40E-06
HK2	yellow	0.631149585	4.36E-06
NNMT	yellow	0.630007906	4.60E-06
SLC27A5	yellow	-0.604210601	1.41E-05
FDFT1	yellow	-0.603814951	1.43E-05
FABP7	yellow	0.595688044	1.99E-05
GAPDH	yellow	0.594986142	2.05E-05
MECOM	yellow	-0.592125757	2.30E-05
GLS2	yellow	-0.587809749	2.72E-05
ACLY	yellow	0.569240394	5.52E-05
MED13L	yellow	0.56684062	6.03E-05
TBL1XR1	yellow	0.566051674	6.21E-05
DEGS1	yellow	0.557397866	8.48E-05
TRIB3	yellow	0.556694279	8.70E-05
PLOD1	yellow	0.551596435	0.000104046
TECR	yellow	-0.548752979	0.000114847
PFKP	yellow	0.548209561	0.000117024
SLC2A1	yellow	0.545673823	0.000127683
ALDOA	yellow	0.545563098	0.000128168
BCKDK	yellow	0.545326261	0.000129211
SCAP	yellow	-0.526518052	0.000241322
P4HA2	yellow	0.526402019	0.000242227
ARNT2	yellow	-0.524351143	0.000258727
FUT11	yellow	0.520199837	0.000295271
GAL3ST1	yellow	0.518778632	0.000308812
LYPLA2	yellow	-0.516155692	0.000335284
PIK3C2G	yellow	-0.515751447	0.000339541
DARS1	yellow	0.51276856	0.000372488
SPTLC2	yellow	-0.512516681	0.000375397
MED22	yellow	-0.512055495	0.000380778
PSMD11	yellow	0.510477798	0.000399714
HACL1	yellow	-0.50997318	0.000405948
TPI1	yellow	0.508953119	0.000418815
PCCB	yellow	-0.507127162	0.000442768
SHMT2	yellow	0.506210051	0.000455258
S100A10	yellow	0.503575768	0.000492912
PGM3	yellow	0.502681445	0.000506318
ABCA1	yellow	0.502356547	0.000511269
PLA1A	yellow	0.501730326	0.000520933
RUFY1	yellow	0.495738616	0.00062204
NBN	yellow	0.493567053	0.000662817
CGA	yellow	-0.492023025	0.000693249
ENPP3	yellow	0.488384011	0.000769976
ADH1A	yellow	-0.486666708	0.000808755
GMDS	yellow	-0.483276843	0.000890463
ACP6	yellow	-0.482948649	0.000898752
CSPG4	yellow	0.481830794	0.000927505
MED20	yellow	-0.481259216	0.000942522
HILPDA	yellow	0.481085808	0.00094712
APIP	yellow	-0.481024663	0.000948746
DGKD	yellow	0.478070378	0.001030352

SQOR	yellow	0.476800694	0.001067305
PPARD	yellow	0.471103682	0.001248065
SRD5A3	yellow	0.470217965	0.001278489
PYGL	yellow	0.465925828	0.001435462
PSMC3	yellow	0.465576906	0.001448945
ABCC3	yellow	0.459253777	0.001713555
GPC3	yellow	-0.455137398	0.001908029
PFKFB4	yellow	0.452311274	0.00205261
MTRR	yellow	0.450616145	0.002143903
NR1H4	yellow	0.449656864	0.00219714
RPL15	yellow	-0.441043306	0.002729941
SMARCD3	yellow	-0.439180065	0.00285913
QARS1	yellow	-0.436408075	0.003061277
PHKA2	yellow	0.436103648	0.003084225
MINPP1	yellow	0.436091363	0.003085154
INSIG2	yellow	0.435941069	0.003096542
LARS1	yellow	0.430710715	0.003516559
GFPT1	yellow	0.430645639	0.003522085
CHD9	yellow	0.423142023	0.004212575
ADH1B	yellow	-0.421700295	0.004358009
SCD	yellow	0.41532694	0.005054734
B4GALT5	yellow	0.41350535	0.005270894
GALNS	yellow	0.410638966	0.00562733
LIPG	yellow	-0.405126936	0.006371972
SETD2	yellow	-0.404881462	0.006407032
ABCB4	yellow	0.401183049	0.006955866
ABHD5	yellow	-0.396594654	0.007692943
PLIN2	yellow	0.394781035	0.008002353
MGLL	yellow	0.39375434	0.008182226
PHYKPL	yellow	0.393636864	0.008203028
PSTK	yellow	-0.393211803	0.008278676
MTMR2	yellow	0.391300083	0.008626387
NR1H3	yellow	0.389994658	0.008870986
OAT	yellow	-0.369472115	0.013576457
PGM1	yellow	0.369414475	0.013592199
PLA2G5	yellow	0.357907464	0.017062528
NMRAL1	yellow	0.35655573	0.017515476
METAP1	yellow	-0.35277597	0.018836265
ALDOC	yellow	0.352119535	0.019073993
MBOAT1	yellow	-0.348892411	0.020279767
MED21	yellow	-0.347764097	0.020716187
PTPN13	yellow	-0.340392875	0.023765714
GLB1	yellow	-0.339727525	0.024058536
MIF	yellow	0.335184818	0.026139399
MTMR1	yellow	-0.334643775	0.026396932
AMDHD2	yellow	0.328266877	0.029594737
MANBA	yellow	0.327032876	0.030249193
PPP1CA	yellow	-0.326916079	0.030311753
GBE1	yellow	0.324635109	0.031555133
IDO2	yellow	-0.322946564	0.03250248
STARD3NL	yellow	0.320962367	0.033645576
PGD	yellow	-0.316678311	0.036226825
STS	yellow	0.315544458	0.036936567
ACSL3	yellow	-0.307784937	0.042105742
SRR	yellow	-0.30730656	0.042442805
MED27	yellow	-0.30690318	0.042728731
PPP1R3C	yellow	0.30580589	0.043514466
PGK1	yellow	0.303568376	0.045153131

AZIN2	yellow	0.302562819	0.04590571
ITPKB	yellow	-0.300715214	0.047315022
B3GNT4	yellow	0.299922602	0.047930259
LPIN3	yellow	0.296879641	0.050352691
CYP2J2	yellow	0.296373264	0.050765237
PPARGC1B	yellow	0.294166995	0.052594638
HSD17B3	yellow	0.292688356	0.053850154
PLCB4	yellow	0.289378003	0.056748447
ENO1	yellow	0.286641667	0.05923746
RIMKLA	yellow	0.28497321	0.060797462
PLCB1	yellow	0.283799601	0.061914304
KMO	yellow	0.283745092	0.06196657
PTPRG	yellow	0.282374895	0.063292016
PGAM4	yellow	0.282373594	0.063293286
ADSS2	yellow	-0.276760697	0.068960292
PSMD6	yellow	-0.261305581	0.086654498
ERP29	yellow	-0.254885494	0.094968453
PAPSS1	yellow	-0.254195625	0.095897251
ARSA	yellow	0.253438029	0.09692529
ALDH3A1	yellow	0.251432665	0.099687516
MTMR14	yellow	-0.247953556	0.104622759
HS3ST1	yellow	-0.243769627	0.110802933
SLC6A8	yellow	0.236697004	0.121876413
MVK	yellow	0.231979796	0.129712651
PSMC4	yellow	-0.228009787	0.136594792
PSMA3	yellow	-0.227111384	0.138189199
PSME3	yellow	-0.225357279	0.141341999
INPP5K	yellow	0.223239578	0.145218924
CD36	yellow	0.201491299	0.189667253
ACSL4	yellow	-0.191173288	0.213832134
ALDH3B1	yellow	0.184474412	0.230621736
SLC45A2	yellow	0.182197274	0.236529196
PIP5KL1	yellow	-0.179958326	0.242437298
PAFAH1B2	yellow	-0.179646376	0.243268334
LPCAT1	yellow	0.175656248	0.254068341
FDXR	yellow	0.175496663	0.25450687
PLPP6	yellow	-0.172770364	0.262076958
SBF1	yellow	0.172343736	0.263274992
GPI	yellow	0.171578804	0.265432146
TPH1	yellow	0.171233144	0.266410765
ABCC1	yellow	0.162424362	0.292157889
AMY1B	yellow	-0.144372791	0.349787086
HYI	yellow	0.138265977	0.370755491
HGSNAT	yellow	-0.123053653	0.426160845
LCAT	yellow	0.121937417	0.430401322
AMY1A	yellow	-0.10319773	0.505019411
OCA2	yellow	0.102639356	0.507339
NANP	yellow	-0.083211459	0.59126409
DCT	yellow	0.068652642	0.657908035
