# Supplementary material for: Comprehensive analyses of A 12-metabolism-associated gene signature and its connection with tumor metastases in clear cell renal cell carcinoma
Source: BMC Cancer. 2023 Mar 23;23:264. doi: 10.1186/s12885-023-10740-6 (PMC10035225; doi:10.1186/s12885-023-10740-6)
Supplement: Supplementary file 3 — Additional file 3: Supplementary Figure 1. WGCNA construction and identification of modules associated with the clinical traits of ccRCC based on the GSE105261 cohort. Supplementary Figure 2. Construction and validation of the MAPS. Supplementary Figure 3. Relationship between risk scores and clinicopathological features. Supplementary Figure 4. Infiltration of immune cells and drug susceptibility analyses of the MAPS. Supplementary Figure 5. Analysis of the association between risk score and immune infiltration profiles. Supplementary Figure 6. Expression ofthe 12 genes in the MAPS in ccRCC and normal kidney tissue in TCGA-KIRC dataset. [file 12885_2023_10740_MOESM3_ESM.pdf]

Supplementary Figure 1: WGCNA construction and identification of modules associated with the clinical traits of ccRCC based on the GSE105261 cohort.

A

The connection between red module and the other modules

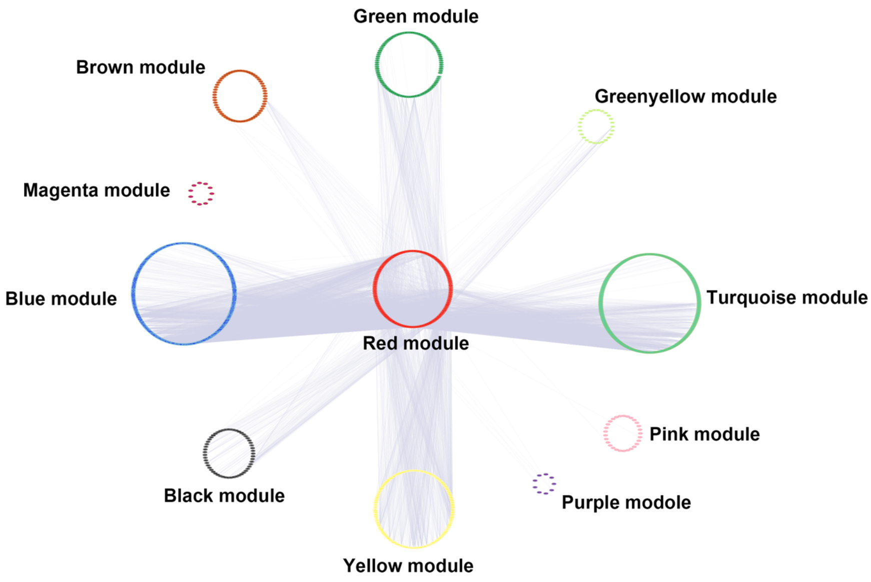

The network of edges and nodes list files of all modules generated via the "WGCNA" R package was exported into Cytoscape. Visualization of the network showed the connectivity between the red module and the other modules. Due to the number of nodes and edges being too much (nodes had 1049 and the edges had 22660), we only retained the edges where the weight value was greater than 0.065.

Supplementary Figure 2: Construction and validation of the MAPS

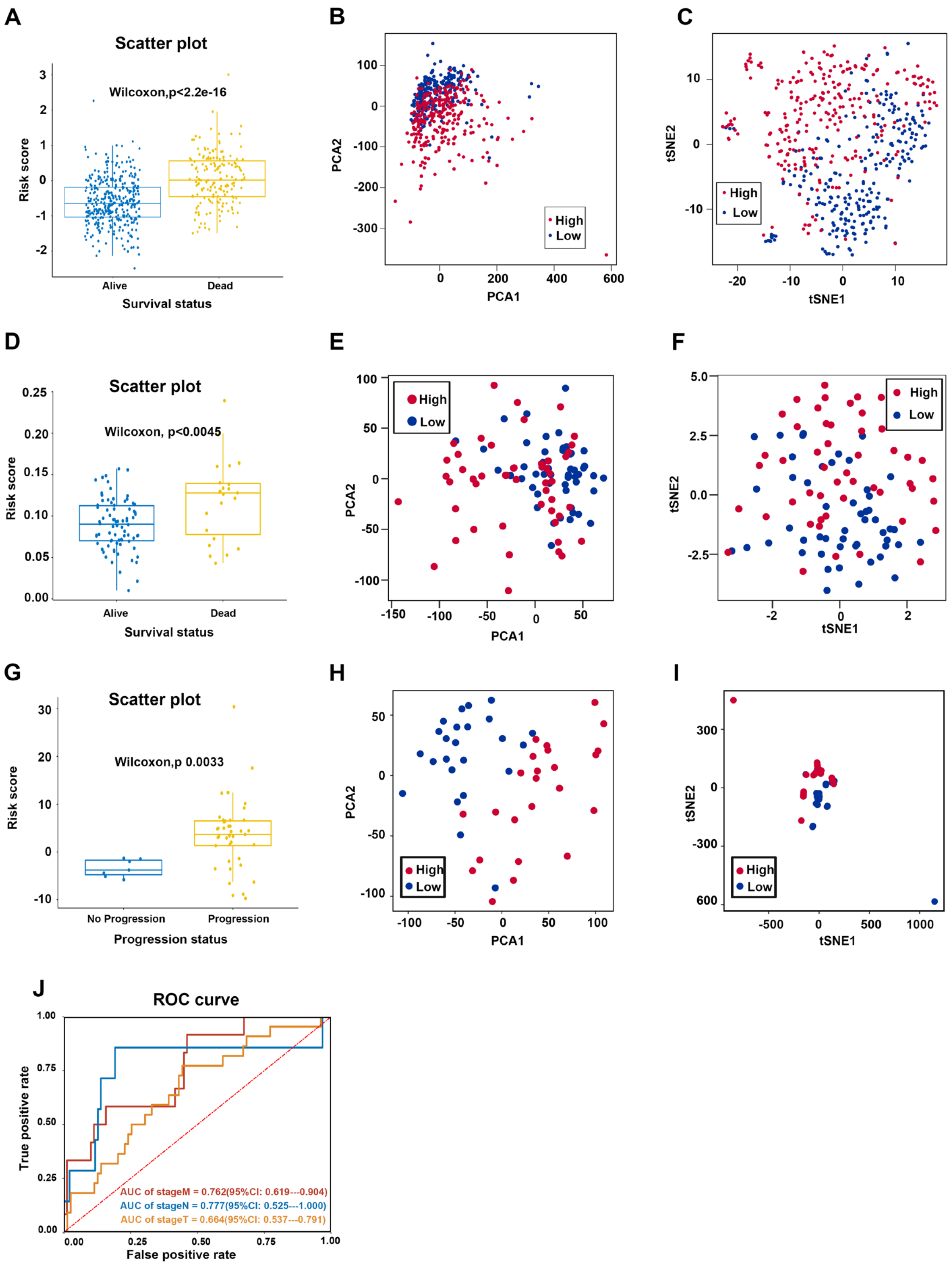

Comparison of the risk scores between patients end with death and alive patients based on TCGA-KIRC cohort (**A**) and E-MTAB-1980 (**D**). Comparison of the risk scores between the patients with progressed ccRCC and without progressed ccRCC based on the GSE22541 cohort(**G**). Principal component analysis (PCA) plot of the TCGA -KIRC(**B**), E-MTAB-1980(**E**), and GSE22541(**H**) cohorts. The t-distributed stochastic neighbor embedding (t-SNE) plot of the TCGA -KIRC(**C**), E-MTAB-1980(**F**), and GSE22541(**I**) cohorts. (**J**) The ROC curve of the prognostic model for predicting the tumor progresses from stage T1 or T2 to stage T3 or T4, the emergence of lymph node metastasis, and remote metastasis based on the E-MTAB-1980 cohort. *P* was calculated using Wilcoxon signed-rank test (**A**, **D,** and **G**).

Supplementary Figure 3: Relationship between risk scores and clinicopathological features

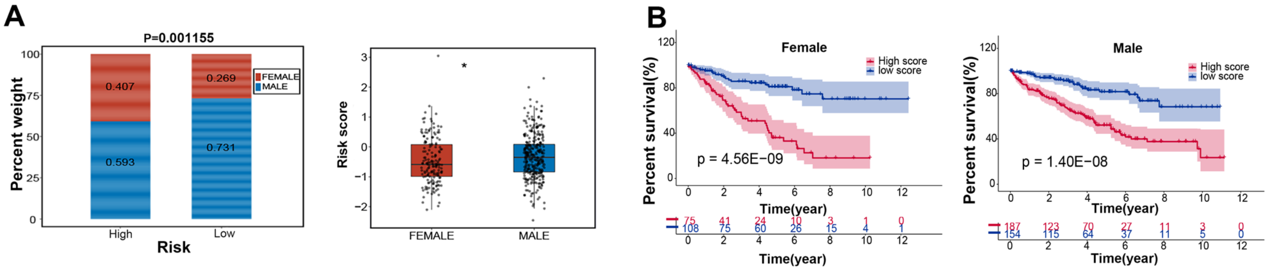

(**A**)The plots showed the association of risk score with gender based on TCGA-KIRC cohort. (**B**) Kaplan-Meier analysis of the MAPS regarding the stratified gender based on TCGA-KIRC cohort. *P* was calculated using the χ^2 test (the left panel of **A**), one-way ANOVA with Tukey's multiple comparisons tests (the right panels of **A**), and log-rank test (**B**). For all the panels, *P <0.05.

Supplementary Figure 4: Infiltration of immune cells and drug susceptibility analysis of the MAPS

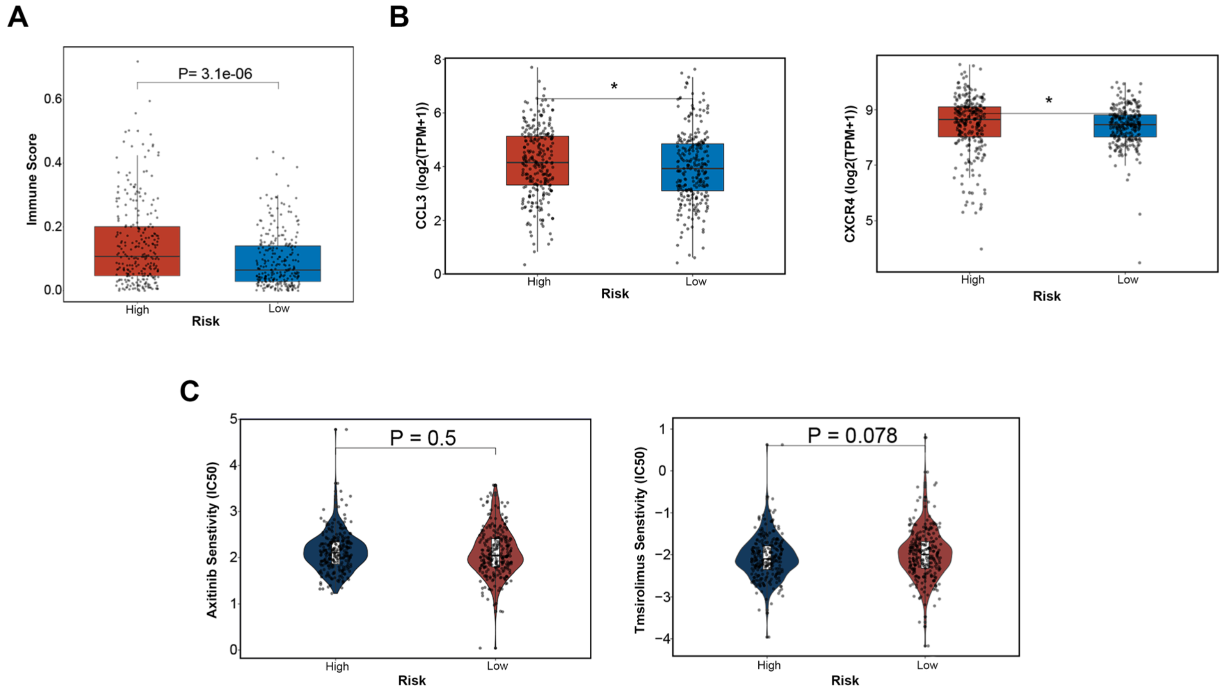

(**A**) Comparison of immune scores from the xCell algorithm between the high and the low-risk subgroups. (**B**) Comparison of the expression of *CCL3*, *CXCR4* between the high-risk subgroup and the low-risk subgroup of the MAPS based on the TCGA-KIRC cohort. The gene-level transcription estimates were shown in a form of log2 (TPM + 1). (**C**) Comparison of drug sensitivity of Axitinib and Tmsirolimus between the high-risk subgroup and the low-risk subgroup of the MAPS. *P* was calculated via a two-tailed Mann-Whitney test (**A, B,** and **C**). For all the panels, **P* <0.05.

Supplementary Figure 5: Analysis of the association between risk score and immune infiltration profiles

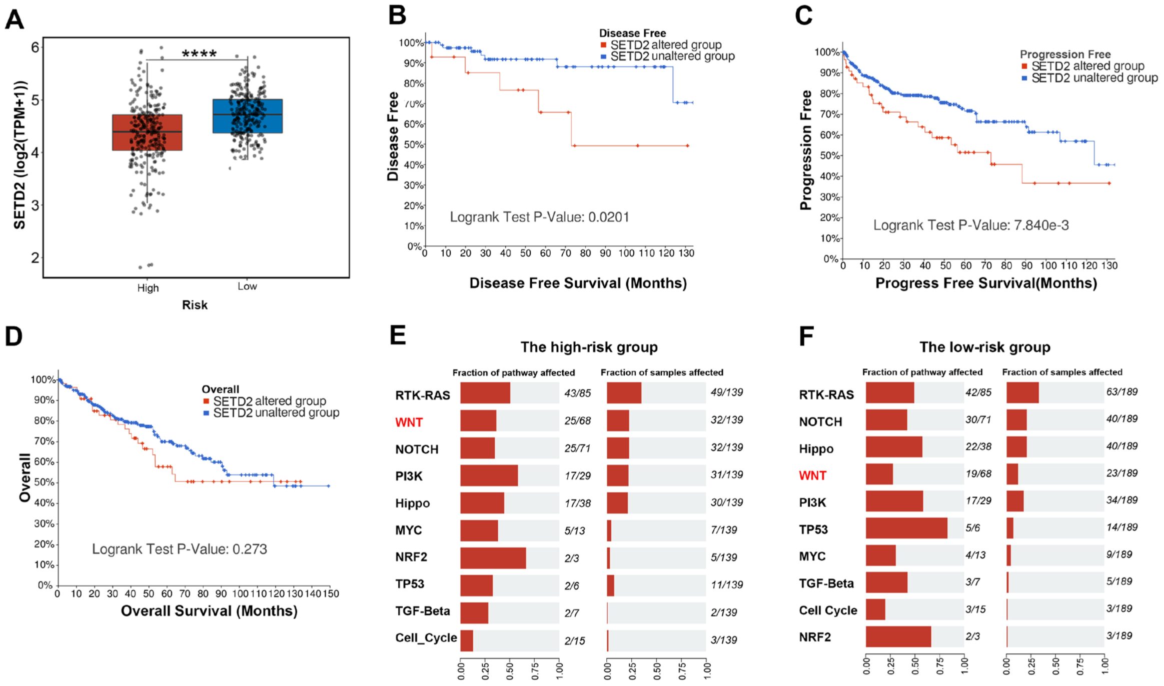

(**A**) Comparison of the expression of *SETD2* between the high-risk subgroup and the low-risk subgroup of the MAPS based on the TCGA-KIRC cohort. The gene-level transcription estimates were shown in a form of log2 (TPM + 1). The Kaplan-Meier analysis regarding PFS (**B**), DFS (**C**), and OS (**D**) of the stratified SETD2 subgroups. The plots are derived from the cbioportal website(www.cbioportal.org). Top 10 signaling pathways enriched by mutation genes in the high-risk subgroup(**E**) and the low-risk subgroup(**F**).
P was calculated using a two-tailed Mann-Whitney test(**A**), and log-rank test (**B, C,** and **D**). For all the panels, **P* <0.05, ***P* <0.01, ****P* <0.001, *****P* <0.0001, "ns" means no significance.

Supplementary Figure 6: Expression of the 12 genes in the MAPS in ccRCC and normal kidney tissue in TCGA-KIRC dataset.

A

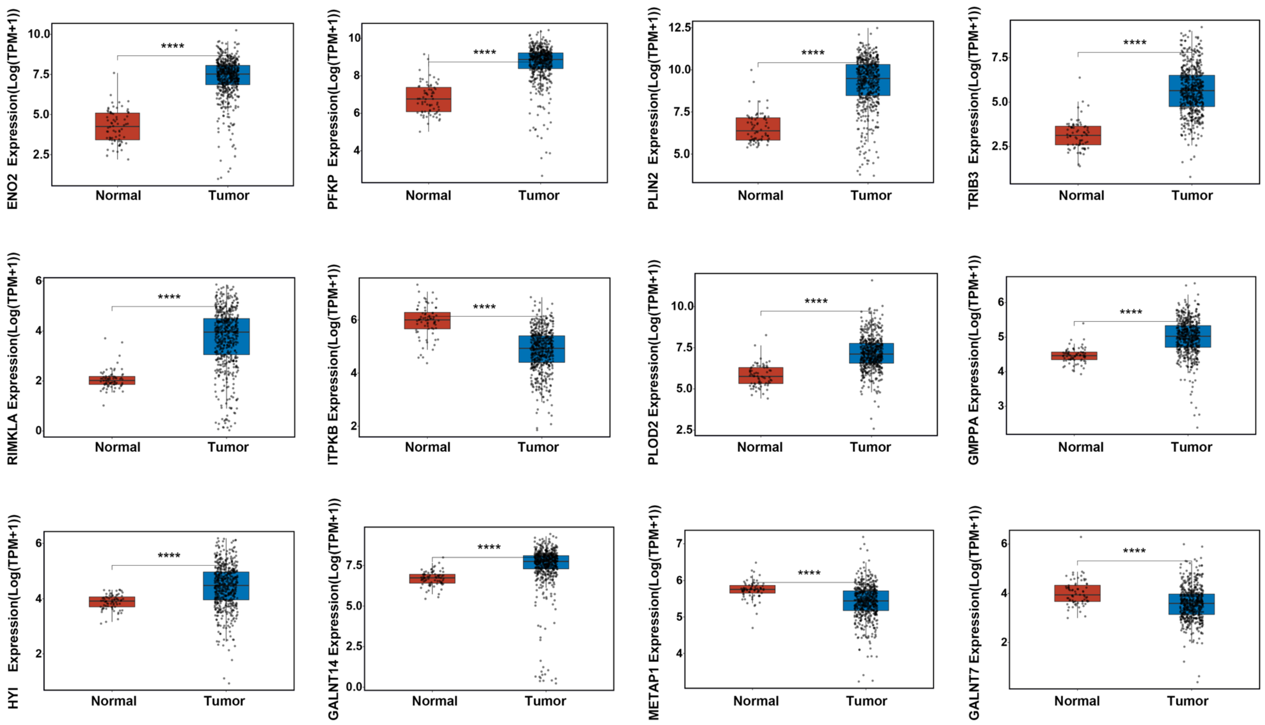

(**A**) The box plots of *ENO2*, *PFKP*, *PLIN2*, *TRIB3*, *RIMKLA*, *ITPKB*, *PLOD2*, *GMPPA*, *HYI*, *GALNT14*, *METAP1* and *GALNT7* in ccRCC tumor tissues and kidney normal tissues from TCGA-KIRC cohort. The gene-level transcription estimates were shown in a form of log2 (TPM + 1). *P* was calculated using a two-tailed Mann-Whitney test (**A**). For all the panels, **P* <0.05, ***P* <0.01, ****P* <0.001, *****P* <0.0001, "ns" means no significance.
