# Supplementary material for: Comprehensive analyses of A 12-metabolism-associated gene signature and its connection with tumor metastases in clear cell renal cell carcinoma
Source: BMC Cancer. 2023 Mar 23;23:264. doi: 10.1186/s12885-023-10740-6 (PMC10035225; doi:10.1186/s12885-023-10740-6)
Supplement: Supplementary file 4 — Additional file 4: Supplementary Table S3. The genes from the red module and their module menbership and trait significance value. [file 12885_2023_10740_MOESM4_ESM.pdf]

Supplementary Table S3: the genes from the red module and their module menbership and trait significance value

Gene ID	Module Membership of Gene	Trait Significance of Gene
PLOD2	0.762326228	0.771761781
SEC24A	0.744931462	0.766736319
P4HA1	0.82311879	0.699314339
NDUFA4L2	0.934195305	0.694026766
LDHA	0.84231678	0.667688575
LPGAT1	0.618715331	0.65434506
FABP6	0.836576587	0.648670639
HSD3B7	0.822041534	0.639060589
GMPPA	0.615156538	0.63768047
ANGPTL4	0.900408735	0.636615036
HK2	0.790649114	0.631149585
NNMT	0.815034959	0.630007906
FABP7	0.645043546	0.595688044
GAPDH	0.778735085	0.594986142
GUCY1B1	0.680588852	0.569313097
DEGS1	0.683273293	0.557397866
TRIB3	0.712837154	0.556694279
PLOD1	0.736975862	0.551596435
PFKP	0.873385501	0.548209561
SLC2A1	0.780303844	0.545673823
ALDOA	0.754759406	0.545563098
BCKDK	0.844555219	0.545326261
P4HA2	0.614027427	0.526402019
FUT11	0.737069935	0.520199837
GAL3ST1	0.875238428	0.518778632
CHST14	0.707763947	0.518665353
NT5C3A	0.648733685	0.51730359
GUCY1A1	0.693272441	0.515722187
CA9	0.846740147	0.514933622
DARS1	0.831192647	0.51276856
TPI1	0.72704085	0.508953119
SHMT2	0.803666376	0.506210051
S100A10	0.515789877	0.503575768
ABCA1	0.768897451	0.502356547
PLA1A	0.522964185	0.501730326
ENO2	0.692419923	0.498988813
RUFY1	0.564672507	0.495738616
SLC27A3	0.643069717	0.494681868
NBN	0.541212192	0.493567053
ENPP3	0.77659808	0.488384011
CSPG4	0.732277853	0.481830794
HILPDA	0.870109607	0.481085808
DGKD	0.723200576	0.478070378
PPARD	0.65206639	0.471103682

PYGL	0.763160801	0.465925828
GALNT14	0.794178109	0.46407584
ABCC3	0.777193863	0.459253777
GXYLT1	0.443831424	0.457279457
PFKFB4	0.712958614	0.452311274
MTRR	0.727201521	0.450616145
PHKA2	0.84117867	0.436103648
MINPP1	0.512771748	0.436091363
INSIG2	0.692158375	0.435941069
CHD9	0.469344059	0.423142023
SCD	0.691782076	0.41532694
TYMS	0.597843557	0.400831603
PLIN2	0.764498358	0.394781035
MGLL	0.670463515	0.39375434
PHYKPL	0.696971703	0.393636864
MAN1A2	0.37617859	0.393183648
NR1H3	0.647133661	0.389994658
POMGNT1	0.545589641	0.378315389
PGM1	0.580230791	0.369414475
NMRAL1	0.518137643	0.35655573
ALDOC	0.653186904	0.352119535
COX4I2	0.511324702	0.338097695
MIF	0.627634155	0.335184818
AMDHD2	0.42100931	0.328266877
PPP1R3C	0.590845901	0.30580589
PGK1	0.545142214	0.303568376
B3GNT4	0.49838564	0.299922602
LPIN3	0.572660699	0.296879641
RIMKLA	0.703014498	0.28497321
PLCB1	0.530989294	0.283799601
KMO	0.41901701	0.283745092
PTPRG	0.346093605	0.282374895
RRM2B	0.388593541	0.254048601
ARSA	0.411055634	0.253438029
SLC6A8	0.540689099	0.236697004
MVK	0.477845077	0.231979796
CYB5R3	0.583342914	0.225698644
SLC45A2	0.367760806	0.182197274
GPI	0.573342716	0.171578804
NT5C	0.517405644	0.154602698
NT5C3B	0.344561773	0.154266715
HYI	0.373037183	0.138265977
FITM1	0.35579937	0.133306853
RPS15	0.454754794	0.053139679
MED26	-0.356048682	-0.168315316
ACSL4	-0.556577726	-0.191173288
PSME3	-0.599111515	-0.225357279
PSMA3	-0.464909887	-0.227111384
MED28	-0.409089303	-0.236834464
HS3ST1	-0.569453041	-0.243769627
MTMR14	-0.449756137	-0.247953556
PAPSS1	-0.475929645	-0.254195625
PSMD6	-0.662001594	-0.261305581

ADSS2	-0.646668324	-0.276760697
ITPKB	-0.504684007	-0.300715214
MED27	-0.702282075	-0.30690318
SRR	-0.570256794	-0.30730656
ACSL3	-0.683540306	-0.307784937
PDE3B	-0.477353988	-0.308757372
PGD	-0.606594803	-0.316678311
IDO2	-0.452562425	-0.322946564
PPP1CA	-0.567216802	-0.326916079
MTMR1	-0.443193148	-0.334643775
GLB1	-0.702279406	-0.339727525
MBOAT1	-0.627731556	-0.348892411
METAP1	-0.521711012	-0.35277597
GALNT7	-0.325380626	-0.363408151
OAT	-0.615154697	-0.369472115
ACSL6	-0.361317224	-0.373035681
PSTK	-0.491497875	-0.393211803
ABHD5	-0.636592013	-0.396594654
SETD2	-0.673758083	-0.404881462
LIPG	-0.584652913	-0.405126936
NT5C2	-0.737754942	-0.406243196
ADH1B	-0.575155334	-0.421700295
QARS1	-0.646212167	-0.436408075
SMARCD3	-0.701556651	-0.439180065
RPL15	-0.70157321	-0.441043306
GPC3	-0.734401569	-0.455137398
ADK	-0.654228928	-0.462250517
MAN1C1	-0.713744643	-0.46413307
MED20	-0.823414727	-0.481259216
GMDS	-0.61425517	-0.483276843
ADH1A	-0.737511165	-0.486666708
CGA	-0.591093799	-0.492023025
PCCB	-0.721226189	-0.507127162
TSPOAP1	-0.318122657	-0.509368017
HACL1	-0.760479585	-0.50997318
SPTLC2	-0.60398001	-0.512516681
PIK3C2G	-0.688490455	-0.515751447
LYPLA2	-0.636855316	-0.516155692
POMGNT2	-0.699814374	-0.51633317
SCAP	-0.684424629	-0.526518052
GALNT16	-0.696261786	-0.570326284
DPM3	-0.676334395	-0.587329354
MECOM	-0.853946023	-0.592125757
PDHB	-0.88495553	-0.655111837
PLCD1	-0.749853209	-0.699382992
AK3	-0.768174504	-0.724241047
