# Supplementary material for: Comprehensive analyses of A 12-metabolism-associated gene signature and its connection with tumor metastases in clear cell renal cell carcinoma
Source: BMC Cancer. 2023 Mar 23;23:264. doi: 10.1186/s12885-023-10740-6 (PMC10035225; doi:10.1186/s12885-023-10740-6)
Supplement: Supplementary file 5 — Additional file 5: Supplementary Table S4. The 13 genes from LASSO regression and their proportional hazards assuption results in multivariate Cox regression. [file 12885_2023_10740_MOESM5_ESM.pdf]

Supplementary Table S4: the 13 genes from LASSO regression and their proportional hazards assuption results in multivariate Cox regression

Gene ID	Coefficients from LASSO regression	Test the proportional hazards assumption		
		Score test of for addition of the time-dependent term	The degrees of freedom	*P*
ENO2	3.51E-04	0.6984	1	0.403
GALNT14	-3.92E-04	0.9339	1	0.334
GALNT7	7.76E-03	1.1622	1	0.281
GMPPA	4.72E-03	0.754	1	0.385
HYI	6.32E-03	2.2005	1	0.138
ITPKB	-1.70E-03	0.0583	1	0.809
LPIN3	2.04E-04	6.4956	1	0.011
METAP1	-7.63E-03	0.106	1	0.745
PFKP	-2.96E-04	0.1434	1	0.705
PLIN2	-5.17E-05	0.3659	1	0.545
PLOD2	3.33E-04	1.9092	1	0.167
RIMKLA	-1.32E-02	2.2866	1	0.13
TRIB3	7.19E-04	0.7299	1	0.393
